# A Triassic crown squamate

**DOI:** 10.1126/sciadv.abq8274

**Published:** 2022-12-02

**Authors:** David I. Whiteside, Sofía A. V. Chambi-Trowell, Michael J. Benton

**Affiliations:** ^1^ School of Earth Sciences, University of Bristol, Bristol, BS8 1RJ, UK.; ^2^ Palaeontology Section, Earth Science Department, The Natural History Museum, Cromwell Road, London SW7 5BD, UK.

## Abstract

Mammals, birds, and squamates (lizards, snakes, and relatives) are key living vertebrates, and thus understanding their evolution underpins important questions in biodiversity science. Whereas the origins of mammals and birds are relatively well understood, the roots of squamates have been obscure. Here, we report a modern-type lizard from the Late Triassic of England [202 million years (Ma)], comprising a partial skeleton, skull, and mandibles. It displays at least 15 unique squamate traits and further shares unidentatan and anguimorph apomorphies. The new discovery fixes the origin of crown Squamata as much older than had been thought, and the revised dating shows substantial diversification of modern-type squamates following the Carnian Pluvial Episode, 232 Ma ago.

## Teaser

The world’s oldest modern-type lizard is reported from the Late Triassic of England, 34 million years older than presently known.

## INTRODUCTION

Dating the origins of modern species-rich groups is crucial for understanding macroevolution. This is a focus for the “tree of life” program because correctly dated phylogenetic trees provide insights into origins of biodiversity, innovations in evolution, and the impact of environmental drivers on macroevolution. Dating squamate origins, a clade with more than 11,000 extant species (*1*), has proved problematic, with estimates as latest Early Jurassic [~175 million years (Ma)] from fossils (*2*–*5*), Late Triassic (~205 Ma) from molecular data (*6*), and Triassic-Jurassic (172 to 213 Ma) from combined data (*7*, *8*). The oldest examples of modern squamates are incomplete Middle Jurassic fossils [~168 Ma; (*2*–*5*)], and the first articulated skeletons occur in the Late Jurassic Solnhofen Formation [146 to 151 Ma; (*9*)].

Earlier squamates, from the Triassic, should be expected, partly because molecular data point to older origins, and because the sister clade of Pan-squamata, Rhynchocephalia, includes diverse Late Triassic examples (*2*, *7*). Several Triassic lizard-like animals have been described and analyzed (*10*, *11*), but they are either Pan-lepidosauria or Lepidosauria (e.g., *Taytalura*) or Pan-squamata (e.g., *Sophineta* and *Megachirella*) and do not belong to the crown clade Squamata.

Clarity in group terminology is essential (*12*, *13*). The widest clade is Pan-lepidosauria, including Lepidosauria plus all fossil taxa closer to modern lepidosaurs than to extant crocodilian and avian archosaurs. Lepidosauria comprises Rhynchocephalia (*Sphenodon* plus extinct relatives) plus Pan-squamata. Pan-squamata (*12*) includes stem reptiles that are closer to squamates than to rhynchocephalians. “Squamata” is used here strictly for the crown clade, namely, all the living species plus their ancestors back to their last common ancestor [(*13*); see the Supplementary Materials].

Here, we report an exceptional, well-preserved skull and skeleton of a Late Triassic lizard, a crown squamate, extending modern squamate origins back by >30 Ma. The key specimen, studied through micro–computed tomography (CT) scanning, comprises the skull, mandibles, vertebral column, pectoral girdle, and forelimbs, representing a single individual.

## RESULTS

### Formal establishment of *Cryptovaranoides microlanius* n. gen., n. sp.

Infraclass DIAPSIDA Osborn, 1903 (*14*)

PAN-SQUAMATA sensu Gauthier and de Queiroz, 2020 (*12*)

SQUAMATA sensu de Queiroz and Gauthier, 2020 (*13*)

*Cryptovaranoides* gen. nov

Figures 1 to 7 and fig. S1

**Fig. 1.**
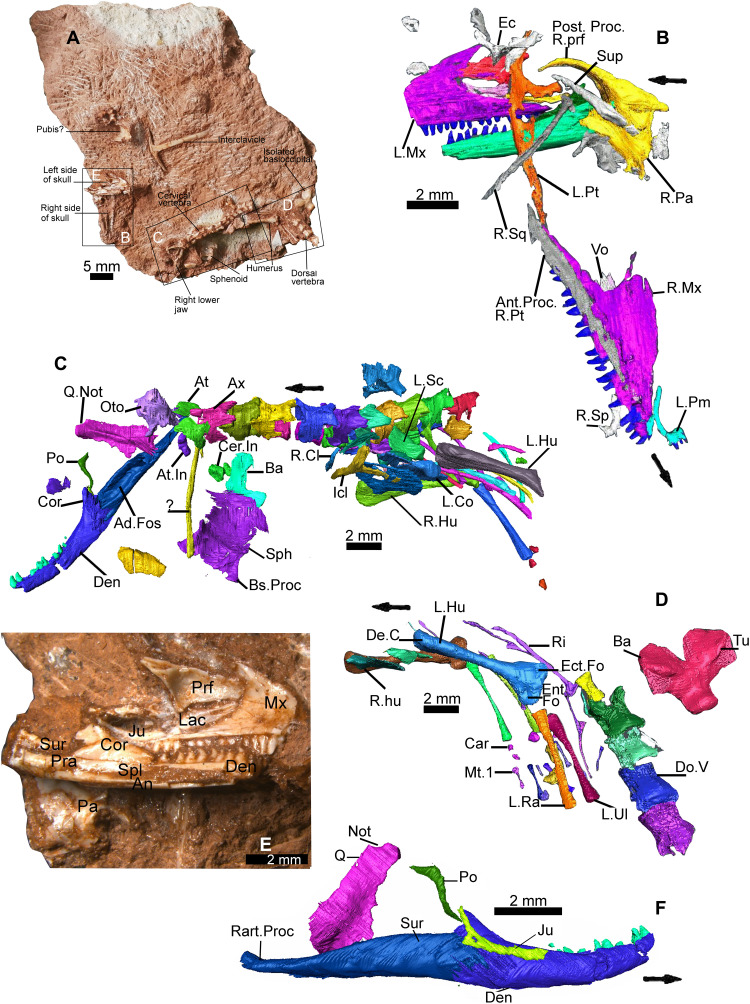
*C. microlanius* (NHMUK PV R36822), anatomical details. (**A**) Rock slab exhibits articulated partial left side of skull and mandible in medial view next to articulated anterior skeleton. (**B** to **D**) Segmented CT scan models with regions outlined in (A); (B) is obverse side to (A). (**E**) Close-up of the left-hand side of the skull in medial view. (**F**) Lateral view of CT-scanned right mandible. Arrow points to anterior in this and subsequent figures. 2nd, secondary; Ad, adductor; An, angular; Ant, anterior; At, atlas; Ax, axis; Ba, basioccipital; Bs, basipterygoid; C, crest or crista; Car, carpal; Cer, cervical; Cl, clavicle; Co, coracoid; Cor, coronoid; De, deltopectoral; Den, dentary; Do, dorsal; Ec, ectopterygoid; Ect, ectepicondyle (ectepicondylar); Ent, entepicondylar; Fo, foramen (foramina); Fos, fossa; Hu, humerus (humeral); In, intercentrum; Inc, interclavicle; Ju, jugal; L., left; Lac, lacrimal; Mt., metacarpal; Mx, maxilla (maxillary); Not, notch; Oto, otoccipital; Pa, palatine; Pm, premaxilla; Po, postorbital; Post, posterior; Pra, prearticular; Prf, prefrontal; Proc, process; Pt, pterygoid; Q, quadrate; R, right; Ra, radius; Rart, retroarticular; Ri, rib; Sc, scapula; Sp, septomaxilla; Sph, sphenoid; Spl, splenial; Sq, squamosal; Sup, supratemporal; Sur, surangular; Tu, tubera; Ul, ulna; V, vertebra(l); Vo, vomer.

**Fig. 2.**
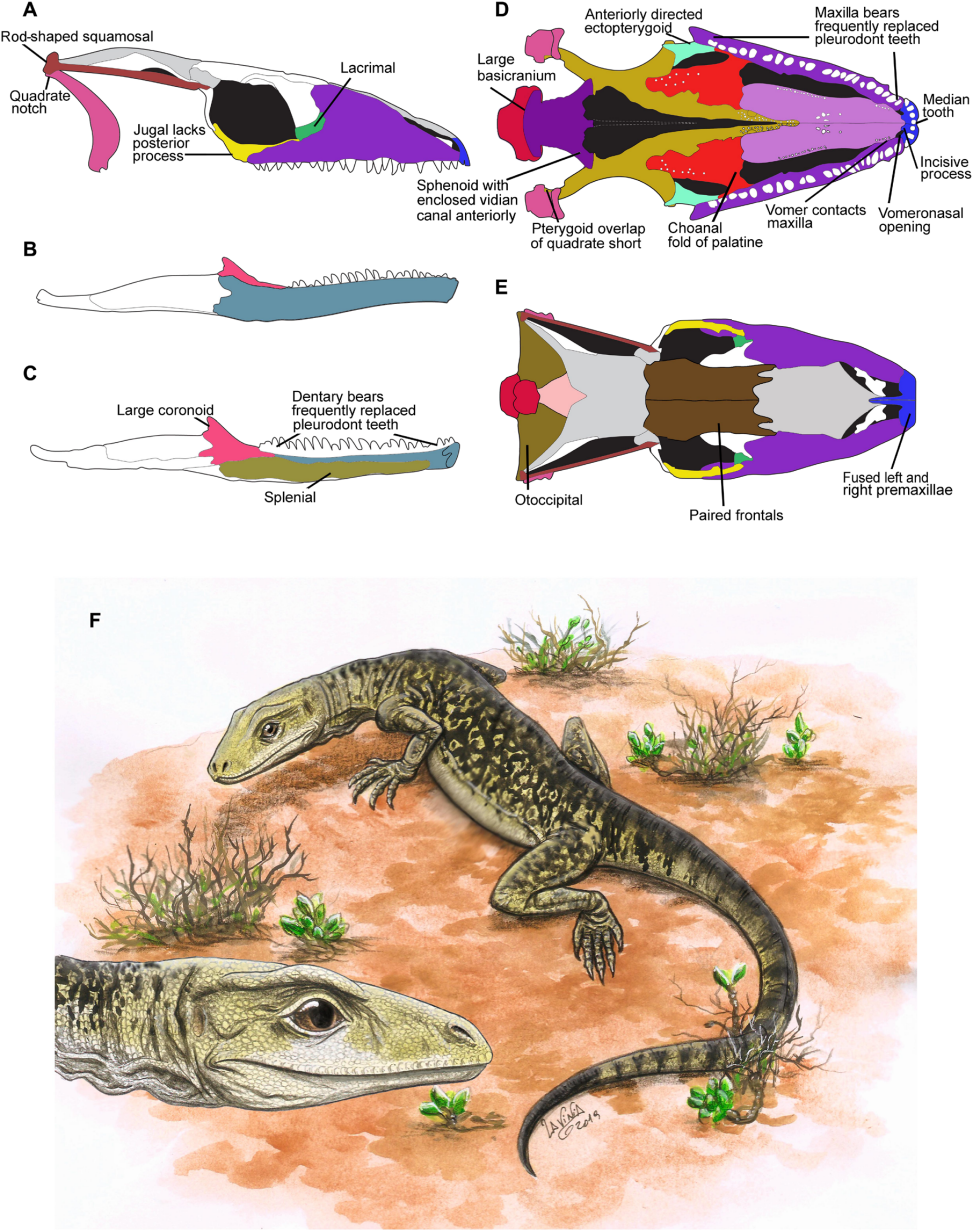
Reconstructed skull and mandible of *C. microlanius*, based mainly on the holotype PV R36822. Some key squamate features are labeled. Bones in light gray (nasal, parietal, and postfrontal) are unknown and speculatively reconstructed. Skull shown in lateral (**A**), ventral (**D**), and dorsal (**E**) views; right mandible in lateral (**B**), left mandible in medial (**C**) views. (**F**) Life restoration by L. Gandolfi. Estimated skull and mandible length is 14 mm for this juvenile specimen; isolated bones indicate that the skull can reach about 30 mm. Entire animal length ~ 25 cm. For more details of bones, see fig. S1. Outline drawings by S. Powell, University of Bristol.

**Fig. 3.**
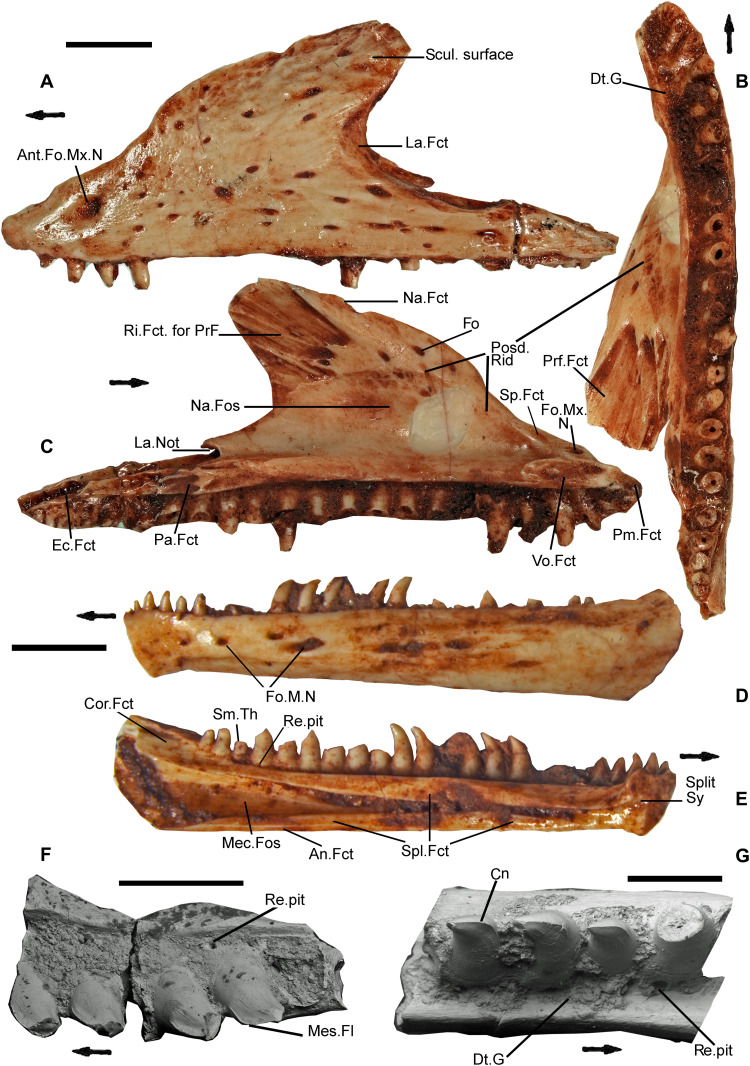
Maxillae and dentaries of *C. microlanius*. (**A** to **C**) Left maxilla (NHMUK PV R36999), the most complete specimen in the collection: (A) in lateral view, (B) in ventral view, and (C) in medial view. (**D** and **E**) Left dentary (NHMUK PV R37001) in lateral (D) and medial (E) views; note coronoid facet formed by a sulcus on dentary in (E). (**F**) Scanning electron microscopy image of right maxilla fragment (NHMUK PV R37280) and (**G**) fragment of left dentary (NHMUK PV R37282) demonstrating active replacement in pleurodont teeth. Scale bars represent 0.5 mm for (G), 1 mm for (F), and 2 mm for all other bones; top bar is for (A) to (C); middle bar is for (D) and (E). Abbreviations as for Fig. 1 and Cn, carina; Dt,g, dental gutter; Fct, facet; La, lacrimal; M, mandibular; Mec, Meckelian; Mes, mesial; N, nerve; Na, nasal; Posd, posterodorsal trending; Rid, ridge(d); Re, resorption; Scul, sculptured; Sm, small (smaller); Sy, symphysis (symphyseal).

**Fig. 4.**
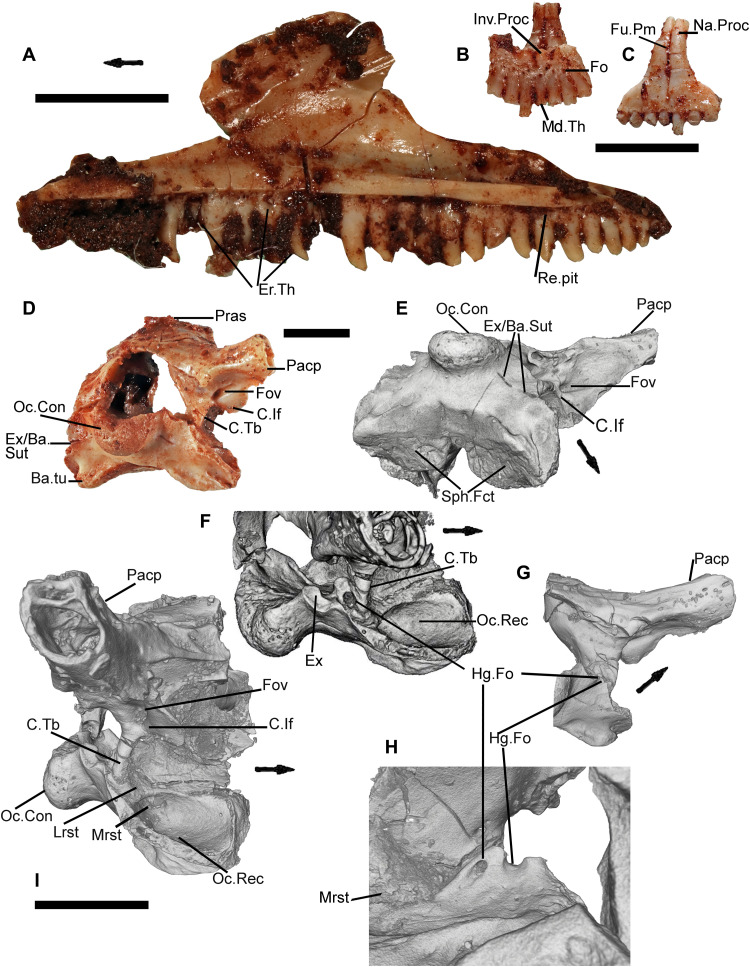
Isolated elements of *C. microlanius* showing squamate features. (**A**) Medial view of right maxilla NHMUK PV R37279 with recurved mid and posterior teeth, resorption pits, erupting teeth on many pleurodont emplacements. (**B** and **C**) Fused premaxilla NHMUK PV R37378 in postero-palatal view (B) showing incisive processes, foramina, and characteristic asymmetrical number of teeth, 4 on right premaxilla, 3 on left, and anterior view (C). (**D**) Right postero-lateral view of braincase NHMUK PV R37377 showing oto-occipital, ascending process of supraoccipital and laterally expanded paroccipital process. (**E**) Ventral CT scan view of same braincase with similar basioccipital to holotype. (**F**) Lower part of braincase in lateral view showing damaged exoccipital where vagus foramen should be (posterior to crista tuberalis but anterior and above hypoglossal foramen). (**G**) Posterior CT scan view of right side showing damaged exoccipital; lines show position of (H). (**H**) Close-up, in lower posterior medial view showing hypoglossal foramina on damaged exoccipital part of otoccipital. (**I**) CT scan of otoccipital in lateral view showing squamate features: lateral opening of recessus scala tympani (label to opening), medial opening of recessus scala tympani, crista tuberalis, crista interfenestralis, and occipital recess. All scale bars, 2 mm [bottom bar is for (I)]. Image (H) is 0.75 mm across. C, crista; Con, condyle; Er, erupting; Ex, exoccipital; Fov, fenestra ovalis; Fu, fused; Hg, hypoglossal; If, interfenestralis; Inv, incisive; Lrst, lateral opening of recessus scala tympani; Md, median; Mrst, medial opening of recessus scala tympani; Oc, occipital; Op, opening; Pacp, paroccipital process; Pras, processus ascendens; Rec, recess; Sut, suture; Tb, tuberalis; Th, tooth.

**Fig. 5.**
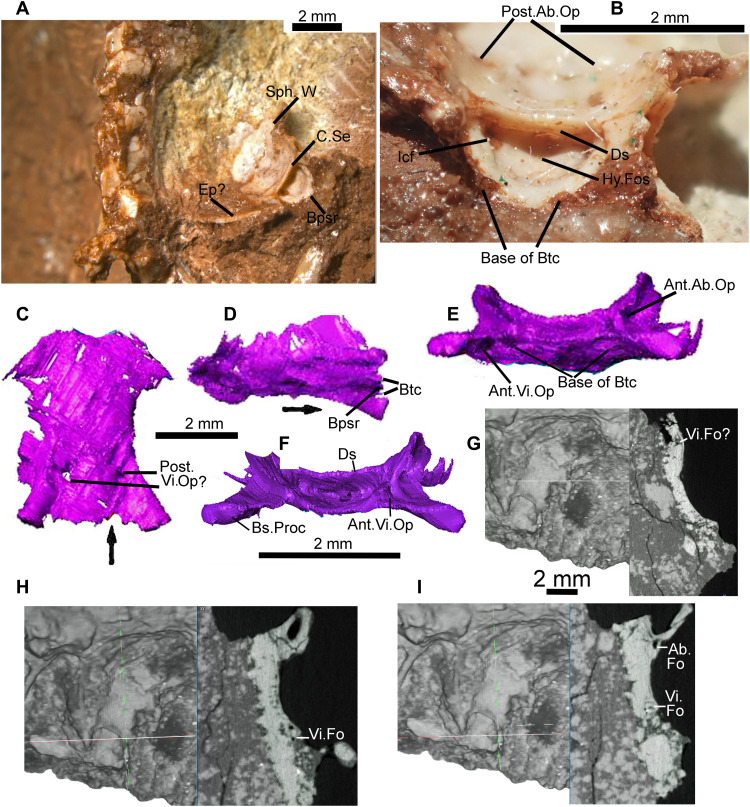
Images of the holotype of *C. microlanius* showing sphenoid and indicating squamate features. (**A**) View of holotype highlighting the sphenoid with a prominent dorsum sella (showing a deep fossa) and high crista sellaris. (**B**) Close-up anterior (and slightly dorsal) view of the sphenoid showing posterior opening of the abducens canal and internal opening of the carotid canal. (**C** to **F**) CT scan of sphenoid in (C) ventral, (D) right latero-ventral, (E) antero-ventral, and (F) anterior views. (**G** to **I**) CT cross sections on right of each image, with thin white line showing position on left-hand side, which is a dorsal view. (G) is about midway antero-posteriorly with the slice cutting across the lateral “wing.” (H) and (I) are at the anterior of the sphenoid; (I) is the most anterior. Squamate features in (C) to (I) show position of Vidian and Abducens canal foramina. All scale bars, 2 mm [bar for (C) and (D) between images; (E) and (F) bar under (F) and bottom bar is for (G) to (I)]. Ab, abducens; Bpsr, parasphenoid rostrum; Btc, trabeculae cranii; Ds, dorsum sella; Ep, epipterygoid; Hy, hypophysial; Icf, internal carotid foramen; Se, sellaris; Vi, vidian; W, wing.

**Fig. 6.**
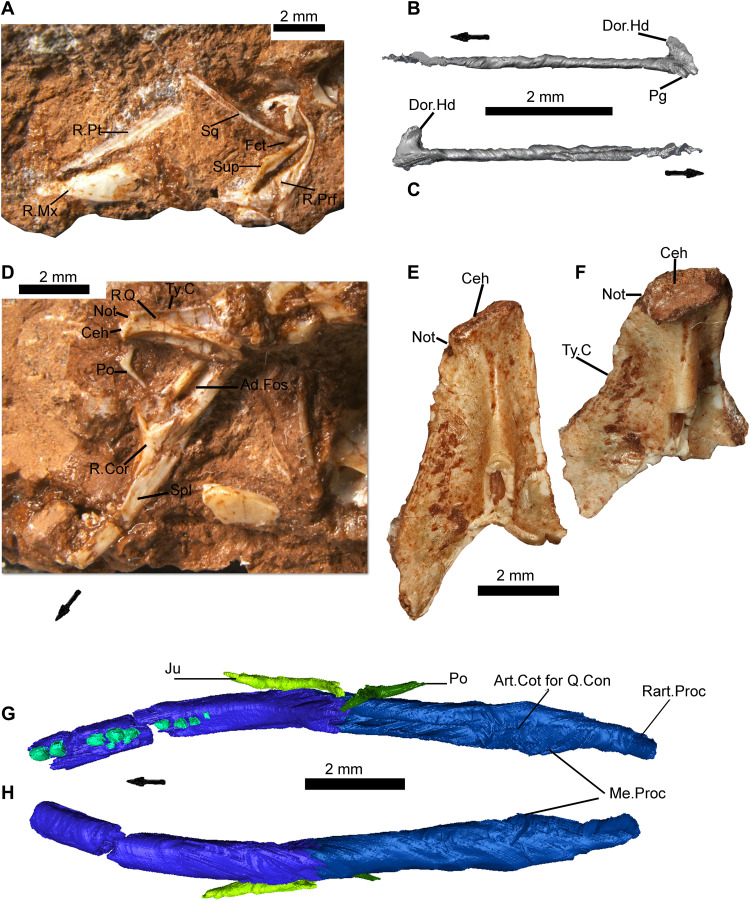
Images of the holotype of *C. microlanius* and isolated quadrate showing squamate features. (**A**) Reverse side of holotype fossil rock NHMUK PV R36822 shown in Fig. 1A. (**B** and **C**) Right squamosal in (B) medial and (C) lateral view. (**D**) Close-up of the right lower jaw and associated bones including the right quadrate and right postorbital. (**E** and **F**) Digtally separated from matrix isolated left quadrate NHMUK PV R37604 in (E) posterior and (F) dorso-posterior views. (**G** and **H**) Scan of right lower jaw of holotype NHMUK PV R36822 in (G) dorsal and (H) ventral views. All scale bars, 2 mm. Art, articular; Ceh, cephalic head; Cot, cotyle; Hd, head; Me, medial; Pg, peg; Ty, tympanic.

**Fig. 7.**
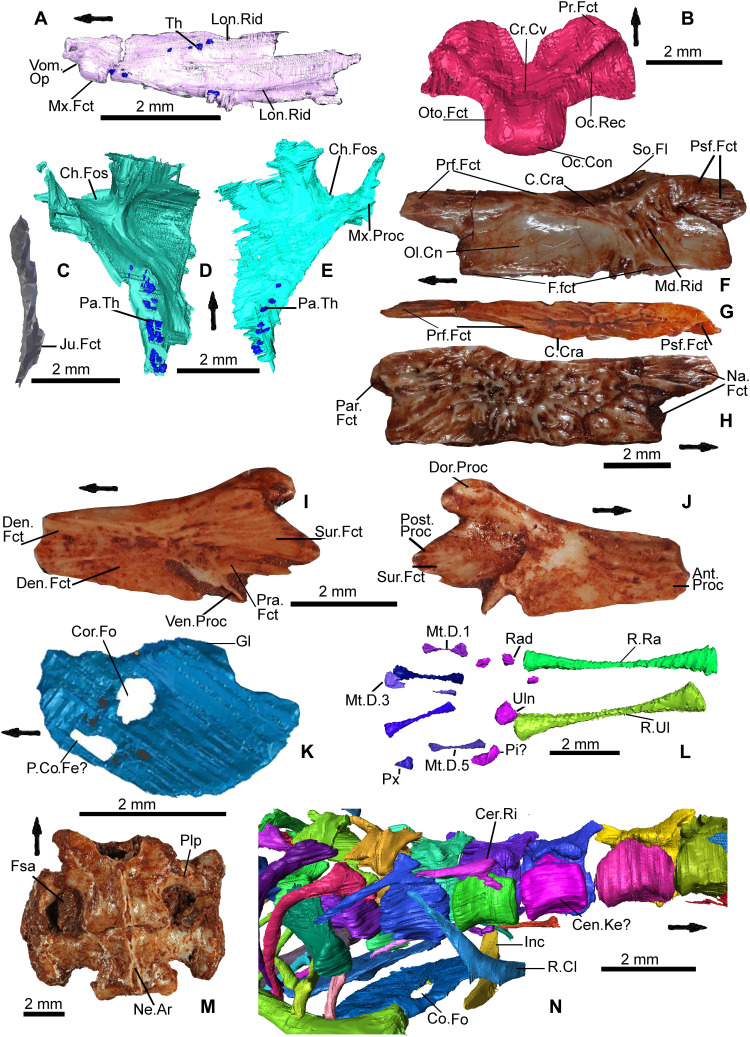
Important anatomical elements of *C. microlanius*. (**A**) Ventral view of right vomer showing ridges with teeth, border of the vomeronasal opening, and thickened anterior lateral side with facet for maxilla. (**B**) Isolated basioccipital in dorsal view showing occipital recess and facets for exoccipital (of the otoccipital) and prootic; about three times as large as the basioccipital next to the sphenoid of the articulated holotype. (**C**) Right postorbital in anterior aspect with the probable jugal facet identified. (**D** and **E**) Ventral views of right (D) and left (E) palatines; note that choanal fossa positioned anteriorly on each bone and two rows of teeth on posterolateral regions. (**F** to **H**) Isolated left frontal NHMUK PV R37274 in (F) ventral view showing crista cranii, olfactory canal and facets for the prefrontal and the (presumed) postfrontal, (G) lateral view showing facets for the prefrontal and (presumed) postfrontal, and (H) showing sculptured surface and facets for the nasal and parietal. (**I**) Isolated left coronoid bone NHMUK PV R37273 in lateral aspect; facets for the dentary, prearticular, and surangular are present as well as the ventral process. (**J**) Medial view of the same coronoid bone with conspicuous posterior, dorsal, and anterior processes as well as large fossa for the bodenaponeurosis. (**K**) Right coracoid, medial view, showing coracoid foramen and possible fenestra. (**L**) Right radius, ulna, carpus, and metacarpus. (**M**) Dorsal view of isolated sacral vertebrae NHMUK PV R37275 with fused pleurapophysis, foramen sacrale, and neural spine labeled. (**N**) Close-up of right side of cervical vertebrae showing ribs and pectoral elements including the interclavicle, clavicle, and coracoid with coracoid foramen labeled. Scale bars, 2.0 mm. Images (A) to (E), (K), (L), and (N) are segmented images from the CT scan of the holotype. Abbreviations: As in the Supplementary Materials, p. 48.

### Type species—*C. microlanius* sp. nov. (by monotypy)

Etymology—The genus name *Cryptovaranoides* is derived from the Greek word crypto, “hidden,” new Latin varan (from Arabic Waran meaning “dragon-lizard”), and the Greek suffix oides meaning “like.” The generic name is thus “hidden, lizard-like” (animal), referring to the fact that this fossil has remained unrecognized in a collection for nearly 70 years and that it was a small lizard-like animal (skull length maximum about 30 mm), living on karst limestone, perhaps hiding in the grykes (crevices). The species name *microlanius* derives from the Greek “micro” = small and Latin “lanius” meaning butcher. The recurved blade-like teeth demonstrate that the animal was a small efficient predator that could capture and immobilize prey quickly.

Holotype—NHMUK PV R36822, a partly articulated anterior skeleton and associated partial skull and lower jaws preserved on and in the red matrix of a small block of conglomeratic limestone. The left mandible and a partial skull (Fig. 1, A and B), including both maxillae and associated bones, are close to the articulated partial skeleton. The left side of the skull comprising the maxilla, prefrontal, jugal, and lacrimal in near-life positions (Fig. 1, B and E) has twisted away from the right side. The partial skeleton (Fig. 1C) includes the right mandible with associated quadrate, jugal, postorbital, otoccipital, basicranial bones, articulated cervical and anterior dorsal vertebrae, as well as pectoral girdle and forelimb elements. Nearby are further articulated dorsal vertebrae, followed by a basioccipital (Figs. 1, A and D, and 7B) from a larger individual of *Cryptovaranoides*. Bone preservation (Fig. 1, A and E) is superb and detail in the CT scans (Fig. 1, B to D and F) is often excellent, allowing us to identify numerous characters of modern squamates.

Referred fossils—Referred isolated bones (all NHMUK PV) are as follows: left maxilla R36999; right maxilla R37279; fragment of right maxilla R37280; left dentary R37001; fragment of left dentary R37282; right dentary R37281; left coronoid R37273; left frontal R37274; left quadrate R37604; braincase R37377; premaxilla R37378; sacral vertebrae R37275; cervical vertebra R37276; dorsal vertebra R37277.

Locality and age—Triassic fissure deposit in Carboniferous Limestone, Cromhall Quarry, Tortworth, Gloucestershire, United Kingdom. Age is Rhaetian, probably late Rhaetian (equivalent of Cotham Member, Lilstock Formation), 201.6 to 201.8 Ma (*15*).

Diagnosis—Characterized by the following apomorphies: (i) maxillary and dentary with frequently replaced conical, pleurodont recurved teeth (Figs. 1, A to C, E, and F, 2, C and D, 3, A to G, and 4A) displaying labio-lingually compressed apices (upper part of the crown) and a prominent sharp carina on mesial and distal edges (Fig. 3, F and G). The mesial compressed region is somewhat more pronounced than the distal, and overall cross section of compressed region is oculiform-lanceolate. The mesial region of mid and posterior maxillary teeth develops a pronounced blade-like flange. The lingual surface of the dentary and maxillary teeth has 2 to 10 vertical striae (higher number on younger, smaller unworn teeth), including a few branching striae (observed on dentary teeth), on compressed upper part of crown; (ii) tripartite maxilla (Figs. 1B, 2A, and 3, A to C, and fig. S1, A and F) with high facial process that curves medially encompassing a prominent nasal fossa bordered dorsally with a distinct ridge; also, there are long anterior and posterior processes; (iii) lateral faces of dentary and maxilla are perforated by many foramina (Fig. 3, A and D; *c* 20+ for the large maxillae) for branches of the mandibular and maxillary nerve (CN5), respectively, with a range of sizes; about 20% of maxillary foramina have maximum length greater than observable lateral tooth width; mandibular foramina usually form double row anteriorly; (iv) premaxillae fused in larger individuals (Fig. 4, B and C) with four teeth on one premaxilla and three on the other; (v) the fused premaxillae dentition includes a median tooth derived from the premaxilla with the greater number of teeth; (vi) the sphenoid contains a vidian canal (Fig. 5, E and F) that is enclosed as it passes over the basipterygoid processes and an internal carotid foramen that opens into a deep hypophysial fossa (Fig. 5B) bounded posteriorly by the dorsum sellae; (vii) pronounced dorsum sella roofed by a prominent well-developed anteriorly directed crista sellaris that partially covers the fossa (Fig. 5, A and B); (viii) abducens canal present (Fig. 5, E and I); (ix) metotic fissure subdivided with recessus scala tympani developed anteriorly (floored by a prominent occipital recess) bounded posteriorly by the crista tuberalis (Fig. 4, D to H); (x) long thin squamosal with dorsal and posterior processes (Figs. 1B, 2A, and 6, B and C, and fig. S1, A, D, and F), the latter articulated as a “peg-in-socket” with the notch on quadrate; (xi) quadrate with notch for squamosal on lateral side of cephalic condyle (Figs. 1, C and F, and 6, D to F); (xii) jugal: posterior aspect very short and truncated with straight edge; short dorsal process and longer anterior process (Fig. 1F); (xiii) postorbital forms a slender postorbital bar with the dorsal process of the jugal (Figs. 1F, 2A, and 7C, and fig. S1, A and F); (xiv) coronoid is relatively large (Figs. 1E, 2C, and 7, I and J, and fig. S1, B and C); about 20% of lower jaw length and more than 80% of jaw height (measured dorso-ventrally from top of coronoid process to ventral base of mandible) in medial view and with a prominent posterior recess, presumably for the bodenaponeurosis; (xv) vomer (Fig. 7A) is in anterior sutural contact with maxilla (Fig. 3C shows vomer facet on the maxilla), separating the vomeronasal opening from the choana; (xvi) ventrally the vomer has two prominent postero-anterior longitudinal toothed ridges that somewhat converge anteriorly (Fig. 7A).

### Skull

The reconstructed skull (Fig. 2, A to E, and fig. S1) is based largely on the holotype, augmented by isolated bones of larger individuals. The holotype is a juvenile, based on its small size (skull length, 14 mm) and unfused cervical neural arches and centra. Isolated bones suggest that an adult skull could be 30 mm long and up to 16 mm wide. The reconstruction started with the palate (Fig. 2D) because we have all elements, with small, conical teeth, on the palatine, on longitudinal ridges of the vomer, and on the pterygoid anterior ramus; teeth are absent from the sphenoid, ectopterygoid, and most of the pterygoid. The proportions of the skull viewed laterally (Fig. 2A) and dorsally (Fig. 2E) follow the ventral view (Fig. 2D), and elements in color, except the frontals and braincase, are based on the three-dimensional (3D) CT scans. These indicate broad skull roof bones and evident streptostyly (Fig. 2A), with a narrow, conch-shaped quadrate, a thin rod-shaped squamosal, and the lower temporal opening is wide with a small, truncated jugal.

The palatal reconstruction (Fig. 2D and fig. S1E) is the most reliable as we have all the bones (but sometimes only the left or right element) from the palate represented in the CT scan images. The palatine, vomer, and anterior ramus of the pterygoid all bear small conical teeth in the anterior and mid-palate. Teeth are absent from the sphenoid and ectopterygoid, and there are no signs of them on the mid- or posterior parts of the pterygoid and its flange. There is a fossa for the base of the epipterygoid on the dorsal surface of the mid-region of the pterygoid (the fossa columellae; fig. S1H), but the CT scan is not distinct enough to ascertain its shape. Therefore, neither do we know the shape of the base of the epipterygoid. There is a possible epipterygoid (labeled “?” in Fig. 1C). However, this is a speculative identification as that element is in two parts, with one part about the right size for an epipterygoid in this skull. If the element was originally a complete unit (rather than from two different bones), then this “epipterygoid” must be some other unidentified bone.

The sphenoid forms the larger element of the basicranial region that constitutes a relatively large feature of the skull in ventral view. The other bone is the basioccipital with a maximum width, across the basioccipital tubera, of more than 42% the width between the lateral margins of the quadrate condyles. These proportions are similar to many lizards [calculated from skulls in (*16*, *17*)] such as *Varanus salvator* (overall between 33 and 80% for lizards and usually about 37 to 50% but can reach 80% in, e.g., the skink *Typhlosaurus*) compared to the smaller proportions found in rhynchocephalians such as *Sphenodon* (c. 30%), *Planocephalosaurus* [calculated at c. 27% from (*18*)], *Clevosaurus* [calculated at c. 25% from (*19*)], *Diphydontosaurus* [calculated at c. 20% from (*20*)], and *Gephyrosaurus* [calculated at c. 22% from (*21*)]. The subtemporal fenestra is relatively large and indicates that the adductor musculature was substantial.

The sphenoid is a thin bone, formed from the fused endochondral basisphenoid and the dermal parasphenoid (*17*); this was difficult to process from the CT scan because it is highly fractured, crushed in many places, with some missing bone, and sediment has filled the interstices. This is almost certainly a consequence, at least in large part, of the juvenility of the animal in life and therefore incomplete ossification. However, with additional close-up photography, the major features are shown in Fig. 5 (A to I). The base of the parasphenoid process lies between the trabeculae cranii (Fig. 5D), which diverge posteriorly forming the floor of the deep hypophysial fossa (Fig. 5, A and B), either side of which is a large foramen for the internal carotid artery (Fig. 5B). The hypophysial fossa is bounded posteriorly by a pronounced dorsum sella, and this is roofed by a prominent well-developed anteriorly directed crista sellaris that partially covers the fossa (Fig. 5, A and B). The sphenoid contains the closed vidian canal with the anterior opening at the base of the basipterygoid process (Fig. 5, E and F). The vidian canal, incorporating the internal carotid artery and the base of the palatine artery, enclosed by the sphenoid as it passes over the basipterygoid process is a squamate apomorphy (*22*). The position of the posterior opening is more difficult to discern. There is a foramen positioned on the lateral “wings” of the sphenoid (Fig. 5G) somewhat behind the posterior base of the basipterygoid process that may be toward the posterior entry. However, because of repeated fractures and some crushing and missing bone, it is not possible to follow this foramen continuously toward the front of the sphenoid, although there are enclosed foramina in the anterior region of the bone (Fig. 5, H and I). It is possible that the enclosed vidian canal is quite short and the posterior entry may be as suggested in Fig. 5C. The anterior exit of the abducens canal (Fig. 5E) is confirmed from the CT slice across the anterior of the sphenoid (Fig. 5I). The posterior opening of the abducens canal is more problematic to identify but is suggested to be as shown in Fig. 5B.

The vomers form a large portion of the palate and are bounded laterally by extensive choanae. We have searched carefully for facets on the posterior side of the premaxillae or the anterior margin of the vomer (Fig. 7A), but they appear to be lacking in the juvenile and an isolated vomer is absent in the larger bones of the collection. The anterior region of the vomer imaged from the CT scan has a small, truncated process positioned medially, which probably formed the connection to the premaxilla. The large, fused premaxillae (Fig. 4, B and C) is important as it is composed of a right bone with four teeth and a left bone with three teeth. The fusion of the two bones results in the most mesial tooth of the right premaxilla forming a median tooth; this is a synapomorphy of modern Squamata. The asymmetry in number of tooth positions of the two premaxillae is also found in the juvenile holotype.

Although not discernible on the juvenile holotype, *Cryptovaranoides* has incisive processes on a larger specimen of the premaxillae (Fig. 4B), and the connection with the vomer was probably ligamentous. An important aspect of the palate is the connection between the maxilla and the vomer in the most anterior region; the vomerine facet, which is ridged, is distinct above the subdental shelf of the maxilla (Fig. 3C), and the CT scan image of the vomer (Fig. 7A) has a clear laterally positioned and matching maxillary facet. The vomer facet on the maxilla demonstrate that the maxilla-vomer connection was strong and not simply a vomeromaxillary overlap. These positions result in a small gap on both lateral sides of vomer where it would have contacted the premaxillae. As there appears to be no palatal posterior process of the premaxillae, which would (in squamates) often border the vomeronasal fenestra on the medial side, we suggest that these small gaps are the external openings of the vomeronasal openings. There is a small emargination and a groove on the lateral side of the vomer at the anterior end, which we suggest is the vomeronasal notch (Figs. 2D and 7A). A vomeronasal opening bordered by the vomer posteromedially and the maxilla laterally is common in extant squamates, but an anterior premaxilla margin is rare. However, just such an arrangement of bones occurs in the amphisbaenians *Amphisbaena alba*, which has a similarly small external vomeronasal opening, and *Diplometopon* (*23*). We can also compare *Cryptovaranoides* with, for example, *Varanus* (*24*, *25*) where the vomeronasal opening lies anterior to the vomer/maxillary suture and is therefore separate from the choana. No other constriction (which might suggest an alternative position for the vomeronasal fenestra) in the choana is observed in *Cryptovaranoides*. We have therefore concluded that, like *Varanus*, *Cryptovaranoides* was neochoanate.

The hypothesized position of the vomeronasal opening is strengthened by the recognition of a right septomaxilla (Fig. 1B) that lies near the separated premaxillae and anterior of the disarticulated vomer and maxilla of the holotype. In life, the septomaxillae lay above and posterior to the external vomeronasal openings. The anterior chamber formed by the septum dividing the ventral space would have housed the Jacobson’s organ for chemoreception. This can be observed in the dorsal reconstruction (Fig. 2E and fig. S1D).

We have reconstructed the extent of the nasals (Fig. 2F) based on the facets of the maxillae, indistinct features on the postero-dorsal processes of the premaxillae, and (isolated) frontals. However, the dorsal reconstruction is less certain than the ventral view as CT scan images of the holotype do not show the nasal, frontal, postfrontal, or parietal. The parietal and nasal are shown as single elements analogously to *Varanus* but may well have been paired to match the frontals. On the basis of the lack of observable anterior nasal facets on the maxilla, we speculate that the anterior part of the olfactory chambers may have been unprotected by the nasal(s) and, if so, presumably would have been covered by thick toughened soft tissue instead.

There are isolated frontals (e.g., NHMUK PV R37274; Fig. 7, F to H), and we are reasonably sure that these are referrable to *Cryptovaranoides* because the lateral and ventral facets in the anterior and mid-region are complementary to prefrontal facets including those of the long curved posterior process. Also, like the CT scans of the prefrontal, the frontal bone is highly ornamented. The frontal bone (Fig. 7, F to H) has facets for the nasal anteriorly and prefrontal anterolaterally; these extend posteriorly along the lateral side. There are also posterior facets that lie laterally and ventrally for the (presumed) postfrontal [similar to those in *Elgaria*; figure 1.79 B of (*17*)] and a small underlap for the parietal posteriorly (Fig. 7H). The olfactory canal running along the ventral anterior half of the frontal is pronounced, but the crista cranii is relatively low as it is in teiids and iguanids (*17*) but less well developed in comparison to many squamates. The cartilaginous planum supraseptale would have attached to the crista cranii (*17*). In similar manner to iguanids, there is a median raised area at the end of the olfactory canal but, unlike that group, it is distinctively ridged in *Cryptovaranoides*. Palpebrals may have been absent or present, but we have no clear evidence either way.

The discovery of a good braincase NHMUK PV R37377 (Fig. 4, D to I), albeit missing the mid-region of the fused exoccipitals and some lateral bone, has allowed a reasonable understanding of this region of the skull with the paroccipital processes and the recognition of an otoccipital (fusion of exoccipital and opisthotic). On the right-hand side, the vagus foramen is inferred to lie above two foramina (Fig. 4, F and G) that we identify as hypoglossal foramina, which are positioned on the lower medial region of the broken exoccipital near its contact with the basioccipital. The hypoglossal foramina lie posteriorly to the medial opening of the recessus scala tympani (mrst). The crista tuberalis, which separates the anterior cavity of the metotic fissure (*10*, *17*), is present and protects the lateral opening of the recessus scala tympani (lrst; Fig. 4H). There was presumably a lateral vagus opening posterior to the crista tuberalis and anterior to the missing mid-region of the exoccipital, but the fossil provides no evidence. The occipital recess that floors the rst is observed clearly and on the separate basioccipital (Fig. 7B) with the same morphology, confirming that the braincase is from *Cryptovaranoides*. We confirm that the metotic fissure is divided as in squamates, based on the distinct and separate rst separated by the crista tuberalis (*10*), the (probable) vagus foramen likely positioned between the mrst and the hypoglossal foramina. Furthermore, where the metotic fissure is undivided as in *Sphenodon* (*17*), there is no occipital recess in the basioccipital, nor in basal sphenodontians and rhynchocephalians such as *Diphydontosaurus* and *Gephyrosaurus*, but it is pronounced in *Cryptovaranoides* (Fig. 4, F and I) as in many squamates. This braincase specimen has a partial suture line between the exoccipital part of the otoccipital and the basioccipital (Fig. 4, D and E), but the isolated basioccipital (Fig. 7B) and others in the collection indicate that fusion of the elements was uncommon. Fractures in the fossil (fig. S1D) probably indicate the suture with the supraoccipital. The supraoccipital has an ascending process (Fig. 4D), not extensive but clearly present.

There are difficulties in reconstructing the mid- to posterior region of the skull as it is uncertain whether the postorbital connected directly to the squamosal, frontal, and/or the parietal. If the facet for the jugal on the postorbital has been correctly recognized (Fig. 7C), then the bone size (relative to others) would suggest not. Moreover, posterolateral facets, partly positioned ventrally, on the frontal (Fig. 7F) indicate that a postfrontal was probably present. Also, there is a dorsal facet on the postorbital that indicates a suture to a bone, which is probably a postfrontal, missing from the fossil. A postfrontal is therefore likely to have connected the postorbital to the squamosal, but the evidence is not conclusive. The reconstruction of the position of the squamosal is based on its relative proportion to other preserved bones. It is probable that the long thin squamosal formed the lateral boundary of the upper temporal fenestra, and the supratemporal (also based on proportional size to other bones) would have formed part of the medial wall as in varanids. The dorsal process of the squamosal (Fig. 6, B and C) would have formed a buttress for the posterolateral region of the skull (*11*).

The same degree of uncertainty about the upper temporal region pertains to the lateral view. However, the two identical jugals and one postorbital (Fig. 1, C, E, and F) make it clear that the postorbital bar was very slender but probably complete (fig. S1, A and F). We have constructed the lower part of the quadrate based on isolated quadrates and the proportionate position of the cotyles within the lower jaw. The mid-region and snout reconstruction are more certain. We have been able to produce the same reconstruction with the CT scan images and another with the best-preserved isolated maxilla, NHMUK PV R36999 (Fig. 3A). The overall effect is of a skull with a long low profile (Fig. 2A and fig. S1, A and F). The proportions of skull bones in the holotype and from isolated bones suggest that the antorbital region is slightly longer than the postorbital, and we have reconstructed the skull based on this likelihood. However, without a parietal, there is some uncertainty, and the lengths of the antorbital and postorbital regions may have been more equal. The lower temporal fenestra is open and the jugal has only a small truncated posterior process. The lacrimal is similar in proportion (8% of the antorbital region in lateral view) to some living squamates such as *V. salvator* [(*17*), figure 1.89]. The maxilla has a profusion of foramina (for the maxillary nerve CN5) positioned all over the lateral surface of the bone (Fig. 3A and fig. S1A). This may indicate a highly sensitive integument over the region, which would have enabled the animal to sense prey in dark areas such as leaf litter or possibly indicates a nocturnal habit. However, the profuse foramina may perhaps rather relate to an amphibious mode of life as in the extant crocodile lizard *Shinisaurus crocodilurus*.

Without an identified nasal, we cannot be sure whether the reconstruction of the external naris is accurate, although the lack of an anterior nasal facet suggests that it would have been similar in life. The septomaxilla is exposed in our reconstruction and resembles that of varanids.

### Mandible

The mandible (Fig. 2, B and C) is well preserved on both sides (Fig. 1, C and F), showing all elements and numerous teeth. The lower jaw reconstruction (Fig. 2, B and C, and fig. S1, B and C) is based on the left-side element (Fig. 1, A, B, and E) and the 80% complete right-side specimen (Figs. 1, A, C, and F, and 6, D, G, and H) that can be viewed in situ medially and from the lateral (Fig. 1F), dorsal, and ventral sides in a CT scan image. One isolated dentary, NHMUK PV R37281, constitutes the other element in the reconstructions.

The holotype clearly shows where the surangular, angular, and prearticular meet the coronoid, splenial, and dentary. It also shows the relative positions of the splenial, angular, and dentary on the mid-anterior part of the mandible (Figs. 1E and 3E), confirmed by facets on the isolated bone NHMUK PV R37001. In addition, the isolated coronoid (Fig. 7, I and J) shows excellently preserved facets for the dentary, surangular, and prearticular. The presence of a splenial facet on another isolated coronoid guided us in our reconstruction of that part of the medial view. Our positioning of the posterior parts of the surangular, angular, and the prearticular is guided by features on the scans (Fig. 1, C and F) and is therefore an informed interpretation, but margins of the bones are not certain. The glenoid is also somewhat unclear, so the shape and structure of the articular surfaces is not as well defined as we would have liked. The condyles on the holotype quadrate are also relatively poorly preserved, but these are intact on some isolated elements to provide good evidence for the reconstructions.

The retroarticular is long and narrow, and there is a rudimentary medial (angular) process (Figs. 1C and 6, G and H) that lies medial and slightly posterior to the articular cotyles. The dorsal edge of the anterior lateral part of the retroarticular is presumably the ventral site for the tympanic membrane.

Viewed laterally and medially, the coronoid eminence is formed from the coronoid (Fig. 2B and fig. S1, B and C) only, rather than the dentary and surangular as in rhynchocephalians. In lateral view, the *Cryptovaranoides* coronoid forms about 41% of the dorso-ventral width of the mandible (measured from the upper apex of the coronoid to the ventral margin of the mandible); corresponding figures [calculated from (*16*, *17*)] in squamates are *Lialis* (31%), *Diploglossus* (39%), *Gonatodes* (39%), and juvenile *Gallotia* (41%), rising to the near 70% of the gecko *Pachydactylus*. In *Huehuecuetzpalli* (*26*), the sister taxon to the crown-Squamata, the calculated figure is about 27%, or in basal rhynchocephalians, e.g., *Gephyrosaurus* [calculated from (*21*)], it is 10%. The *Cryptovaranoides* coronoid bone, in medial view (fig. S1C), occupies about 84% of the dorso-ventral width of the mandible, as in the anguid *Diploglossus* (79%) or the gecko *Gonatodes* (80%), and it is higher than *Huehuecuetzpalli* (74%) and much greater than *Gephyrosaurus* (53%) and *Diphydontosaurus* [64%; calculated from (*20*)]. The ventral region of the coronoid lacks the arched shape found in many extant lizards, e.g., *Diploglossus* or the lacertoid *Gallotia*, but is similar to the base of the coronoid of the pygopodid *Lialis* or indeed in most snakes. The morphology of the *Cryptovaranoides* coronoid and its aspect on the lower jaw is therefore within the range of modern Squamates.

Laterally, the isolated dentary bones show many mental foramina (Fig. 3D), forming a lengthwise double row in places, particularly in the mid-dentary, and some of the foramina are large relative to the width of the jaw. This complements the multiple foramina on the maxilla and suggests that the animal had a highly sensitive integument covering the snout and the anterior of the lower jaw. It is plausible that this sensitivity was an adaptation for a semi-aquatic life to sense prey such as small arthropods and fish in the water. The “scincomorph” *Saurillodon marmorensis* (*5*) has a similar profusion of foramina on the anterior of the dentary.

### Dentition

Tooth implantation is pleurodont, within a pronounced subdental gutter and lingual wall (Figs. 1E, 3, B, C, and E to G, and 4A) with teeth firmly attached to the higher labial wall. Teeth are conical, slightly recurved, with a sharp unserrated carina.

The teeth show an iguanid-type regular replacement, with examples of a new smaller tooth emplaced lingually in the resorption pits of the older teeth (Fig. 4A). The marginal tooth-bearing bones of *Cryptovaranoides* show evidence of frequent tooth replacement, especially in the anterior half of the jaws. In about 25% of the isolated specimens of maxillae and dentaries, there are examples of infrequent replacement in posterior teeth, but the substantial bone of attachment (or cementum) present in basal rhynchocephalians such as *Gephyrosaurus* (*21*) or, particularly, *Deltadectes* (*27*) is not observed.

The middle and posterior teeth on both the dentary and maxilla are slightly larger than the anterior, and anterior teeth can be less recurved (Figs. 1E and 3, D and E). However, the dentition is essentially homodont along the dentary, contrasting with the heterodonty found, for example, in *Pseudopus* (*28*). The teeth are cone-shaped with a circular cross section toward the tooth base but prominently recurved and labio-lingually compressed in the upper apical half of the crown (Fig. 3G). There is greater variation in the maxillary dentition, with larger and more flanged teeth in mid-region (Fig. 3F). The teeth, unlike those of some varanids, are not serrated but have a sharp carina (resulting in a formidably sharp blade) running mesial-distally across the labio-lingually compressed crown apex of each tooth (Fig. 3, F and G). However, the apices of the mid and posterior maxillary teeth differ from the dentary teeth by the presence of a distinctive mesial flange (Fig. 3F), which produces a razor-blade edge. In profile, the dentary and anterior maxillary teeth closely resemble the recurved teeth of the anguimorphs *Anniella*, *Pseudopus*, *Heloderma*, *Shinisaurus*, or scincids such as *Typhlosaurus* and have some similarity (although the curvature is not as extreme) to those of boid snakes. The mid and posterior mesially flanged maxillary teeth of *Cryptovaranoides* resemble dentary teeth of *Ophisaurus ventralis*, displaying a mesial flange of the apex [(*28*), figure 12E], and may indicate a similar diet of arthropods, small vertebrates, and their eggs.

### Postcranial skeleton

The cervical vertebrae are well preserved in articulated series (Figs. 1C and 7L). The neural arches and pleurocentra are not fused, suggesting that the animal was a juvenile, in contrast with isolated adult cervical vertebrae in the collection where these elements are fused. Ribs are visible on some of the vertebrae. The scan shows probable bicapitate (= bicipital or dichocephalous) (*29*) ribs on CV6 and CV7 (Fig. 7N), and an additional image indicates another on CV5. Although modern Squamata are frequently considered to have the synapomorphy only unicapitate (= single headed) ribs [(*30*), Ch. 86], bicapitate cervical ribs are present in *Varanus* [(*29*, *31*), figure 1A]; Hoffstetter and Gasc [(*29*), p. 252] state that “a double articulation can often be seen in *Varanus* between the first cervical ribs and the corresponding vertebrae.” There are gaps between the vertebrae indicating that intercentra were present (but displaced in the specimen) on CV3 and posteriorly. Some images of bones on the scans are identified as intercentra (Fig. 1C).

There is a further series of dorsal vertebrae (Fig. 1D), but although they may well be from the same individual, they are not connected to the articulated anterior skeletal elements that reach the 11th vertebra in the holotype (Fig. 1C). There are individual vertebrae in the collection, some certainly and others probably belonging to *Cryptovaranoides*, which we can recognize by similarities in morphology. These bones have not only wider lateral processes and prominent zygapophyses but also rudimentary zygantrum and zygosphene accessory articulations.

There are a few isolated sacral vertebrae, of which the most complete is shown (Fig. 7M). The bones are characterized by broad pleurapophyses fused to the vertebrae. The sacrum consists of two vertebrae fused at the centra, the lateral ends of the pleurapophyses and probably at the neural arch. There is a large foramen sacrale between the pleurapophyses.

Both scapulae and coracoids (Figs. 1C and 7N) are present in the holotype. On the right coracoid, there are two large openings, one possibly the primary coracoid fenestra (Fig. 7, K and N) but that may be an artifact of the CT scan or a damaged area. The left scapula is shown in Fig. 1C. We have reconstructed the scapulocoracoid (fig. S1G) based on the holotype specimens, and this displays the anterior emargination between the scapula and coracoid. If the possible primary coracoid fenestra is excluded, our reconstruction bears most similarity to *Heloderma* among extant squamates. Left and right clavicles are imaged from the CT scan (Figs. 1C and 7N). The clavicle is thicker ventrally where it lies in a facet of the interclavicle. That distal part was orientated latero-dorsally, but the upper part was free of the interclavicle, thinner and twisted so that it curved dorsally and slightly posteriorly. In the holotype of *Cryptovaranoides*, the gracile interclavicle is essentially T-shaped (Fig. 1C) rather than anchor-shaped. There is no indication of an anterior process. The posterior process has been broken postmortem, so only a small part is present in the fossil.

Left and right humeri are represented in the holotype (Fig. 1, C and D). The distal end is notable for its large entepicondylar and ectepicondylar foramina with openings on the anterior and posterior sides. The left humerus of the holotype shows a proximal end bearing the deltopectoral crest. From CT scan images, the radius is slightly longer than the ulna (Figs. 1D and 7L), and there is a small protuberance distally, possibly a styloid process, on the left bone. There is no evidence of an epiphysis or a patella ulnaris in the ulna, perhaps because the holotype is a juvenile.

The carpals are somewhat spread out on the right wrist and include the ulnare, a probable radiale, and possibly the pisiform. There are small bones that might be distal carpals, some displaced to the distal ends of the metacarpals, but they cannot be identified further. All five metacarpals are seen beyond the extremities of the right forelimb epipodials (Fig. 7L), but metacarpal 3 is damaged and lacks the middle section. Metacarpals 2 and 4 and probably 3 are similar in length, but they are about 20% longer than metacarpal 5, which is slightly longer than metacarpal 1. Metacarpal 1 has an expanded proximal head, which suggests, as for squamates, that distal carpal 1 has fused with the proximal head of the metacarpal. No clear elements beyond the distal ends of the metacarpals have been recovered in the CT scan, so we are unable to make any substantial comment on the phalanges.

### Phylogenetic analysis

A serious problem at present in any phylogenetic analysis of fossil Lepidosauria or Squamata is that the molecular and morphological analyses differ in fundamental topology (*7*, *10*, *11*, *22*, *32*, *33*). For example, in morphological analyses, the Iguania are frequently placed in a basal position and Gekkonomorpha nested higher in the tree, whereas phylogenomic trees all agree that Gekkonomorpha is the basal clade, and Iguania are derived. This affects clade definitions and makes placement of fossil taxa difficult. For example, the Anguimorpha from morphological analyses generally includes skinks and amphisbaenians, whereas Scincomorpha and Amphisbaenia are more basally placed in the molecular tree. The snakes are placed basally within the Toxicofera in the phylogenomic tree (*34*) but are more crownward in the combined molecular and morphological tree (*32*).

**Fig. 8.**
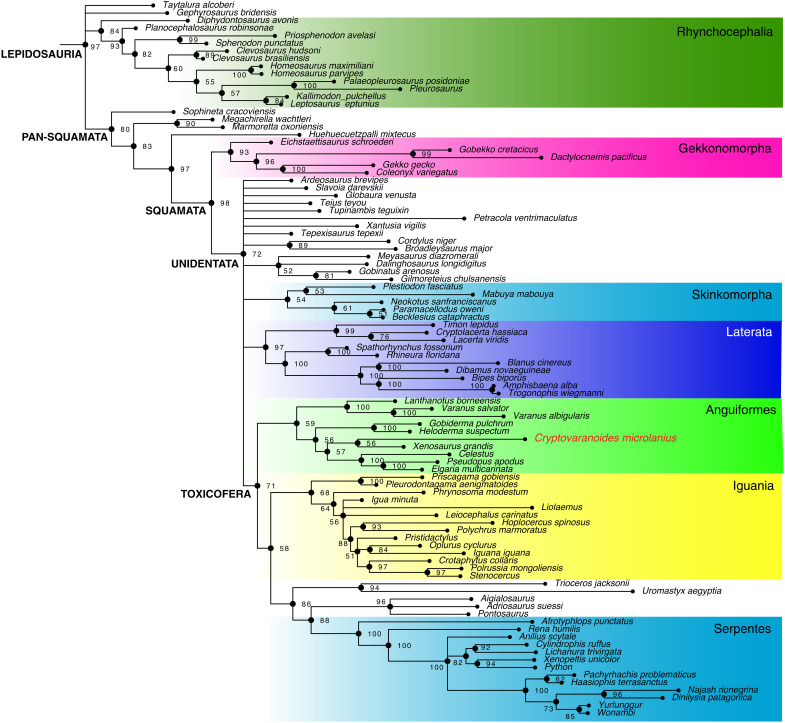
Phylogenetic analysis of Squamata and phylogenetic placement of *Cryptovaranoides*. Maximum parsimony tree from a Bayesian-inference analysis of combined molecular and morphological data based on the data matrix of (*32*). Numbers at nodes are posterior probabilities. Full details in the Supplementary Materials.

Therefore, we follow current practice (*10*, *11*, *32*) in performing phylogenetic analyses with a combined molecular-morphological data matrix. As in previous analyses [e.g., (*34*, *35*)], the tree (Fig. 8) shows the maximum clade credibility (MCC) tree from molecular data, with Gekkonomorpha basal and the Toxicofera (Anguiformes, Iguania, and Serpentes) a major clade. Our analysis identifies Rhynchocephalia as a clade and positions some basal taxa as either Pan-squamata (*Sophineta*, *Megachirella*, and *Marmoretta*) and others (*Taytalura* and *Gephyrosauru*s) as Lepidosauria. However, our phylogeny of Squamata (Fig. 8) conforms with recent analyses (*10*, *11*, *32*, *34*, *35*), in discovering Gekkonomorpha basal, and the major clades Unidentata, Toxicofera, Skinkomorpha, Laterata, Anguiformes, Iguania, and Serpentes. Unexpectedly, we failed to retrieve complete resolution of clades between Unidentata and Toxicofera, and this relates to uncertainty in the placement of several Jurassic and Cretaceous fossil lizards (e.g., *Ardeosaurus*, *Globaura*, and *Tepexisaurus*).

The new Triassic lizard, *C. microlanius*, is nested deep in the phylogeny of crown Squamata as successively Squamata, Unidentata, Toxicofera, Anguiformes, Neoanguimorpha, and Anguioidea (*34*), which suggests that it is related to modern xenosaurids, anguids, and helodermatids (Fig. 8). Because this is a fossil taxon, we lack molecular data; however, morphology-only phylogenetic analyses confirm that *C. microlanius* is a crown squamate and an anguimorph (further details in the Supplementary Materials).

### Character analysis

We step through numerous characters that confirm that *Cryptovaranoides* belongs to each of these clades (further details in the Supplementary Materials). *Cryptovaranoides* shares 10 synapomorphies with the clade Pan-squamata: Angular does not extend posteriorly to reach articular condyle; coronoid eminence formed by coronoid bone only, not in the combined process of the dentary and prominent dorsal expansion of the surangular; quadratojugal not present as a separate element; short overlap in quadrate-pterygoid contact; scapulocoracoid emargination/fenestration present; jugal posterior process absent; coronoid postero-medial process present; jugal closely approaches level of prefrontal below orbit; jugal entirely exposed above labial margin of maxilla; coronoid reaches lateral side of surangular.

Unequivocal squamate apomorphies in *Cryptovaranoides* are fused premaxillae and premaxillary unfused median tooth. A further 13 squamate synapomorphies are as follows: cephalic head of mobile quadrate with laterally positioned notch, peg-in-notch articulation with rod-shaped squamosal, vomer and maxilla meet at anterior margin of fenestra exochoanalis, prominent choanal fossa on anterior margin of ventral surface of palatine (this is particularly noticeable in larger unregistered bones in the collection where the posterior margin of the fossa extends posteriorly to the anterior edge of the suborbital fenestra/foramen), subdivision of embryonic metotic fissure by the crista tuberalis into vagus (jugular) foramen and recessus scala tympani, enclosed vidian canal exiting anteriorly at base of each basipterygoid process, no quadrate/quadratojugal foramen, medially positioned posterior myohyoidal foramen on mandible, fusion of exoccipitals and opisthotics forming an otoccipital, coronoid anteromedial process fits into sulcus beneath tooth-bearing border of dentary, frontal underlaps (or barely overlaps) parietal laterally on frontoparietal suture, palatine extends posteriorly so pterygoid enters sub-orbital fenestra, and trunk vertebrae lack intercentra.

*Cryptovaranoides* shows five synapomorphies of Unidentata: frontoparietal suture moderately interdigitated, rugose ornamentation over dorsum, jugal lies ventral to lacrimal, posterodorsal trending ridge delineates anterior limits of naso-lacrimal fossa, and septomaxilla probably contacts dorsal surface of palatal shelf of maxilla (septomaxillary facet on maxilla). *Cryptovaranoides* shares four synapomorphies with Anguimorpha: frontal underlaps parietal laterally on frontoparietal suture, short overlap in quadrate-pterygoid contact, long ventral longitudinal ridges converging toward midline of vomer, and lacrimal probably arches dorsally over lacrimal duct and floors lacrimal duct with medial process posteriorly.

## DISCUSSION

**Fig. 9.**
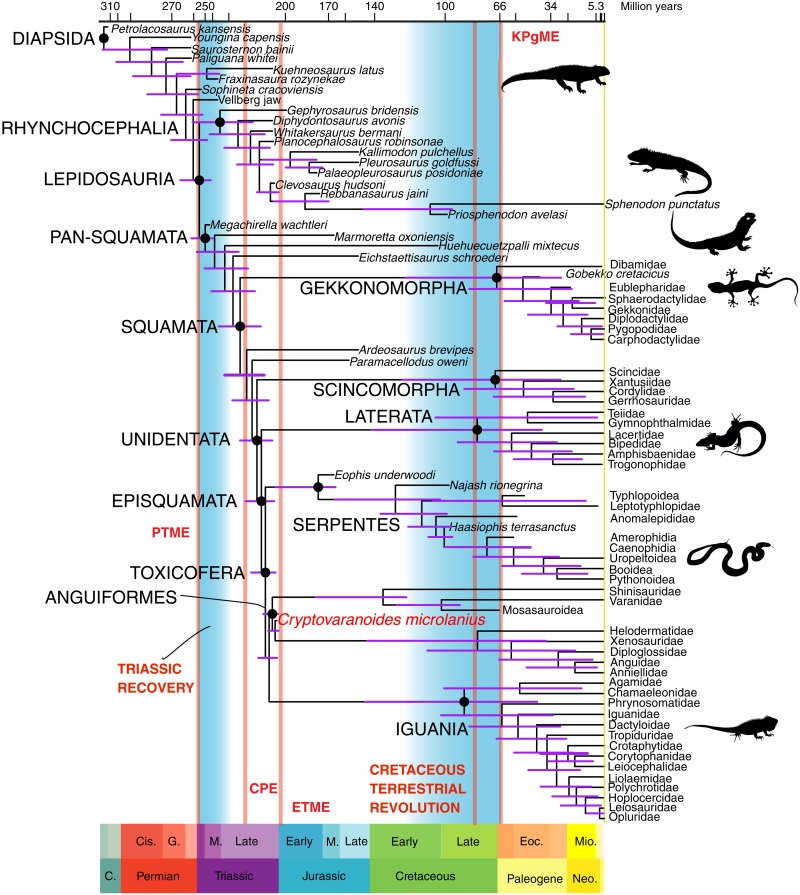
Phylogeny of Lepidosauria showing dating estimates for key clades. The tree shows all major squamate groups, constrained on a recent molecular phylogeny (*34*), and displays the effect of *Cryptovaranoides* on dating major times of divergence. PTME, Permo-Triassic mass extinction, 252 Ma; CPE, Carnian Pluvial Episode, 232 Ma; ETME, end-Triassic mass extinction, 201 Ma; KPgME, Cretaceous-Paleogene mass extinction, 66 Ma.

The new fossil has three profound effects on our understanding of squamate evolution. First, it alters our understanding of the origins of numerous included clades, such as Unidentata, Episquamata, Toxicofera, and Anguiformes. Dating all these clade origins is crucial for setting the time scale of modern squamate biodiversity (*6*–*8*, *10*, *22*, *34*, *36*). Identifying *Cryptovaranoides* as an anguimorph squamate pushes many of the deep divergences within Squamata back from Middle-Late Jurassic to Late Triassic (Fig. 9), a span of 30 to 50 Ma. This will have substantial effects on calculations of rates of evolution of traits in future macroevolutionary work.

Second, the re-dating of pan-squamate origins not only confirms a Triassic origin, as had been predicted from the Triassic origin of their sister clade, Rhynchocephalia, but also shows that this predicted Late Triassic origin comprised more than just one or more basal squamate forms, but that many of the modern clades track back to that time, suggesting a broad-scale diversification of major lizard clades, minimally Gekkonomorpha and Unidentata.

Third, the new fossil, placed in the phylogenetic tree (Fig. 8), indicates a possible trigger for squamate diversification at this point. Age-dating of deep clades shows that many originated in the Late Triassic, well before the end-Triassic mass extinction (201 Ma), but well after the end-Permian mass extinction (252 Ma). They fall close to the Carnian Pluvial Episode (CPE), 232 Ma, already identified as a major stimulus for the origins of new clades of terrestrial plants, insects, and tetrapods (*37*). We propose here that the CPE might also have triggered the rapid expansion of Squamata and multiple major included squamatan clades. The rapid divergence of Lepidosauria and Pan-squamata in the Early-Middle Triassic, as indicated by *Megachirella* and the Vellberg jaw, matches that of dinosaurs, pterosaurs, and synapsids (*38*) in the recovery time following the end-Permian mass extinction (Fig. 9). At local scale, our dating of *Cryptovaranoides* at the time of the end-Triassic mass extinction suggests that it may have been part of the radiation of squamates that eventually surpassed rhynchocephalians, which are diverse in the Rhaetian of the United Kingdom (*39*, *40*).

Through the Jurassic and Cretaceous, squamates diversified further, as represented by incomplete remains of fossil lizards and snakes from the latest Early Jurassic and Middle Jurassic, and more complete fossils in the Late Jurassic and Early Cretaceous. To what extent the patchy fossil record of squamates in the Jurassic reflects genuine rarity or poor sampling (*41*) or a combination is hard to say. Nonetheless, the diversity of rhynchocephalians apparently declined in the Early Cretaceous in Laurasia (*41*). After that, rhynchocephalians remained at low diversity through the present single species, *Sphenodon punctatus*, whereas squamates further diversified through the late Early and Late Cretaceous, from 125 to 66 Ma. This was the time of the Cretaceous Terrestrial Revolution, and important time of diversification of angiosperms, insects, and spiders that might have stimulated the diversification of lizards and snakes (*35*, *42*–*46*). The mass extinction at the end of the Cretaceous during which several lineages of squamates died out, as well as the marine mosasaurs, also triggered further diversifications among squamates in the subsequent Paleogene (*41*, *42*). A key question for phylogenetic-macroevolutionary studies is to determine the extent to which these different events triggered different phases of squamate diversification. Furthermore, dating the origins of major clades correctly provides data on which to test whether the macroevolution of these reptiles was driven primarily by changing physical environments (changing temperatures, moving continents, and aftermath of crises), by new ecological opportunities (e.g., burgeoning insects in the mid-Cretaceous associated with diversification of flowering plants), or by innovation (new adaptations).

## MATERIALS AND METHODS

### Specimens

The provenances and ages of the specimens are well documented. The type specimen (Figs. 1, 5, A to I, 6, A to D, G, and H, and 7, A, C to E, K, L, and N) was collected in 1953 by P. L. Robinson of University College, London, from a fissure filling at Cromhall (formerly Slickstones) Quarry, near Bristol. Details of the fissure are given in (*47*, *48*) and in the unpublished notebooks of P. Robinson held in the NHMUK. All aspects of the rock matrix including specimens of the rhynchocephalian *Clevosaurus* (a large T-shaped interclavicle and a possible pubis) confirm that it is Late Triassic in age. The fissure infills at Cromhall have been dated variously from late Carnian to Rhaetian, but biostratigraphically useful fossils (palynomorphs from nearby fissure localities and conchostracans from Cromhall and other fissure sites) and fissure filling correlated with the Rhaetian marine transgression (*49*) confirm a Rhaetian (205 to 201 Ma) age. The collections of fossils incorporating *C. microlanius* and *Clevosaurus hudsoni* that included the conchostracan *Euestheria brodieana*, a crustacean only found in the United Kingdom in the Cotham Member, Lilstock Formation, Late Rhaetian (*50*), affirm the dating as Late Rhaetian [201.6 to 201.8 Ma; (*15*)]. The specimens and CT scans are the property of the NHMUK and can be accessed with permission from the Department of Earth Sciences, The Natural History Museum, Cromwell Road, London SW7 5BD, UK.

### Imaging

Photographs were taken with the Bristol University Leica M205C optical microscope and Leica DFC425 C digital microscope camera. We used the multifocus option and Leica application suite (LAS) software to produce the best possible photo-stacked images of isolated bones. Additional photographs of isolated bones were taken by D.I.W. using the Canon EOS photo-stacking and Helicon focus processing software at the Angela Marmont Centre of the NHMUK. All images were handled using Adobe Photoshop 6.

Backscattered electron scanning electron microscopy (SEM) images were obtained at the EM Labs, School of Earth Sciences at Bristol University using a Hitachi 5500-N SEM operating under low vacuum (30 to 50 Pa), with an accelerating voltage of 15 kV on uncoated samples.

### 3D imaging and processing

An initial CT scan was taken at the NHMUK with a resolution of 43 μm, and although many features could be discerned following segmentation (Avizo 8.0), it was not possible to resolve some key issues such as the nature of tooth implantation as the resolution was too low. Therefore, two further scans, at a higher resolution, were conducted using the X-Tek XT H 225 ST microfocus CT scanner (Nikon Metrology), with microfocus 225-kV source, which is fitted with a tungsten reflection target, and PerkinElmer detector (XRD 1620 detector), belonging to the Palaeobiology Research Group, and located in the Life Science Building, University of Bristol. The scan settings for the left mandible and associated anterior portion of the articulated skeleton of NHMUK PV R36822 used 222 kVp, 153 μA, 500-ms exposure, and 3141 projections, which were acquired by a full rotation of 360° using four frames per projection. A 17.5-μm reconstructed voxel resolution resulted from a source to object distance of 103 mm and a source to detector distance of 1176 mm. There was no filtration of these scans other than the beryllium window. The scan settings for the associated skull of NHMUK PV R36822 used 200 kVp, 200 μA, 708-ms exposure, and 6284 projections, which were acquired by a full rotation of 360° using one frame per projection. A 43.2-μm reconstructed voxel resolution resulted from a source to object distance of 255 mm and a source to detector distance of 1180 mm. For these scans, we used 0.5-mm Cu filtration and a beryllium window. The scan settings for the braincase NHMUK PV R37377 used 130 kVP, 48 μA, 1000-ms exposure, and 3141 projections, which were acquired by a full rotation of 360° using one frame per projection. A 6.3-μm reconstructed voxel resolution resulted from a source to object distance of 37 mm and a source to detector distance of 1175 mm. There was no filtration of these scans other than the beryllium window. In all cases, the specimens were mounted with phenolic foam to prevent movement during rotation.

Scans were reconstructed using CT Pro 3D (Nikon Metrology). The two batches of CT scans were then processed using Avizo 8.0 (FEI Visualization Sciences Group) 3D-visualization software. Automated segmentation proved impossible as the density of the limestone and fossil bone was very similar, resulting in poorly contrasted x-ray attenuation properties on the CT scans. Therefore, segmentation was performed manually. Bones were individually segmented from the matrix and then identified and assigned labels within Avizo, before a 3D surface model was generated. In general, although the contrast was low, it was possible to confidently visualize all fossil bone within the scanned regions of NHMUK PV R36822. The only exception was the two lacrimal bones, which could only be partially discerned in the CT scans but were both clearly visible under the microscope.

### Phylogenetic analyses

We carried out five phylogenetic analyses: (i) a combined molecular-morphological analysis using Bayesian techniques on the Martínez *et al.* (*32*) data matrix (Fig. 8) and (ii to v) morphology-only analyses using parsimony and Bayesian techniques on the data matrices of Simões *et al.* (*10*) and Griffiths *et al.* (*11*), respectively.

For the Bayesian analyses, we used MrBayes v. 3.3.7a (*51*) to infer MCC trees, using the standard Mk model, with specification of gamma distribution priors for site rate variation. The data matrix of Martinez *et al.* (*32*) includes 145 taxa and 349 morphological characters; we deleted *Vellbergia*, identified as a rogue taxon (*32*), and added *Cryptovaranoides*, coded as below (in the Supplementary Materials). The molecular data comprise in total 14 nuclear and 2 mitochondrial loci (11,532 base pairs), sampled from 47 extant taxa. Fossil taxa are coded as unknown for the molecular partition of the data. We performed the analysis with four runs of six chains each, run for 40 million generations, and sampling parameters every 1000 generations. The first 50% of sampled trees were discarded as burn-in. Convergence was assessed on the basis of effective sample size (ESS) values >200, and Potential Scale Reduction Factor, (PRSF+) = 1.000. Results from the Bayesian analysis were summarized using the MCC tree, and strict and 50% majority consensus trees, retaining clades that have posterior probability values of ≥50%.

We used a number of current data matrices including Triassic diapsids and a broad sample of modern lizards; we focused on those of Simões *et al.* (*10*), Griffiths *et al.* (*11*), and Martinez *et al.* (*32*). *Vellbergia bartholomai* [data from (*33*)] and *Taytalura alcoberi* (*32*) were added to the Simões *et al.* (*10*) matrix. The recent morphological data matrices (*10*, *11*) are connected, in that Griffiths *et al.* (*11*) revised the Simões *et al.* (*10*) matrix, adding and modifying characters as well as rescoring them for many taxa. Griffiths *et al.* (*11*) also removed some taxa included by Simões *et al.* (*10*) and added others. Martinez *et al.* (*32*) made some small modification to the data matrix of (*10*). We have used all these matrices with no modification to the character descriptions, and little or no amendments to their scoring (except where stated) but have added *C. microlanius* to all three matrices, two of which (*10*, *11*) share much of their structure. We added one character to the Simões *et al.* (*10*) and Griffiths *et al.* (*11*) matrices, which references the occurrence of a median premaxillary tooth. The advantage of using these three matrices is that they are composed by other authors, and we can therefore assess the most likely phylogenetic relationships of *Cryptovaranoides* without biasing the character or taxon selection. We inserted newly scored characters for *Cryptovaranoides* and rescored characters for certain fossil taxa, primarily *Megachirella* and *Marmoretta* in (*10*) (appendix S3). We scored characters 348 and 349 in the Martinez *et al.* (*32*) dataset as 0 and 0, respectively, for *C. microlanius*, but the scoring of all other taxa is the same as for (*32*).

In the Phylogenetic Analysis Using Parsimony (PAUP) analyses, we used the branch and bound method. In TNT (*52*), the most parsimonious trees (MPTs) were analyzed using traditional tree bisection and reconnection (TBR) branch swapping, and the new technology tree fusing, ratchet, and sectorial searching algorithms. This was followed by TBR branch swapping to find all MPTs up to a maximum of 100,000 held. All recovered MPTs were then summarized in a strict consensus tree, and clade robustness was assessed with Bremer decay indices from TBR branch swapping of 8000 trees. We also ran the TNT New Technology search in all cases, deploying the parsimony ratchet, tree-fusing, tree-drifting, and sectorial searches, and then calculating a “nelsen” strict consensus tree, and with calculation of bootstraps and Bremer supports.

### Tree dating analysis

We dated a fixed tree topology using the fossilized birth-death (FBD) method implemented in MrBayes (*51*), following recent recommendations (*53*). The tree topology (Fig. 9) is based on the most recent molecular phylogenetic tree (*34*). We used the MKv model of evolution, with gamma distribution to model among character rate variation. We selected the TK02+ln clock model (TK02, relaxed continuous autocorrelated clock with values sampled from a lognormal distribution), with an exp (*10*) hyperprior on the shape of the lognormal distribution from TK02. We used the “diversity” FBD model, in which the fossil taxa are assumed to be sampled randomly and modern taxa are assumed to be sampled in a way that maximizes diversity, and fossils can be tips or ancestors. Furthermore, we did not sample for ancestors [the NoSA strategy of (*53*)]. We set uniform age ranges for each of the included 78 taxa, whether living or fossil (appendix S6), and fixed the tree age prior equivalent to *Petrolacosaurus* (307 and 312 Ma). The analysis was run for 1 million iterations, achieving convergence (average square difference function, ASDF of 0.0003, PSRF = 1.014, and ESS > 200 for all parameters). Relative burn-in was 0.25, and trees were sampled every 500.

## Supplementary Materials

### This PDF file includes:

Supplementary Text Appendices S1 to S7 Figs. S1 to S6 References
