# Supplementary material for: A Triassic crown squamate
Source: Sci Adv. 2022 Dec 2;8(48):eabq8274. doi: 10.1126/sciadv.abq8274 (PMC10936055; doi:10.1126/sciadv.abq8274)
Supplement: Supplementary file 1 — Supplementary Text Appendices S1 to S7 Figs. S1 to S6 References [file sciadv.abq8274_sm.pdf]

Science Advances
AAAS

Supplementary Materials for

A Triassic crown squamate

David I. Whiteside *et al.*

Corresponding author: Michael J. Benton, mike.benton@bristol.ac.uk

Sci. Adv. **8**, eabq8274 (2022)
DOI: 10.1126/sciadv.abq8274

This PDF file includes:

Supplementary Text
Appendices S1 to S7
Figs. S1 to S6
References

Supplementary text

The fossils of *Cryptovaranoides* The holotype is a small block of red sandy limestone, NHMUK PV R36822 (Fig. 1A) with several cranial and postcranial elements, labeled as '*Clevosaurus* plus one other reptile'. It was clear that at least one of the fossils on the rock, a partial skull, was new, not a clevosaur and of considerable interest. The rock came from the same red conglomeratic limestone matrix that had yielded *C. hudsoni*. The collecting site has been described before (*47, 48, 54)*. The rocks were collected within 2–3 m of the pre-quarrying surface (*48*) in the southerly part of the quarry; the fissure was destroyed in quarrying but exposures at one end remain (although with a different lithology), the site 2 of Fraser (*19*). Site 1 of Fraser (*19, 55)* may have also been linked to the now destroyed fissure. Further details on the south-west U.K. fissure sites, the terrestrial fauna, and the fissure fills, and their formation, have been presented (*40, 56*).

The fossil is separated into two areas of the rock specimen. The left lower jaw as well as a partial skull consisting of both maxillae and associated bones, some in near life positions, has been separated by a centimeter from the articulated partial skeleton. The left side of the skull and lower jaw has twisted away from the right side (which lacks the mandible) so that the latter lies in a different plane and at right angles. In near life positions next to the left maxilla are a prefrontal, jugal, and lacrimal. The partial skeleton consists of the right mandible with associated quadrate, a right postorbital, right jugal, (a possible) otoccipital, basicranial bones and articulated cervical and anterior dorsal vertebrae, as well as all the pectoral girdle and many of the forelimb elements. Near these articulated elements is another group of dorsal vertebrae and near them is an additional basioccipital (Figs. 1A, D, 7B) from a larger individual of *Cryptovaranoides*. We are sure that the separate skull belongs to the same individual, as no bones are replicated in the two areas and where there are separated left and right elements, such as in the lower jaw and the jugals, the bones match in all features. There are at least three other bones positioned in the middle of the rock specimen (Fig. 1A) which consist of an interclavicle, a possible pubis, and another unidentified bone. However, we are unsure whether these elements are from *Cryptovaranoides* and, as a result, they are not considered here.

The articulated bones on NHMUK PV R36822 have enabled us to recognize isolated bones from the Robinson collection, which has greatly aided our understanding of this new squamate. These isolated bones were prepared by Frances Musset and Pamela Robinson. They provide first-hand observations of facets, bone morphology and dentition. They also have a range of sizes and morphologies which has allowed us to describe the variability of key elements from the new species. The holotype with an emphasis on additional isolated bones enable us to compile the reconstruction of Figure S1.

Dating and geology. The dating and paleoenvironment of the Cromhall fissure deposits has proved controversial. Earlier authors (*47,55*) regarded the red deposits as Norian, but others (*40, 56*) argue they are Rhaetian and from low-lying limestone islands formed around the time of the Rhaetian transgression across Europe. Our specimen NHMUK PV R36822 was found in a Late Triassic fissure deposit that also contained specimens of the spinicaudatan conchostracan *Euestheria brodieana*, collected by Pamela Robinson (*50*), a biostratigraphic indicator from the Cotham Member, Lilstock Formation, Late Rhaetian (*50*) that provides definitive evidence of the age of *Cryptovaranoides*. The Cotham Member dates from towards the end of the Rhaetian stage, latest Triassic, whose total duration is 4.1 Myr, and within the age range 205.5–201.4 Ma (*15*), and these authors indicate an age range for the Cotham Member of 201.6–201.8 Ma. However, also see (*40,49,57,58*) for the most recent debate on the age of these fissures.

The clade name Squamata

The name Squamata when applied to living forms *only* is unequivocal – it comprises all lizards, amphisbaenians and snakes, and was equal to the sum of clade Iguania plus clade Scleroglossa, under the original morphology-based cladistic analyses (*30,36,59*). Phylogenomic analyses (*8,10,32,34,44,45*) showed that the included clades were more likely Gekkonomorpha + Unidentata (= 'Autarchoglossa" + Iguania), but the arrangement of those clades of living forms does not affect the nature of crown Squamata.

However, with the addition of fossils, the meaning of the term 'Squamata' became equivocal – many fossils belong to the living clades, and so did not alter its meaning, but certain lizard-like fossils from the Triassic and Jurassic cannot be assigned to the modern clades, and so belong outside the strict definition of the term.

Systematists distinguish three sets of taxa in a case such as this: the **crown** clade is the clade comprising all living forms plus their ancestors down to their last common ancestor. Close relatives outside the crown clade belong to the **stem**, and stem + crown = the **total** group. This matters for dating phylogenomic trees: a stem lizard from the Jurassic or Triassic lies outside the crown and so cannot date the crown. Paleontologists have claimed numerous 'lizard' fossils from the Permian and Triassic, but none of these hitherto has been a convincing crown squamate. It is important to resolve the confusion over usage of the term 'squamate', especially here, as we provide evidence that *Cryptovaranoides* is embedded deep within the crown-group Squamata, and we clarify the terms in Figure S2.

The term Squamata was redefined as the term for the crown (*30,59*). Estes et al. (ref. *59*, p. 186) defined the Squamata as 'The most recent common ancestor of Iguania and Scleroglossa (i.e., the crown clade). Others e.g., Conrad (*36*) considered the clade Squamata to be sister to the Rhynchocephalia. He noted (*36*, p.73): 'common usage of the name (Squamata) incorporates all non-rhynchocephalian members of the crown-node Lepidosauria.' Here, we follow crown-group usage of Squamata.

The oldest crown squamates reported previously come from the Middle Jurassic (Bathonian, 168.3–166.1 Ma) of England (*5*), whereas the oldest stem squamates come from the Triassic *(10),* so there is a considerable difference in content and date of origin of the total and crown-group clades.

Therefore, there is a clear need to define terms and we use the most recent (Fig. S2) (*12,13, 60,61*). The term for the total group is Pan-squamata Gauthier and de Queiroz (*12*), defined as 'The total clade of the crown clade *Squamata*.' It is further defined (*12*) as '*Squamata* and its stem group—that is, *Squamata* and all extinct species that are more closely related to that crown clade than they are to *Sphenodon punctatus*.' 'The crown clade Squamata is defined (*13*) as: 'The largest crown clade containing *Lacerta agilis* Linnaeus 1758 but not *Sphenodon* (originally *Hatteria*) *punctatus* (Gray, 1842). This is a maximum-crown-clade definition.'

This then clarifies the positions of fossils relative to the modern taxa. Because *Cryptovaranoides microlanius* is a crown (not stem) squamate as well as a pan-squamate, it matters to be precise so that fossil dates can be used accurately in phylogenomic analysis. Its date marks the oldest fossil confidently assigned to Squamata, the clade of living forms; older putative non-squamate pan-squamates such as *Megachirella* cannot help in dating the origin of modern squamates such as that given by (*7,8*).

Phylogenetic analysis

Morphology-only analyses. In accord with (*12,13*), we compare *Cryptovaranoides microlanius* characters to the morphological synapomorphies of the Squamata of (*22*). We present Bayesian, combined molecular-morphological data analyses (see main text) and morphology-only analyses.

In the morphology-only analyses, we used the data matrix of *(11)* to establish the relationships within Pan-Lepidosauria (*60*) as this includes the most significant Triassic fossil taxa as well as recent squamates. Output statistics for the analyses are listed (Appendix S1). We had initially used the dataset of (*7*) for a guide but this included a metataxon (rather than individual genera) for Squamata and was targeted by those authors at the Rhynchocephalia and Pan-lepidosauria, It also had only 100 characters and lacked key taxa such as *Hueheuecuetzpalli*. Therefore, we focused on the much larger morphological and recent datasets *(10,11)* as they include 247–277 additional characters and about 100 more taxonomic units.

The Simões *(10)* data matrix (Appendices S2, 3) including revised character scorings for *Megachirella* and *Marmoretta* (see Appendix 3 for re-scorings). We also added character 348 which references the presence or absence of a median premaxillary tooth; this is a modified version of character 413 of (*22)* and originally in *(62)*. We explored these data using PAUP, running a standard heuristic parsimony search, and TNT, also running a standard heuristic parsimony search with TBR branch swapping, and a New Technology search. In each case, we added *Vellbergia bartholomaei* (*63*), *Taytalura alcoberi (32)* and *Cryptovaranoides microlanius*; we excluded taxa 1–12 and 14–52, retaining *Youngina*, *Palaeagama*, *Kuehneosaurus*, and *Icarosaurus* as outgroups to Lepidosauria. This corresponds to removal of 51 taxa, leaving 81 taxa to be analyzed. One consequence was that many characters became uninformative, and we removed those, amounting to 82 of the 348 characters – these 82 were either invariant or showed the derived state in only one taxon, and this could either apply in any case across the whole matrix, or this might be a consequence of removal of most non-lepidosaurians.

The statistics for the runs of the Simões *(10)* data set are very similar (Appendix S1). We show the outputs from the TNT parsimony and NT analyses (Fig. 4A, B) and the MrBayes Bayesian analysis (Fig. S4C). *Cryptovaranoides* is placed as a basal member of Anguimorpha, within the Unidentata, in the strict consensus trees of both the PAUP and TNT parsimony (Fig. S4A) and TNT NT runs (Fig. S4B). In both cases, strict and majority rule trees are the same, probably because the TNT cladistic analyses are a consensus trees of small numbers of source trees.

The MrBayes runs gave a reasonably well resolved MCC (Fig. S4C), and at least confirms the phylogenetic position of *Cryptovaranoides* as part of the Anguimorpha clade. In the Bayesian analysis, the Rhynchocephalia are fully resolved, as in the parsimony trees, but Pan-squamata and Squamata are less resolved. The major clades Gekkonomorpha and Unidentata are distinguished, with outgroups following in sequence: *Eichstaettisaurus schroederi*; *Huehuecuetzpalli mixtecus*; *Marmoretta oxoniensis* and *Megachirella wachtleri.* This would then form the Pan-Squamata with *Megachirella* as the most basal member. Sister to the Pan-squamata is the Rhynchocephalia with *Gephyrosaurus* as the basal member and sister to the Lepidosauria is a polytomy comprised of early lepidosauromorphs (*Paliguana whitei+ Sophineta cracoviensis+ Vellbergia bartholomaei+Taytalura alcoberi*) and a clade of kuehneosaurs+ *Pamelina*+ *Palaeagama*.

We also used the Griffiths et al. *(11)* matrix which substantially revises the Simões et al. *(10)* data, adding 32 new characters, modifies an additional 8 characters, removes some taxa and includes new taxa as well as rescoring many taxa in common to both matrices. We again added a character (Ch. 378) on the presence or absence of the median premaxillary tooth. We ran parsimony analyses in PAUP and TNT and a run using MrBayes. A parsimony analysis is not

reported by *(11*) but we ran MrBayes and the other the programs with all taxa in the manner of those authors. The TNT strict consensus trees (Fig. S5A, C) are poorly resolved and although there are subclades that include some anguimorphs, others are placed in a large polytomy which has subclades of e.g., tanystropheiid archosauromorphs, drepanosaurs, rhynchocephalians, early turtles and a number of taxa of modern and extinct lizards including *Cryptovaranoides.* The majority rule parsimony tree (Fig. S5B) has much better resolution with a Pan-Squamata clade and its sister clade, the Rhynchocephalia, forming the Lepidosauria. *Marmoretta oxoniensis* and *Fraxinisaura rozyneka* are in the subclade sister to the Lepidosauria forming the Pan-lepidosauria. *Paliguana whitei* is positioned in a subclade with archosauromorphs (e.g., rhynchosaurs, *Prolacerta* and trilophosaurs) as sister to the Pan-lepidosauria. Within the Pan-Squamata *Sophineta cracoviensis* is the basal stem-squamate followed crownwards by *Megachirella wachtleri; Huehuecuetzpalli mixtecus* is sister to the Squamata and that clade is separated into the Gekkonomorpha and Unidentata. However, probably anomalously, *Palaeagama vielhaueri* is partnered with *Huehuecuetzpalli mixtecus. Cryptovaranoides* is positioned as a basal member of the clade Anguimorpha

The TNT new technology majority rule tree (Fig. S5D) finds many of the same clades, including Squamata (inclusive of *Eichstaettisaurus schroederi* as a Gekkonomorph) followed by *Huehuecuetzpalli mixtecus*, *Megachirella wachtleri*, and *Sophineta cracoviensis* as successive outgroups. *Palaeaagama, Paliguana, Marmoretta* and *Fraxinisaura* are all in a large polytomy with archosauromorphs and turtles outside the Lepidosauria. Within Squamata, Gekkonomorpha and Unidentata are distinguished, and *Cryptovarnoides* this time is placed as a basal member of Iguania.

The MrBayes MCC (Fig. S5E) identifies some unusual groupings among the non-lepidosaurs, for example pairing basal archosauromorphs with marine reptiles, but it identifies the major clades Rhynchocephalia, Pan-squamata and Squamata, and places *Cryptovaranoides* as an unresolved member of the Unidentata clade. In all cases, *Cryptovaranoides* is unequivocally a member of Pan-squamata, Squamata, and Unidentata, and sometimes of Anguimorpha.

General outlines of phylogeny. Some of our cladistic analyses did not include non-lepidosauromorphs, but the Griffiths et al. *(11)* trees cover all diapsids (Fig. S5) as do the combined molecular-morphological data matrix Martinez et al. *(32)* trees (Fig. S3). The strict consensus tree based on based on the Martinez et al. (*32*) dataset form a large, essentially unresolved, polytomy (Fig. S3A) but the majority rule tree (Fig. S3B) depicts three clades: the pan Squamata, a rhynchocephalian clade (albeit without *Gephyrosaurus*) and a clade composed of Archosauromrphs, marine reptiles and other groups such as the rhynchosaurs. The maximum clade credibility tree (Fig. S3C) also has many taxa within a polytomy but resolves lepidosaurian clades much more clearly with Rhynchocephalia and Pan Squamata as distinct sisters; *Cryptovaranoides* is well positioned in the anguimorphs amongst the Squamata. In the trees based on the Griffiths et al. *(11)* dataset, an unusual clade comprising Archosauromorpha plus marine reptiles (Ichthyosauria, Sauropterygia) is identified, and below it are clades of turtles, (coelurosauravids + kuehneosaurids), and some early diapsids.

All trees identify Pan-lepidosauria and Lepidosauria, and the two main subclades, Rhynchocephalia and Pan-squamata. Within Rhynchocephalia, the most frequently positioned basal rhynchocephalian is *Gephyrosaurus* but often partnered with *Diphydontosaurus* in the *(11)* data. However, *Sophineta* is the basal rhynchocephalian in the MCC of the *(11)* data. Depending on the matrix used, *Vellbergia bartholomaei, Paliguana whitei*, *Taytalura alcoberi* and, sometimes, *Sophineta cracoviensis*, are placed outside the Lepidosauria (*61*) in the Pan-

Lepidosauria (*60*) in the modified matrices of (*10* and *11*), particularly the Simões et al. matrix *(10).* In the modified matrix of *(11)* using parsimony and Bayesian analyses, *Paliguana* is puzzlingly positioned in a clade with archosauromorphs such as *Proterosuchus*, rhynchosaurs and trilophosaurs. In the TNT and PAUP strict consensus and Majority rule (Fig. S4) trees based on the modified matrix of *(10)*, the kuehneosaurids are in a clade with the pan-lepidosaurs *Pamelina* and *Palaeagama*; likewise, the *(11)* matrix parsimony and TNT NT results in a similar subclade grouping but without *Palaeagama.* The Bayesian MCC trees of both matrices position those taxa similarly to the respective TNT trees.

All analyses identify Pan-squamata and Squamata, with outgroups of Squamata comprising *Megachirella*, *Marmoretta*, *Huehuecuetzpalli*, and, frequently, *Eichstaettosaurus*, all examples of Mesozoic lepidosaurs. Within Squamata, the two clades Gekkonomorpha and Unidentata are commonly identified, in line with current molecular phylogenetic analyses (*8,32,34,35,44,45*), and in opposition to classic, morphology-based cladograms (e.g., *9,17,22*) that commonly identified Iguania as sister to the other squamates, termed the Scleroglossa. The Autarchoglossa was another traditional major squamate clade, not identified in these analyses or in recent molecular analyses, comprising skinks, anguimorphs, snakes and relatives, essentially the Unidentata minus Iguania.

Our evidence from all key phylogenetic analyses is that *Cryptovaranoides microlanius* is the earliest undoubted squamate. In the resolved parsimony and Bayesian MCC trees using the detailed modified character matrices and multiple taxa of (*10* and *11*). *Cryptovaranoides* is placed within the Squamata (= crown Squamata) always crownward of Gekkonomorpha. It is always crownward of *Huehuecuetzpalli mixtecus* which is generally regarded as the sister taxon to the Squamata *(*e.g.,*11*,*22)* although Pyron *(8)* and Evans *(17)* have placed that taxon within the Squamata. Simões et al. *(10)* placed *Huehuecuetzpalli* as a sister taxon to all crown squamates in their extended data figures 3–6 and Gauthier and De Queiroz (*12*) listed 19 apomorphies shared between the 'stem-squamate' *Huehuecuetzpalli* and squamates. In contrast, *Cryptovaranoides* is positioned in the anguimorph toxicoferans (Fig. 3) using the matrix of *(32)*, the anguimorph unidentatans using the modified *(10)* matrix and basally in the Iguania using the *(11)* matrix. *Cryptovaranoides* is the basal anguimorph in all the parsimony analyses using PAUP and TNT with the Simões matrix *(10)* and in the TNT parsimony processing of *(11)* data, but as the basal iguanian in the NT majority rule tree using the *(11)* matrix. In the Bayesian MCC trees *Cryptovaranoides* is an anguimorph using the data of *(10)* and *(32)* but is in a polytomy within Unidentata using the *(11)* matrix.

The remaining squamates such as the skinks, teiids, cordyliformes and lacertiids vary in position according to the matrix used but are grouped together in a clade more basal than the iguanians and anguimorphs in the strict consensus trees of *(10)*. In the combined molecular and morphological analysis using the matrix and procedures of (*32*), *(*Fig. 8) some of these clades are unresolved within the Unidentata. Unlike (*34*, fig. 1), the teiids are not placed in the Laterata and the cordyliformes and xantusiids not positioned as sister to the skinks. However, in common with (*34*) those groups are placed separately from the Toxicofera. In our Fig. 8, the acrodont agamids and chameleons are placed as sister to the Iguania rather than within that clade of (*34*) but as in (*34)* do all lie within the Toxicofera. *Ardeosaurus brevipes* or *Tepexisaurus tepexii* of extinct lizards and *Xantusia vigilis* of extant species are the most basal of the Unidentata (=Autarchoglossa+Iguania) in all trees (except the Bayesian maximum clade credibility) based on the matrix of *(10*). *Tepexisaurus tepexii* is similarly placed using the matrix of *(11)*). However, *Ardeosaurus* is, also positioned high in the crown in the *(11)* data TNT NT and MCC trees and

Palaeagama is placed, seemingly anomalously, in a subclade with *Huehuecuetzpalli* in the TNT parsimony tree using that data (but much more basal in the TNT NT majority rule tree).

The phylogeny (Fig. 4A, B), based on morphology only, lends some support to a Pythonomorpha grouping (*64*) that includes the mosasaurs, *Dibamus* and snakes as well as the amphisbaenians. The parsimony analyses differ from (*6*) where the Dibamidae is found to be the sister to the Gekkonomorpha; here, based on morphology only, it is sister to the amphisbaenians. In contrast, in the combined morphology and morphological analysis (Fig. S8) *Dibamus* is placed in a clade with the Amphisbaenians only. This contrasts with the combined molecular and morphological analysis of (*34*) who placed the Dibamidae as sister to the geckoes but with poor support. They found, however, no significant support for a placing of *Dibamus* with fossorial forms.

Character analysis

We step through the character-based evidence that *Cryptovaranoides* belongs to a series of clades, through Pansquamata, Squamata, Unidentata, Toxicofera, and Anguiformes. The summary of these apomorphies is given in Appendix S7.

Cryptovaranoides microlanius shares ten synapomorphies with the clade Pan-squamata = Squamata+*Huehuecuetzpalli mixtectus* (Appendix S7B , character numbers 22-31): angular does not extend posteriorly to reach articular condyle (Fig. S1B, C, synapomorphy of *Huehuecuetzpalli* according to *12,22*); coronoid eminence formed by coronoid bone only, not in the combined process of the dentary and prominent dorsal expansion of the surangular; quadratojugal not present as a separate element; short overlap in quadrate-pterygoid contact; scapulocoracoid emargination/fenestration present; jugal posterior process absent; coronoid postero-medial process present; jugal closely approaches level of prefrontal below orbit; jugal entirely exposed above labial margin of maxilla; coronoid reaches lateral side of surangular.

Unequivocal squamate apomorphies in *Cryptovaranoides* (Appendix S7A, character numbers 5 and 6), are its fused premaxillae (in the adult, Fig. 4B–C) and a premaxillary unfused median tooth *(22*,*62*). A further thirteen squamate synapomorphies (*10,13,22*) are (Appendix S7A, character numbers 7–15, 17, 19–21): cephalic head of mobile quadrate with laterally positioned notch (Figs. 1C, F, 6D–F) for peg-in-notch articulation with rod-shaped squamosal (=two characters); vomer and maxilla meet at anterior margin of fenestra exochoanalis (Fig. 2D); prominent choanal fossa on anterior margin of ventral surface of palatine (Figs. 7D–E, S1E); subdivision of embryonic metotic fissure by the crista tuberalis into vagus (jugular) foramen and recessus scala tympani; enclosed vidian canal exiting anteriorly at base of each basipterygoid process (Fig. 5E-F); no quadrate/quadratojugal foramen (Fig. 6D–F); medially positioned posterior myohyoidal foramen on mandible; fusion of exoccipitals and opisthotics forming an otoccipital (Fig. 4D–H); coronoid anteromedial process fits into sulcus beneath tooth-bearing border of dentary (Fig. 3E); frontal underlaps (or barely overlaps) parietal laterally on frontoparietal suture; palatine extends posteriorly so pterygoid enters sub orbital fenestra (Fig. 2D, S1D); trunk vertebrae lack intercentra. *Cryptovaranoides* likely shares two further squamate synapomorphies (Appendix S7A, character numbers 16, 18): fronto-parietal joint kinetic and septomaxilla medially positioned (Fig. S1D), forming part of nasal cavity and roofing the vomeronasal organ. Furthermore, the *Cryptovaranoides* feature: coronoid eminence formed by coronoid bone only (Fig. 2B, C, S1B–C, Appendix S7, character 23), is considered a squamatan synapomorphy by (*13*) but as a synapomorphy of the clade Squamata+*Huehuecuetzpalli* by (*12, 22*). In summary, *Cryptovaranoides* shows at least fifteen (sixteen if the coronoid eminence

formed by the coronoid bone only is considered a squamatan synapomorphy) and as many as eighteen synapomorphies of Squamata).

Cryptovaranoides shows five synapomorphies of Unidentata (Appendix S7B, character numbers 32–36): frontoparietal suture moderately interdigitated (Fig. S1D); rugose ornamentation over dorsum (Figs. 7F, S1D); jugal lies ventral to lacrimal (Fig. S1A, F); posterodorsal trending ridge delineates anterior limits of naso-lacrimal fossa (Fig. 3B–C); and septomaxilla probably contacts dorsal surface of palatal shelf of maxilla (septomaxillary facet on maxilla; Fig. 3C).

Cryptovaranoides shares four synapomorphies with Anguimorpha (Appendix S7, character numbers 20, 25, 37, 38) and character 20 is also considered a squamate synapomorphy by (*22*): frontal underlaps parietal laterally on frontoparietal suture (Fig. 7H; also a squamatan apomorphy according to (*22)*); short overlap in quadrate-pterygoid contact (Fig. 2D, S1E; also in lacertoids); long ventral longitudinal ridges converging toward midline of vomer (Fig. 7A); and lacrimal probably arches dorsally over lacrimal duct and floors lacrimal duct with medial process posteriorly (Fig. 1E).

Cryptovaranoides microlanius has the following plesiomorphic or likely plesiomorphic features (Appendix S7A, characters numbered 1-4): (1) the humerus has an entepicondylar foramen (Fig. 1D) not recorded in any extant squamatan; (2) bicapitate ribs are likely on at least one cervical vertebra but that character is described as present in extant *Varanus* by (*29*); (3) the vertebrae are amphicoelous but not notochordal (Fig. 7 N; = amphiplatyan of *11*), however that character is apomorphic to many extant gekkotans which have notochordal amphicoelous vertebrae. Based on bone proportions from CT scanning the anterior ramus of the pterygoid contacts the vomer in the holotype which is usually regarded as (4) a plesiomorphic feature of lepidosaurs but is found, for example, in some extant squamates such as the anguimorph *Shinisaurus* (*36*) and amphisbaenians (e.g., *Rhineura (22*) as well as in a number of extinct polyglyphanodontian genera *(36)*. Thus, *Cryptovaranoides* has just one irrefutable plesiomorph, the presence of an entepicondylar foramen, not found in any species of the current defined Squamata. This trait is shared with *Huehuecuetzpalli* that (*8, 17*) has placed within the Squamata but is generally regarded as a stem squamate (*12*,*22,32*). The other three characters considered as plesiomorphic are found in some living squamate taxa.

Appendix S7 shows the summary of the crown squamate synapomorphy distribution and it is clear that of the 38 characters that can be scored for *Cryptovaranoides*, there is one undoubted plesiomorphy and three other, likely plesiomorphies. In contrast, there are nine plesiomorphies found in *Huehuecuetzpalli*. Whereas all 38 characters are scored for *Cryptovaranoides*, fourteen cannot be scored for *Huehuecuetzpalli* due to a lack of preservation in its fossils; between twelve and twenty-four characters cannot be scored in the Triassic or Jurassic pan-lepidosaurs *Megachirella, Taytalura* and *Marmoretta*. Most characters can be scored for the well described *Gephyrosaurus bridensis* (*11,21*) but only three apomorphies are confidently recorded (although the occasional fused premaxillae are also found) whereas there are thirty plesiomorphies in the taxon. These figures show the remarkable preservation of *Cryptovaranoides* and its importance as a Late Triassic squamate.

Appendix S1. Scores the parsimony-based cladistic phylogenetic analyses. Three runs of the Simões et al. *(10)* taxon-character matrix, with *Cryptovaranoides*, and re-codings of *Megachirella* and *Marmoretta.* We ran similarly with data from *(11)* but did not change their codings for *Megachirella* or *Marmoretta*. Abbreviations: CI, consistency index; HI, homoplasy index; RC, rescaled consistency index; RI, retention index.

Simões et al. (*10*) data matrix

PAUP parsimony: Score of best tree = 1372; Number of trees retained = 9; CI = 0.2208; HI = 0.77932; RI = 0.5847; RC = 0.1291.

TNT parsimony, 82 uninformative characters deleted (Fig. S5A): Score of best tree = 1380; Number of trees retained = 26; Number of trees after TBR branch-swapping = 26; CI = 0.217; RI = 0.577; standard bootstraps from 1000 replicates; Bremer supports from 121,597 trees.

TNT new technology, 82 uninformative characters deleted (Fig. S5B): Score of best tree = 1380; Number of trees retained = 26; Number of trees after TBR branch-swapping = 26; CI = 0.219; RI = 0.581; standard bootstraps from 10,000 replicates; Bremer supports from 121,664 trees.

Griffiths et al. (*11*) data matrix

TNT parsimony (Fig. S6A): Score of best tree = 2421; Number of trees retained = 117; Number of trees after TBR branch-swapping = 117; CI = 0.164; RI = 0.597; standard bootstraps from 1000 replicates; Bremer supports from 792,836 trees.

TNT new technology (Fig. S6B): Score of best tree = 2420; Number of trees retained = 110; Number of trees after TBR branch-swapping = 110; CI = 0.164; RI = 0.598; standard bootstraps from 10,000 replicates; Bremer supports from 18,461,116 trees.

Appendix S2. Character list from Simões et al. *(10)*, with modifications. We have scored *Cryptovaranoides microlanius, Vellbergia bartholomai* (data from *33*) and *Taytalura alcoberi (32)* into the taxon-character matrix of Simões et al. *(10)*. The details of the characters and their original derivation and authors is given in the comprehensive supplementary information of (*10*), and we refer readers to that account. We have not altered any character (e.g., we have left the term basisphenoid rather than sphenoid that we use in our text), and we have made decisions about the character state based principally on the *Cryptovaranoides microlanius* holotype. However, evidence for a character state has been enhanced by reference to some isolated *Cryptovaranoides* bones which are found in the collection and are identifiable by a direct comparison with the holotype skeletal elements; we mention such cases in the coding decisions noted below. We have added one character, 348, referencing the median tooth in the premaxilla of Squamata as this is a character readily scored in squamates, pan-squamates and pan-lepidosaria.

Premaxillae

1. Premaxillae, fusion: unfused (0)/ fused (1).
   We have coded as 0 and 1 as we have separate juvenile premaxillae present in the scan (Fig. 1B) and a fused premaxilla from a larger individual (Fig. 4B, C). It is likely that premaxillae are fused in adult *Cryptovaranoides* but we code for the two states here to include both specimens.
2. Premaxillae, nasal process: present (0)/ absent (1).
   The nasal process is clearly present (Fig. 1B) so coded as 0
3. Premaxillae, posterodorsal process: absent (0)/ present (1).
   A posterodorsal process is absent (Fig. 1B) so coded as 0
4. Premaxillae, dentition: present (0)/ absent medially only (1)/ entirely absent (2).
   Three or four teeth are present on each premaxilla (Figs. 1B, 4B, C) so coded as 0.
5. Premaxillae, dentigerous beak: absent (0) / present (1).
   A dentigerous beak is absent (Fig. 1B) so coded as 0.
6. Premaxillae, ventral bony beak: absent (0)/ present (1).
   A ventral bony beak is absent (Fig. 1B) so coded as 0.
7. Premaxillae, incisive process: absent (0)/ present (1)
   The scan image is not sufficiently detailed in the region (Fig. 1B) so an incisive process cannot be confirmed on the juvenile but an incisive process is present on each of the two fused premaxilla of a larger specimen (Fig. 4B) so this is coded 1.
8. Premaxillae, ventral surface, premaxillary foramina: absent (0)/ present (1).
   The scan is insufficiently detailed in the region (Fig. 1B) but foramina are clear on the larger fused premaxilla (Fig. 4 B) so this is coded as 1.
9. Premaxillae, vomerine medial flange: absent (0)/ present (1).
   A vomerine medial flange is not found in the scan but the image is not sufficiently detailed in the region (Fig. 1B) and the fact that the individual is a juvenile makes the coding unsure so coded as ? Posterior processes are found on the single larger, fused premaxilla but it is unclear if these constitute a vomerine medial flange.

Septomaxillae

10. Septomaxillae: present (0)/ absent (1).
    One (right) septomaxilla is preserved (Fig. 1B) so coded as 0.
11. Septomaxillae, position: on narial margin (0)/ within nasal capsule (1).

The septomaxilla is preserved close to its life position but slightly disarticulated from the right vomer and right maxilla (Fig. 1B). Facets on the septomaxilla match those on the maxilla so it is likely that the septomaxilla was present within the nasal capsule. However, as we cannot be certain we code as ?.

12. Septomaxillae, shape, anteriorly: flat (0)/ convex dorsally (1)/ convex ventrally (2)/ laterally compressed (3).
    The septomaxilla scan image is not sufficiently clear, or in life position (Fig. 1B), to be sure although in does appear to be convex dorsally so probably does roof a vomeronasal organ; but as we cannot be certain we code as ?.
13. Septomaxillae, midline crest, dorsal projection: absent (0)/ present (1).
    The septomaxilla scan image is not sufficiently clear, or in life position (Fig. 1B), to be sure if it does form a midline crest; although we think that it is unlikely to form a midline crest, we cannot be certain so we code as ?.

Maxillae

14. Maxillae, contact, with premaxilla: syndesmotic (0)/ sutural (1).
    The contact between the maxilla and premaxilla appears to be loose, based on the premaxillary facet of the maxilla (Fig. 3C) and the simplicity of the maxillary facet of the premaxilla. This indicates that movement between the bones is likely and therefore we have tentatively coded this as 0.
15. Maxilla-premaxilla fenestra, ventrally: absent (0)/ present (1).
    From observations of the ventral region of the maxilla (Fig. 3B) and with the maxilla/premaxillary contact established from isolated bones and the CT scan this character is absent so coded as 0.
16. Maxillae, anterior superior alveolar foramen: absent (0)/ present (1).
    This foramen is certainly present (Fig. 3A) so coded as 1.
17. Maxillae, nasal process: absent (0)/ present (1).
    The nasal process is certainly present (Figs. 1B, E, 3A–C) so coded as 1.
18. Maxillae, posterior emargination, between nasal and orbital processes: absent (0)/ present (1).
    There is no antorbital fenestra and no emargination related to that feature (Fig. 2A) so coded as 0.
19. Maxillae, antorbital fossa: absent (0)/ present (1).
    There is no antorbital fossa (Fig. 2A) so coded as 0.
20. Maxillae, premaxillary process: present (0)/ absent (1).
    A premaxillary process is present on the maxilla (Figs. 1B, 2A) so coded as 0.
21. Maxillae, premaxillary process, groove, on dorsal surface: absent (0)/ present (1).
    There is a groove on the dorsal surface, but it is unlike previous figures (*22*) so we have provisionally coded this feature as ?

Nasals

22. Nasals, fusion: paired (0)/ fused (1).
    Although some fragments of bone may derive from the nasal, an identifiable element is not preserved in the holotype. Therefore, we code this feature as ?.
23. Nasals, ventrolateral process: absent (0)/ present (1).
    An identifiable nasal is not preserved in the holotype. Therefore, we code this feature as ?.
24. Nasals, ventrolateral process, position, relative to maxillary nasal process: posteriorly (0)/

anteriorly (1)/ dorsally (2).
An identifiable nasal is not preserved in the holotype. Therefore, we code this feature as ?.
25. Nasals, foramina: absent (0)/ present (1).
An identifiable nasal is not preserved in the holotype. Therefore, we code this feature as ?.
26. Nasals, ventromedial crest: absent (0)/ present (1).
An identifiable nasal is not preserved in the holotype. Therefore, we code this feature as ?.

Lacrimals and Prefrontals
27. Lacrimals: present (0)/ absent (1).
Both lacrimals are represented in the holotype (e.g., Fig. 1E) so this is coded as 0.
28. Lacrimals, position, relative to prefrontal lateral margin: ventral (0)/ anterior (1)/ posterior (2).
The lacrimal lies ventrally to the prefrontal (main paper Fig 1 E) so is coded as 0.
29. Lacrimal duct, foramen, division: single (0)/ double (1).
The lacrimal duct appears to be single in *Cryptovaranoides* (Fig. 1E) so is coded as 0.
30. Lacrimal duct, posterior opening on skull surface, position: posteriorly (0)/ laterally (1).
The lacrimal duct appears to open posteriorly (Fig 1E) so is coded as 0.
31. Lacrimals, shape, curved anteriorly: absent (0)/ present (1).
Simões uses this character to exclude reptiles with an antorbital fenestra: 'This anterior curvature usually occurs in taxa with an antorbital fenestra, in which the lacrimal has a “T” shape in lateral view.’ The lacrimal is not curved anteriorly as there is no antorbital foramen (Fig 1E) so is coded as 0.
32. Prefrontals, ornamentation on external surface: absent (0)/ rugosities (1)/ tubercles (2)/ pits (3).
From the scan both left and right bones appear rugose; also, isolated prefrontal bones are rugose, so this is coded as 1.
33. Prefrontal crest: absent (0)/ present (1).
No crest is present on the prefrontal (Fig. 1B, E) so this is coded as 0.

Supraorbitals (= palpebrals) and Jugals
34. Supraorbital bones: absent (0)/ present (1).
Palpebrals may have been absent or present but we have no sure evidence either way so we code this as ?.
35. Jugals: present (0)/ absent (1).
Both jugals are present in the holotype (Fig. 1E, F) so this is coded as 0.
36. Jugals, posteroventral process: absent (0)/ present (1).
The jugal of *Cryptovaranoides* has an abrupt posterior termination (Fig. 1F) which might be considered a rudimentary process, but we deem it insufficient to constitute a posteroventral process so is coded as 0.
37. Jugals, posteroventral process, shape: separate from dorsal process (0)/ connected to dorsal process by bony flange (1).
As we do not consider that a posteroventral process is present we code the shape as a missing character (-).

Quadratojugals
38. Quadratojugals: present (0)/ absent (1).

Quadratojugals are certainly absent in the holotype, and are also absent in an isolated quadrate specimen, so this is coded as 1.

39. Quadratojugals, anterior extension: present (0)/ absent (1).
As the quadratojugal is absent, we code this inapplicable character as (-).
40. Quadratojugals, ventral margin, medially inflected flange: absent (0)/ present (1).
As the quadratojugal is absent, we code this inapplicable character as (-).
41. Quadratojugals, ornamentation, on external surface: absent (0)/ pits (1)/ rugosities (2)/ striations (3)/ tubercles (4).
As the quadratojugal is absent, we code this inapplicable character as (-).
42. Quadratojugal foramen: absent (0)/ present (1).
As the quadratojugal is absent, we code this inapplicable character as (-).

Postorbitals

43. Postorbitals: present (0)/ absent (1).
We regard the bone positioned just above the right jugal as the postorbital (Fig. 1F), so have coded this as 0.
44. Postorbitals, fusion to postfrontal: unfused (0)/ fused (1).
From the evidence of the facets (Fig. 7C) the postorbital is unfused to any bone so this is coded 0.
45. Postorbitals, dorsal margin, position relative to postfrontal: laterally (0)/ posteriorly (1)/ anteriorly (2).
We have no evidence of the postorbital position relative to any postfrontal, so code this as ?.
46. Postorbitals, dorsal process: absent (0)/ present (1).
We have no evidence of an absent/present postorbital dorsal process so code this as ?.
47. Postorbitals, ventral process: absent (0)/ present (1).
There is a clear ventral process on the postorbital (Figs. 1G, Fig. 7C) which has a facet that we suggest is for the jugal dorsal process, so this is coded 1.
48. Postorbitals, dorsal concavity: absent (0)/ present (1).
Although this appears to be lacking in *Cryptovaranoides* (Fig. 1F) we cannot be certain, so we code this as a ?.

Squamosals

49. Squamosals: present (0)/ absent (1).
A squamosal has been identified (Fig. 1B) so this is coded 0.
50. Squamosals, anteroventral process: absent (0)/ present (1).
The tetraradiate squamosal of the rhynchocephalians is absent (Fig. 1B) so this is coded 0.
51. Squamosals, posterior process: absent (0)/ present (1).
The *Cryptovaranoides* squamosal has a posteroventral process characteristic of squamates (Fig. S1A) so this is coded 1.
52. Squamosals, dorsal process: absent (0)/ present (1).
The dorsal process of the squamosal is present in the holotype (Fig. 1B) so is coded as 1.
53. Squamosals, occipital flange: present (0)/ absent (1).
The occipital flange of the squamosal as described (10) is absent in the holotype (Fig. 1B) so is coded as 1.
54. Squamosals, ventral margin, medial inflection: absent (0)/ present (1).
A medial inflection of the ventral margin squamosal is absent in the holotype (Fig. 1B) so is coded as 0.

55. Squamosals, anterior margin, bifid facet for postorbital: absent (0)/ present (1).
The squamosal lacks the anterior bifid facet (Fig. 1B) so is coded 0.

Postfrontals

56. Postfrontals: present (0)/ absent (1).
Although a facet for the presumed postfrontal is present on the frontal the postfrontal is not preserved or is unrecognized so this is coded ?.
57. Postfrontals, distal process: present (0)/ absent (1).
The postfrontal is not preserved or is unrecognized so this is coded ?.
58. Postfrontals, distal process, division: single (0)/ double (1).
The postfrontal is not preserved or is unrecognized so this is coded ?.
59. Postfrontals, medial margin, position, relative to parietal: ventral (0)/ dorsal (1)/ lateral (2)/ anterior (3).
The postfrontal is not preserved or is unrecognized so this is coded ?.
60. Postfrontals, parietal process: absent (0)/ present (1).
The postfrontal is not preserved or is unrecognized so this is coded ?.
61. Postfrontals, medial forking: absent (0)/ present (1).
The postfrontal is not preserved or is unrecognized so this is coded ?.

Supratemporals, Tabulars and Postparietals

62. Supratemporals: absent (0)/ present (1).
A supratemporal has been identified (Fig. 1B) and this character is coded as 1.
63. Supratemporals, temporal process: absent (0)/ present (1).
The preserved supratemporal lacks a temporal process so this is coded as 0.
64. Tabulars: present (0)/ absent (1).
A tabular has not been recognized and is likely to be absent but as we are not certain this is coded as ?.
65. Postparietals: present (0)/ absent (1).
A postparietal has not been recognized and is likely to be absent but as we are not certain this is coded as ?.
66. Postparietals, number: single (0)/ paired (1).
A postparietal has not been recognized and is likely to be absent but as we are not certain this is coded as ?.

Frontals

67. Frontals, fusion to each other: unfused (0)/ fused (1).
Although frontals are not preserved in the holotype, isolated frontals are found in the same matrix (Fig. 7F-H). They are confirmed as belonging to *Cryptovaranoides* because their prefrontal facets match the equivalent on the scanned prefrontal. The frontals are in paired elements, so this is coded as 0.
68. Frontals, parietal tabs: absent (0)/ present (1).
The frontals lack the parietal tabs found in rhynchocephalians, so this is coded as 0.
69. Frontals, subolfactory processes: absent (0)/ present (1).
Subolfactory processes (cristae cranii) are present on the frontals so this is coded as 1.
70. Frontals, subolfactory processes, fusion to each other: absent (0)/ present (1).
The subolfactory processes are not fused so this is coded as 0.
71. Frontals, orbitonasal projection: absent (0)/ present (1).

The isolated frontals have an anterior projection bordering the prefrontal, so this is coded as 1.

Parietals

72. Parietals, fusion: unfused (0)/ fused (1).
    Parietals are not preserved in the holotype or any isolated bones, so this is coded as ?.
73. Pineal foramen: present (0)/ absent (1).
    There is no indication in the posterior margin of the frontal of a pineal foramen so it is likely to have been positioned in the parietal(s) but as we cannot confirm this, we code this as a ?.
74. Parietals, supratemporal process: absent (0)/ present (1).
    Parietals are not preserved in the holotype or any isolated bones, so this is coded as ?.
75. Parietals, supratemporal process, distal end, division: single (0)/ bifid (1).
    Parietals are not preserved in the holotype or any isolated bones, so this is coded as ?.
76. Parietals, ornamentation: absent (0)/ rugosities (1)/ tubercles (2)/ pits (3).
    Parietals are not preserved in the holotype or any isolated bones, so this is coded as ?.
77. Parietals, lateral frill: absent (0)/ present (1).
    Parietals are not preserved in the holotype or any isolated bones, so this is coded as ?.
78. Parietals, frontal tabs of parietal: absent (0)/ present (1).
    Parietals are not preserved in the holotype or any isolated bones, so this is coded as ?.
79. Parietals, nuchal fossa: absent (0)/ present (1).
    Parietals are not preserved in the holotype or any isolated bones, so this is coded as ?.
80. Parietals, nuchal fossa, roofing by parietal posterior flange: unroofed (0)/ roofed (1).
    Parietals are not preserved in the holotype or any isolated bones, so this is coded as ?.
81. Parietals, ventral side, parietal fossa: present (0)/ absent (1).
    Parietals are not preserved in the holotype or any isolated bones, so this is coded as ?.
82. Parietals, ventral side, parietal fossa, posterior margin: open (crests extend posterolaterally) (0)/ closed (crests meet at midline) (1).
    Parietals are not preserved in the holotype or any isolated bones, so this is coded as ?.
83. Parietals, posteromedial (= postparietal) process: absent (0)/ present (1).
    Parietals are not preserved in the holotype or any isolated bones, so this is coded as ?.
84. Parietals, posteromedial (= postparietal) process, division: single (0)/ bifid (1).
    Parietals are not preserved in the holotype or any isolated bones, so this is coded as ?.
85. Parietals, parietal table, shape: margins ventrally directed, sagittal crest present (0)/ margins ventrally directed, without sagittal crest (1)/ margins laterally directed (2).
    Parietals are not preserved in the holotype or any isolated bones, so this is coded as ?.
86. Parietals, dorsal surface, parasagittal crests: absent (0)/ present (1).
    Parietals are not preserved in the holotype or any isolated bones, so this is coded as ?.
87. Parietals, crista cranii parietalis, epipterygoid process: absent (0)/ present (1)). Parietals are not preserved in the holotype or any isolated bones, so this is coded as ?.

Vomers

88. Vomers, fusion: unfused (0)/ fused (1).
    Vomers are unfused (Fig. 7A, S1E) so coded as 0.
89. Vomers, teeth: absent (0)/ present (1).
    Teeth are present on the vomers (Fig. 7A) so coded as 1
90. Vomers, anterior premaxillary process: absent (0)/ present (1).

The vomerine premaxillary process is present (Fig. 7A) although we cannot confirm that it divides an incisive process, but there is an indication that there is a separation on the premaxillae (Fig. 4B), so we tentatively code this character as 1.

91. Vomers, lateral expansion: absent (0)/ present (1).
    The vomers are laterally expanded (Fig. 7A) and have a facet which attached to the vomerine facet of the maxilla. The condition is neochoanate. This character is coded as 1.
92. Vomers, ventral surface, midline crest (=longitudinal ridge): absent (0)/ present (1).
    Ridges are present on the vomers, but a midline crest is absent (Fig. 7A), so this is coded as 0.
93. Vomers, ventral surface, lateral crest (=longitudinal ridge): absent (0)/ present (1).
    A lateral crest, or more accurately a ridge (Fig. 7A) runs parallel anteriorly, so this is tentatively coded as 1.
94. Vomers, ventral foramina, in each vomerine element: present (0)/ absent (1).
    The scan does not provide sufficient resolution to decide if a central foramen is present in each vomer so this is coded as ?.
95. Vomers, shape in cross-section: flat (0)/ convex ventrally (1).
    This is difficult to code as there is convexity present (Fig. 7A) but other areas are relatively flat so it is coded as ?.
96. Vomers, posteroventral process (=descending tubercle): absent (0)/ present (1).
    A posteroventral process is unlikely to be present but the scan does not provide sufficient information to be sure so this character is coded as ?.

Palatines

97. Palatines, teeth: absent (0)/ present (1).
    Teeth are present on the palatines (Fig. 7D, E) so coded as 1.
98. Palatines, ascending process: absent (0)/ present (1).
    No ascending process is observed in the scans, so this feature is coded as 0.
99. Palatines, dorsomedially directed process: absent (0)/ present (1).
    No dorsomedial process is observed in the scans, so this feature is coded as 0.
100. Palatines, ventral surface, sulcus choanalis: absent (0)/ present (1).
    A sulcus choanalis (or choanal groove or fossa) is present (Figs. 7D, E, S1E) and is well-developed on large specimens in the collection this character is coded as 1.
101. Palatines, infraorbital foramen: present (0)/ absent (1).
    The scan is insufficiently detailed to decide if an infraorbital foramen is present (although it is likely) and this character is coded as ?.
102. Palatine foramen: absent (0)/ present (1).
    The scan is insufficiently detailed to decide if a palatine foramen is present so this character is coded as ?.
103. Palatines, maxillary process, ventral aspect: absent (0)/ present (1).
    The scan is insufficiently detailed to decide if a maxillary process is present as depicted in Gauthier et al. (*22*) so this character is coded as ?.
104. Palatines, subchoanal shelf: absent (0)/ present (1).
    A subchoanal shelf (secondary palate of *17*) is absent (Figs. 7D, E) so this is coded as 0.

Pterygoids

105. Pterygoids, teeth: absent (0)/ present (1).
    Pterygoid teeth are present (Fig. 1B, 2D) so this is coded 1.
106. Pterygoids, anteromedial processes, anterior end division: single (0)/ bifurcate (1).

The pterygoid anteromedial process appears to be single in the CT scans (Fig. 1B), so this is coded 0.

107. Pterygoids, transverse processes: absent (0)/ present (1).
A pterygoid transverse process is present (Figs. 1B, S1E) so this is coded 1.

108. Pterygoids, transverse processes, flange: absent (0)/ present (1).
A flange on the pterygoid transverse process is present (Figs. 1B, S1E) so this is coded 1.

109. Pterygoids, transverse process, teeth: absent (0)/ present (1).
From the scans there are no teeth on the pterygoid transverse process (Figs. 1B, S1E) so this is coded 0.

110. Pterygoids, main body, concave in ventral aspect: absent (0)/ present (1).
From the scan data the main body is concave in ventral aspect (Figs. 1B, S1E) so this is coded 1.

111. Pterygoids, arcuate flange: absent (0)/ present (1).
An arcuate flange of the type seen in early diapsids or some sphenodontians is absent (Figs. 1B, S1E) so this is coded as 0.

112. Pterygoids, quadrate rami, posterolateral excavation: absent (0)/ present (1).
A posterolateral excavation of the pterygoid quadrate ramus is probably absent but the scan does not provide sufficient detail so this is coded as ?.

Ectopterygoids

113. Ectopterygoids: absent (0)/ present (1).
Ectopterygoids are present (Fig. 1B) so this is coded as 1.

114. Ectopterygoids, lateral process: absent (0)/ present (1).
A lateral process of the ectopterygoid is present (Fig. 1B) so this is coded as 1.

Epipterygoid

115. Epipterygoid: present (0)/ absent (1).
A possible epipterygoid is present and there is a notch in the dorsal region of the pterygoid (Fig. S1H) which indicates its presence, so this is coded as 0.

116. Epipterygoid, base shape: base flared out (0)/ base columnar (1).
The scan is insufficiently distinct to decide the nature of the epipterygoid base so this is coded as ?.

Quadrate

117. Quadrates, articulating surface: flat (0)/ with condyles (1).
From the scan, although incompletely preserved, there are condyles indicated on the quadrate and additionally the articular surface of the lower jaw has cotyles which confirm their presence. Condyles are present in isolated quadrates, so this is coded as 1.

118. Quadrate foramen: present (0)/ absent (1).
The quadrate in the holotype and in isolated bones (Figs. 1F, 6D-F) have no quadrate foramen so this is coded as 1.

119. Quadrates, pterygoid process: present (0)/ absent (1).
A quadrate pterygoid process is absent (Fig. 6D–F); this absence is also observed in an isolated quadrate), so this is coded as 1.

120. Quadrates, posterior emargination: absent (0)/ present (1).
A posterior emargination of the quadrate is present (Fig. 1C); and is also observed in an isolated quadrate) so this is coded as 1.

121. Quadrates, quadrate conch: absent (0)/ present (1).
A quadrate conch is present (Figs. 1C, F, 6D); it is also observed in an isolated quadrate), so this is coded as 1.
122. Quadrates, suprastapedial process: absent (0)/ present (1).
A suprastapedial process of the quadrate is absent (no ventral deflection of the cephalic head of the quadrate; (Fig. 6D), so this is coded as 0.
123. Quadrates, cephalic condyle, notch for the squamosal: absent (0)/ present (1).
A squamosal notch is present on the cephalic head of the quadrate is present (Fig. 1C, F, 6D–F); it is also observed in an isolated quadrate), so this is coded as 1.

Braincase
124. Carotid foramina, braincase, position: lateral wall of braincase (0)/ ventral surface of braincase (1).
From the scans, direct images (Fig. 5B) the carotid foramina are in the lateral wall of the braincase and not ventrally as in *Gephyrosaurus* Evans (*21*, fig. 34 c), so this is coded as 0.

Supraoccipital
125. Supraoccipital, lateral ascending processes: absent (0)/ present (1).
The supraoccipital preserved in a separate specimen (Fig. 4D) does not have an ascending lateral process so this is coded 0.
126. Supraoccipital, medial ascending process: absent (0)/ present (1).
The preserved supraoccipital has a medial ascending process (Fig. 4D) so this is coded 1.
127. Supraoccipital, fusion to exoccipitals: unfused (0)/ fused (1).
Although it is likely, we cannot be sure if the preserved supraoccipital is fused to the exoccipital as a fracture indicates a suture line (Fig. S1D) so this is coded ?.
128. Supraoccipital, sagittal crest: absent (0)/ present (1).
The sagittal crest described for the supraoccipital (*10*) is absent, so this is coded 0.
129. Supraoccipital, lateral nuchal crest: absent (0)/ present (1).
The nuchal crest described for the supraoccipital (*22*) is present, so this is coded 1.

Basioccipital
130. Basioccipital/basisphenoid, sphenoid tubercles: absent (0)/ present (1).
Sphenoid tubercles are present on the basioccipital (Fig. 1D), so this is coded as 1.
131. Sphenooccipital epiphyses: absent (0)/ present (1).
Although it is unlikely that sphenoccipital epiphyses are present we do not have sufficient evidence from the scan or direct observations to confirm so this is coded as ?.
132. Basioccipital, fusion, to exoccipital: unfused (0)/ fused (1).
On the braincase (Fig. 4 D, E) there is a partial fusion of the two bones which is incomplete but in general basioccipital and exoccipital are separate as shown by facets on the large basioccipital (Figs. 1C, 7B) on the holotype rock so this is coded as 0.
133. Basioccipital, fusion to basisphenoid: unfused (0)/ fused (1).
The basioccipital and basisphenoid are separate (Fig. 1D) in the holotype fossil so this is coded as 0.
134. Basioccipital, ventral aspect, shape, concavity: single (0)/ divided (1)/ absent (2).
We have coded this 0 as the ventral aspect has no concavity.

Basisphenoid

135. Basisphenoid, Vidian canal: open (0)/ fully enclosed (1).
The vidian canal is fully closed within the basisphenoid as it crosses the basipterygoid process (Fig. 5E, F, H, I) so this is coded as 1.
136. Basisphenoid, basipterygoid processes: present (0)/ absent (1).
Basipterygoid processes are present on the basisphenoid (Fig. 5F) so this is coded 0.
137. Basisphenoid, dorsum sellae: absent (0)/ present (1).
A dorsum sella is present on the basisphenoid (Fig. 5A)) so this is coded 1.
138. Basisphenoid (or fused parabasisphenoid), ventral aspect, shape, concavity: single (0)/ divided (1)/ absent (2).
We have coded this 0 as the ventral aspect (Fig. 5C) has no concavity.
139. Basisphenoid, lateral depression: absent (0)/ present (1).
A lateral depression of the basisphenoid is absent so this is coded as 0.

Prootics
140. Prootics, prootic crest: absent (0)/ present (1).
The preserved prootic on the isolated braincase has a crest so this is scored as 1.
141. Prootics, prootic crest, shape: crest (0)/ curved flange (1).
The preserved prootic on the isolated braincase broken in this region so this is coded ?.
142. Prootics, alar crest: absent (0)/ present (1).
The preserved prootic on the isolated braincase appears to have the base of an alar crest but as it is broken in this region it is coded ?.
143. Prootics, supratrigeminal process: absent (0)/ present (1).
The Prootic of the isolated braincase is broken in this region so this is coded as ?.
144. Prootics, anterior inferior process: present (0)/ absent (1).
A discernible anterior inferior process is present so this is coded as 0.
145. Prootics, lateral wall, facial foramen: absent (0)/ present (1).
We cannot discern if a facial foramen is present on the lateral wall of the prootic so this is coded as ?.
146. Prootics, lateral wall, facial foramen, division: single (0)/ double (1).
As we cannot discern the facial foramen of the prootic this is coded as ?.

Parasphenoid
147. Parasphenoid, teeth: absent (0)/ present (1).
Teeth are absent on the parasphenoid so this is coded as 0.
148. Parasphenoid, orbitosphenoid processes: absent (0)/ present (1).
There is insufficient detail on the scans to confirm the presence/absence of an orbitosphenoid process so this is coded as ?.

Opisthotics and Exoccipitals
149. Opisthotics, crista interfenestralis: present (0)/ absent (1).
The isolated braincase specimen indicates the presence of a crista interfenestralis (Fig. 4E, I) so this is coded as 0.
150. Exoccipitals, lateral flange: absent (0)/ present (1).
The exoccipitals are fused to the opisthotics so this character is recorded as missing (-).
151. Exoccipitals, fusion: unfused (0)/ to opisthotics only (1)/ to opisthotics and prootics (2).

The isolated braincase specimen confirms that the exoccipitals are fully fused to the opisthotics only so this is coded 1.

152. Exoccipitals, occipital condyle process: absent (0)/ present (1).

It is clear from the isolated braincase specimen that the exoccipitals contribute to the occipital condyle (Fig. 4D), so this is coded 1.

153. Exoccipitals, crista tuberalis: absent (0)/ present (1).

It is clear from the isolated braincase specimen that a crista tuberalis is present (Fig. 4I) on the exoccipitals, so this is coded 1.

154. Exoccipitals (or otooccipitals), contact to each other, medially: absent (0)/ present (1).

It is clear from the isolated braincase specimen (Fig. 4D) that the otooccipitals do not contact each other so this is coded 0.

Stapes (= columella)

155. Stapes, dorsal process: present (0)/ absent (1).

We can find no evidence of a preserved stapes on the scan so this is coded as ?.

156. Stapes, stapedial foramen: present (0)/ absent (1).

We can find no evidence of a preserved stapes on the scan so this is coded as ?.

Laterosphenoid (= pleurosphenoid) and Orbitosphenoid (= postoptic)

157. Laterosphenoids: absent (0)/ present (1).

We can find no evidence of a preserved laterosphenoid on the scan so this is coded as ?.

158. Orbitosphenoids: absent (0)/ present (1).

We can find no evidence of a preserved orbitosphenoid on the scan so this is coded as ?.

159. Orbitosphenoids, fusion to each other: unfused (0)/ fused medially (1).

We can find no evidence of a preserved laterosphenoid on the scan so this is coded as ?.

Sphenethmoid

160. Ossified sphenethmoid: present (0)/ absent (1).

We can find no evidence of a preserved sphenethmoid on the scan so this is coded as ?.

161. Ossified sphenethmoid, shape: without orbital septum (0)/ with orbital septum also ossified (1) / only orbital septum (2).

We can find no evidence of a preserved sphenethmoid on the scan so this is coded as ?.

Mandibles

162. Anterior mylohyoidal foramen: absent (0)/ present (1).

An anterior mylohyoidal foramen is present on the mandible and located in the splenial (Fig. S1C) so this is coded 1.

163. Posterior mylohyoidal foramen: absent (0)/ present (1).

A posterior mylohyoidal foramen is present on the mandible and located at the junction of the splenial and angular (Fig. S1C) so this is coded 1.

164. Anterior inferior alveolar foramen: absent (0)/ present (1).

An anterior inferior alveolar foramen is (probably) present on the mandible and located in the splenial (Fig. S1C) so this is tentatively coded 1.

Dentaries

165. Dentaries, symphyses, fusion to each other: unfused (0)/ fused (1).

The dentaries are unfused at the symphysis (Fig. 3E), so this is coded as 0.

166. Dentaries, symphysial area, shape: flat (0)/ convex (1).
The symphysial area appears to be essentially flat (Fig. 3E) so this is coded as 0.
167. Dentaries, anterior end, split by Meckelian canal: absent (0)/ present (1).
The symphysial area is partially split by the Meckelian canal (Fig. 3E) but the dorsal and ventral surfaces are continuous so this is coded as 0. Simões et al. (*10*) note 'However, even in cases where the Meckelian canal is open throughout most of the extension of the dentary, the anterior tip of the dentary is still formed by a contact between its dorsal and ventral margins (that frame the Meckelian canal), as seen in most squamates. In some reptiles, however, this anterior end is split (or indented) by the Meckelian canal, which becomes open anteriorly.'—so in this case as the dorsal and ventral margins are continuous and frame the Meckelian canal that is 0.
168. Dentaries, anterior end, symphysial articulatory facet, position: on dorsal margin only (0)/on dorsal and ventral margins (1)/ on ventral margin only (2).
The articulatory facet is positioned on both dorsal and ventral symphysial areas (Fig. 3E) so this is coded as 1.
169. Dentaries, subdental shelf: present (0)/ absent (1).
A dentary subdental shelf is present (Fig. 3E, G) so this is coded as 0.
170. Dentaries, dorsal margin, contact, with ventral margin in medial view: absent (0)/ present (1).
The dorsal and ventral margins of the dentary have no medial contact (Fig. 3E) so this is coded as 0.
171. Dentaries, dorsal margin, fusion, with ventral margin in medial view: absent (0)/ present (1).
The dorsal and ventral margins of the dentary are separate (Fig. 3E) so this is coded as 0.
172. Dentaries, coronoid process: absent (0)/ present (1).
There is a discernible (though rudimentary) edentulous coronoid process of the dentary (Fig. 3E) so we tentatively code this as 1.
173. Dentaries, coronoid process, dorsal expansion: absent (0)/ present (1).
The coronoid process of the dentary has some height (Fig. 3E) but is not significantly dorsally expanded so this is tentatively coded as 0.
174. Dentaries, coronoid process, division: single (0)/ double (1).
The coronoid process of the dentary is single (Fig. 3E), so this is coded as 0.
175. Dentaries, posteroventral process: absent (0)/ present (1).
There is no significant posteroventral process (Figs. 1F, S1B) below the level of the surangular/dentary contact (where the anterior surangular foramen would be positioned, but that is not clear in the scan) so this is coded as 0.

Splenials

176. Splenials: absent (0)/ present (1).
A splenial is present (Figs. 1A, E, S1C) so this is coded as 1.
177. Splenials, fusion to dentary: unfused (0)/ fused (1).
The splenial is not fused to the dentary (Figs. 1E, S1C) and there are splenial facets on the dentary so this is coded as 0.
178. Splenials, symphysial process: absent (0) / present (1).
The splenial facets of the dentary (Figs. 3E, S1C) do not appear to reach the symphysis so this is tentatively coded as 0.
179. Splenials, anterior border, shape: rounded (0)/ notched (1)/ flat (2)/ tapering (3).

The anteriormost region of the splenial is not preserved and although facets on the dentary indicate that it is likely to have tapered we cannot be sure so this is coded as ?.

Angulars

180. Angulars: present (0)/ absent (1).
An angular is present (Fig. 1E) so this is coded as 0.

181. Angulars, anterior end, medial view, position relative to splenial: lateral (0)/ dorsal (1)/ ventral (2)/ posterior (3).
The anterior end of the angular is positioned ventrally to the splenial (Figs. 1E, S1C) in medial view so this is coded as 2.

Surangulars

182. Surangulars, coronoid process: absent (0)/ present (1).
The surangular has a coronoid process (Figs. 1E, F, S1B, C) that reaches higher than the crowns of the mandibular teeth, so this is coded as 1.

183. Surangulars, lateral adductor crest: absent (0)/ present (1).
The lateral adductor crest of the surangular is present (Figs. 1C, S1C) so this is coded as 1.

184. Surangulars, anterior surangular foramen: absent (0)/ present (1).
The scan is insufficiently detailed to decide if the anterior surangular foramen is present so this is coded with a ?

185. Surangulars, posterior surangular foramen: absent (0)/ present (1).
The scan is insufficiently detailed to decide if the posterior surangular foramen is present so this is coded with a ?.

186. Surangulars, mandibular fenestra: absent (0)/ present (1).
A surangular fenestra is absent (Fig. 1C, F) so this is coded as 0.

Articulars

187. Articulars, lateral shelf: absent (0)/ present (1).
There does not appear to be a lateral shelf on the articular (Figs. 1C, F, 6G, H) so this is coded as 0.

188. Articulars, fusion: unfused (0)/ to prearticular only (1)/ to prearticular + surangular only (2)/ to prearticular + surangular + angular only (3)/ to prearticular + surangular + angular + splenial (4).
The scan is insufficiently resolved to decide if fusion is present to the combination of bones named so this is coded with a ?.

189. Articulars, foramen chorda tympani: absent (0)/ present (1).
The scan is insufficiently resolved to decide if a chorda tympani foramen is present so this is coded with a ?.

190. Articulars, retroarticular process: absent (0)/ present (1).
A retroarticular process is present (Figs. 1C, F, S1B, C) so this is coded as 1.

191. Articulars, retroarticular process, dorsal fossa: absent (0)/ present (1).
A dorsal fossa is present on the retroarticular process (Figs. 1C, S1C) so this is coded as 1.

192. Articulars, retroarticular process, lateral notch: absent (0)/ present (1).
A lateral notch is absent on the retroarticular process (Figs. 1F, S1B) so this is coded as 0.

193. Articulars and prearticulars, medial process: absent (0)/ present (1).
A rudimentary medial process (angular process) is present (Figs. 1C, 6G,H, S1C) so this is tentatively coded as 1.

194. Articulars and prearticulars, medial process, prearticular crest: absent (0)/ present (1).
There is no observable crest on the medial process present (Fig. 1C), so this is coded as 0.

Prearticulars

195. Prearticulars, retroarticular process: absent (0)/ present (1).
The contribution, if any, of the prearticular to the retroarticular process is not clear from the scans so this is coded as a ?.

196. Prearticulars, mandibular fossa: present (0)/ absent (1).
The prearticular mandibular fossa is present (Figs. 1C, S1C) so this is coded as 0.

Coronoids

197. Coronoids, dorsal process: absent (0)/ present (1).
The dorsal process on the coronoid is present (Fig. 7I, J) so this is coded as 1.

198. Coronoids, anterolateral (=labial) process: absent (0)/ present (1).
A labial process on the coronoid is present (Fig. 7I and unregistered specimens so this is coded as 1.

199. Coronoids, anteromedial process: present (0)/ absent (1).
An anteromedial process on the coronoid is present (Fig. 7J) so this is coded as 0.

200. Coronoids, posterodorsomedial process: present (0)/ absent (1).
Although there is a posteromedial process of the coronoid (Fig. 7I) it does not lie dorsally so this character is coded as a ?.

201. Coronoid, posteroventromedial process: present (0)/ absent (1).
A posteroventromedial process on the coronoid is present (Fig. 7I) so this is coded as 0.

Dentition

202. Dentition, crown apical striations, labial side: absent (0)/ present (1).
Crown apical striations are absent from the labial side (Fig. 3A, D, G) so this is coded as 0.

203. Dentition, mesiodistal serration: absent (0)/ present (1).
Mesiodistal serrations are absent (Fig. 3F, G) so this is coded as 0.

204. Posterior dentition, accessory cusps, mesiodistally oriented: absent (0)/ present (1).
Mesiodistal accessory cusps are absent (Figs. 3E, 4A) so this is coded as 0.

205. Posterior dentition, accessory cusps, labiolingually oriented: absent (0)/ present (1).
Labiolingually directed accessory cusps are absent (Fig. 3E, 4A) so this is coded as 0.

206. Posterior dentition, replacement teeth: absent (0)/ present (1).
Replacement teeth are present in the posterior dentition of maxillae (Fig. 4A) and dentaries (Fig. 3E) so this is coded as 1.

207. Posterior dentition, resorption pits: present (0)/ absent (1).
Resorption pits are present in the posterior dentition of maxillae (Fig. 4A) and dentaries (Fig. 3E), so this is coded as 0.

208. Posterior dentition, replacement teeth, position in relation to functional teeth: lingual (0)/ posterolingual (1).
Teeth are replaced lingually in the posterior dentition of maxillae (Fig. 4A) and dentaries (Fig. 3E), so this is coded as 0.

209. Posterior dentition, tooth shape, concave anteriorly: absent (0)/ present (1).
The posterior teeth do not have a concave anterior (Figs. 3E, 4A), so this is coded as 0.

210. Posterior dentary teeth, position, relative to dentary crista dorsalis (apex of labial wall) of dentary: lingual (0)/ apical (1)/ apicolingual (2).

The posterior dentary teeth are positioned lingually with respect to the labial wall (Fig. 3E), so this is coded as 0.

211. Posterior dentary teeth, ankylosis to crista dorsalis (apex of labial wall) of dentary: absent (0)/ present (1).
The posterior dentary teeth are not ankylosed to the labial wall apex (Fig. 3E), so this is coded as 0.

212. Posterior dentary teeth, delimitation by tooth bearing bone: by a labial wall only (0)/ by a three-sided socket (1)/ by a four-sided socket (2)/ by a lingual and labial wall only (3).
The posterior dentary teeth are pleurodont, delimited by a labial wall only (Fig. 3E) so this is coded as 0.

213. Posterior maxillary teeth, delimitation by tooth bearing bone: by a labial wall only (0)/ by a three-sided socket (1)/ by a four-sided socket (2)/ by a lingual and labial wall only (3).
The posterior maxillary teeth are, pleurodont, delimited by a labial wall only (Fig. 4A) so this is coded as 0.

214. Posterior dentary dentition, lingual and labial carinae: absent (0)/ present (1),
Carinae are present on the posterior dentary dentition (Fig. 3G) but these are primarily mesio-distal not lingual and labial, so this is coded as 0.

215. Anterior dentary teeth: present (0)/ absent (1).
Anterior dentary teeth are present (Fig. 3E) so this is coded as 0.

216. Anterior dentary teeth, position relative to the jaw apical margin (dentary dorsal crest or maxillary ventral crest): lingual (0)/ apical (1)/ apicolingual (2).
The anterior dentary teeth are positioned lingually with respect to the jaw apex (Fig. 3E), so this is coded as 0.

217. Posterior maxillary teeth, posteromedial ridge: absent (0)/ present (1).
The posterior maxillary teeth (Fig. 3F, 4A) do not have a posteromedial ridge (as in *Kallimodon*) so this is coded as 0.

218. Anterior maxillary teeth, alternating teeth series: absent (0)/ present (1).
The anterior maxillary teeth (Fig. 4A) do not have an alternating size series (as in some sphenodontians) so this is coded as 0.

Atlas and axis

219. Atlas, pleurocentrum, fusion to axis: unfused (0)/ fused (1).
The atlas pleurocentrum is fused to the axis (Fig. 1C) so this is coded as 1.

220. Atlas, neural arches: separate (0)/ sutured to each other (1)/ sutured to axis neural spine (2).
The neural arches of the atlas are separated in the holotype (Fig. 1C) so, although they could possibly fuse in the adult, we tentatively code this as 0.

221. Atlas, neural arches, postzygapophyses: absent (0)/ present (1).
Postzygapophyses are present on the atlas neural arches (Fig. 1C) so this is coded as 1.

222. Atlas, ribs: present (0)/ absent (1).
From the scan data, atlas ribs are absent (Fig. 1C), so this is coded as 1.

223. Axis, pleurocentrum, fusion to neural arch: unfused (0)/ fused (1).
The axis is fused to the pleurocentrum (Fig. 1C), so this is coded as 1.

224. Axis, intercentrum: present (0)/ absent (1).
An axis intercentrum is present (Fig. 1C) so this is coded as 0.

225. Axis, connectivity to intercentra: to intercentra 2 only (0)/ to intercentra 2 and 3 (1).
The axis is connected to intercentrum 2 only so this is coded as 0.

226. Axis, intercentrum, fusion to axial pleurocentrum: unfused (0)/ fused (1).
From the scan information, the axis intercentrum appears to be fused to the pleurocentrum so we code this as 1.
227. Axis, ribs: present (0)/ absent (1).
Axis ribs are absent (Fig. 1C), so this is coded as 1.

Postaxial vertebrae: Presacral/precloacal pleurocentra
228. Presacral pleurocentra, orientation of centrum: amphicoelous (0)/ procoelous (1)/ opistocoelous (2)/ platycoelous (3)/ amphiplatyan (4).
The presacral pleurocentra are amphicoelous (Figs. 1C, 7N) so this is coded as 0.
229. Presacral pleurocentra, notochord, persistent in adults: present (0)/ absent (1).
The scans show that a cavity is present inside the vertebral pleurocentrum but the ends are solid, so we code this as 1.
230. Presacral parapophyses: present (0)/ absent (1).
We do not have sufficient information so we code this character as ?.
231. Presacral pleurocentra, midventral crest, cervical vertebrae: absent (0)/ present (1)
A distinctive mid ventral crest connected to hypophyses is absent in cervical vertebrae, so this is coded as 0.
232. Presacral pleurocentra, midventral crest, posterior dorsal vertebrae: absent (0)/ present (1)
We do not have sufficient information to decide on presence or absence of a midventral crest on dorsal presacral pleurocentra so this is coded as ?.
233. Posterior presacral pleurocentra, precondylar constriction: absent (0)/ present (1)
This is inapplicable as *Cryptovaranoides* is amphicoelous, so it is coded as (-).
234. Posterior presacral pleurocentra, dorsal vertebrae, margo ventralis (= ventrolateral crest): absent (0)/ present (1).
We do not have sufficient information to decide on presence or absence of a ventrolateral crest on dorsal presacral pleurocentra so this is coded as ?.

Sacral and Caudal vertebrae
235. Sacral vertebrae, number: zero (0)/ one (1)/ two (2)/ three (3)/ four (4).
An isolated sacral vertebra (Fig. 7M) referable to *Cryptovaranoides* is formed of 2 sacral vertebrae, so we code this as 2.
236. Caudal vertebrae, autotomic septum: absent (0)/ present (1).
There are no caudal vertebrae referable to *Cryptovaranoides* in the collection, so this is coded as ?.

Intercentra
237. Intercentra, on cervical vertebrae: absent (0)/ present (1).
Probable intercentra are recognized from the scan below the cervical pleurocentra (Fig. 1C) so this is coded as 1.
238. Intercentra, on cervical vertebrae, position: intervertebral (0)/ on preceding centrum body (1)/ on following centrum body (2).
As the cervical intercentra are displaced (Fig. 1C) we have coded this character as ?.
239. Intercentra, on dorsal vertebrae: absent (0)/ present (1).
No dorsal intercentra are recognized from the scan (Fig. 1D) so this is coded 0.
240. Intercentra, on anteriormost caudal: absent (0)/ present (1).
As caudal intercentra are missing from the collection we have coded this character as ?.

241. Intercentra, on anteriormost caudal, shape: wedge-like elements (0)/ modified into chevron elements (1).
As caudal intercentra are missing from the collection we have coded this character as ?.
242. Intercentra, on posterior caudals (chevron bones): present (0)/ absent (1).
As caudal intercentra are missing from the collection we have coded this character as ?.
243. Chevron bones, articulation: between pleurocentra (0)/ with pleurocentrum, on articulatory facets (1)/ with pleurocentrum, on haemapophyses (=pedicles) (2).
As chevron bones are missing from the collection we have coded this character as ?.
244. Chevron bones, fusion to pleurocentrum: unfused (0)/ fused (1).
As chevron bones are missing from the collection, we have coded this character as ?.
245. Chevron bones, distal fusion: separate elements (0)/ “V” shaped (1) / “Y” shaped (2)/ elliptically shaped (3).
As chevron bones are missing from the collection, we have coded this character as ?.

Neural arches and Neural spines

246. Neural arches, dorsal vertebrae, ventral bridge, posterior borders of neural arches: absent (0)/ present (1).
A ventral bridge on the posterior border of the neural arch of the type found in captorhinids is not present on scanned or isolated dorsal vertebra are absent so we have coded this character as 0.
247. Neural arches, prezygapophyses, presacral vertebrae, processes ventrolaterally on prezygapophyses: absent (0)/ present (1).
Ventrolateral processes on the prezygapophyses of presacral vertebrae are absent so this is coded as 0.
248. Neural arches, presacral vertebrae, zygosphenes: absent (0)/ present (1).
Zygosphenes are present, although rudimentary, on isolated *Cryptovaranoides* presacral vertebrae so this is coded as 1.
249. Neural arches, presacral vertebrae, zygosphenes orientation: facing dorsolaterally (0)/ facing ventrolaterally (1).
From isolated dorsal vertebrae the zygosphene orientation is dorsolateral so we code this character as a 0.
250. Neural arches and pleurocentrum, diapophysis, anterior dorsal vertebrae, fusion to parapophysis: absent (0)/ present (1).
The diapophysis of anterior dorsal vertebrae (Fig. 1 C, D) including isolated bones is fused with the parapophysis so this is coded as 1.
251. Neural arches, margo lateralis, posterior presacral vertebrae: absent (0)/ present (1).
The presence of a margo lateralis on the holotype or on isolated presacral vertebra is uncertain so this is coded as ?.
252. Neural spine, cervical vertebrae, posterior notch: absent (0)/ present (1).
There is insufficient evidence from the holotype or isolated vertebrae to identify a posterior notch of the neural spine on cervical vertebrae so this is coded ?.
253. Neural spine, cervical vertebrae, apically, lateral expansion: absent (0)/ present (1).
A lateral expansion (T shape) of the type found in the neural spine of cervical vertebrae of e.g., archosauriforms, and *Kuehneosaurus* is not found in the holotype or isolated vertebrae so is coded 0.
254. Dermal neural spine, dorsal vertebrae: absent (0)/ present (1).
A dermal neural spine on the dorsal vertebrae is absent in *Cryptovaranoides* so is coded as 0.

255. Neural spine, dorsal vertebrae: present (0)/ absent (1).
A neural spine on the dorsal vertebrae is present in the *Cryptovaranoides* holotype (Fig. 1C, D) and isolated vertebrae so is coded as 0.
256. Neural spine, dorsal vertebrae, anterior midline process: absent (0)/ present (1).
An anterior midline process on the neural spine of dorsal vertebrae is absent in *Cryptovaranoides* so is coded 0.
257. Neural spine, dorsal vertebrae, mammillary process: absent (0)/ present (1).
A mammillary process on the neural spine of dorsal vertebrae (Fig. 1C, D) is absent in *Cryptovaranoides* so is coded 0.
258. Neural spine, dorsal vertebrae, apically, lateral expansion: absent (0)/ present (1).
A lateral expansion (T shape) of the type found in the neural spine of dorsal vertebrae of e.g., *Prolacerta* and *Kuehneosaurus* is not found in the holotype (Fig. 1C, D) or isolated vertebrae so is coded 0.

Presacral ribs
259. Presacral ribs, anteroposterior crests: absent (0)/ present (1).
A T-shaped rib of the type found in the presacral ribs of turtles is not found in the holotype or isolated vertebrae so is coded 0.
260. Presacral ribs, cervical ribs: present (0)/ absent (1).
Some cervical vertebrae e.g., CV 5 bear ribs (Fig. 7N) so this is coded as 0.
261. Presacral ribs, cervical ribs, anterior process: absent (0)/ present (1).
An anterior process on the cervical rib shafts of the type found in e.g., rhynchosaurs and thalattosaurs is not found in the holotype (Fig. 7N) or isolated vertebrae so is coded 0.
262. Presacral ribs, uncinate processes, anterior dorsals: absent (0)/ present (1).
Uncinate processes are absent from the dorsal ribs of *Cryptovaranoides* (Fig. 1C, D) so this is coded as 0.
263. Presacral ribs, anteroventral process at rib head (= pseudotuberculum): absent (0)/ present (1).
Anteroventral processes (pseudotuberculum) are absent from the presacral ribs of *Cryptovaranoides* (Figs. 1C, 7N) so this is coded as 0.
264. Presacral ribs, posterodorsal process at rib head (= pseudotuberculum): absent (0)/ present (1).
Posterodorsal processes (pseudotuberculum) are absent from the presacral ribs of *Cryptovaranoides* (Figs. 1C, 7N) so this is coded as 0.
265. Posteriormost presacral vertebra, ribs: present (0)/ absent (1).
There are no indisputable posteriormost presacral vertebrae in the holotype or as individual isolated bones so the presence or absence of ribs on these elements cannot be assessed and therefore this character is coded as ?.
266. Posteriormost presacral vertebra, ribs articulation: ribs unfused (0)/ ribs fused (1).
As there are no indisputable posteriormost presacral vertebrae in the holotype or as individual isolated bones so the fusion of ribs on these elements cannot be assessed and therefore this character is coded as ?.
267. Sacral ribs, distal forking: absent (0)/ on first sacral rib only (1)/ on first and second sacral ribs (2)/ on second sacral rib only (3).
The sacral ribs on the isolated sacral vertebrae are not forked (Fig. 7M) so this is coded as 0.
268. Sacral/Cloacal ribs, fusion to pleurocentra: unfused (0)/ fused (1).

The sacral ribs on the isolated sacral vertebrae are fused to pleurocentra (Fig. 7M) so this is coded as 1.

269. Anterior caudal ribs: absent (0)/ present (1).
There are no caudal vertebrae or their ribs represented on the holotype or as isolated bones so this character cannot be assessed and is coded as ?.

270. Anterior caudal ribs, fusion to pleurocentra: unfused (0)/ fused (1).
There are no caudal vertebrae or their ribs represented on the holotype or as isolated bones so this character cannot be assessed and is coded as ?.

Sternum

271. Presternum, mineralized: absent (0)/ present (1).
A presternum is not recognized or is absent in the holotype so as we cannot confirm a presence or absence, this character is coded as ?.

272. Xiphisternum: absent (0)/ present (1).
A xiphisternum is not recognized or is absent in the holotype so as we cannot confirm a presence or absence, this character is coded as ?.

Poststernal inscriptional ribs

273. Mineralized poststernal inscriptional ribs: absent (0)/ present (1).
Mineralized poststernal inscriptional ribs (found in e.g., *Sphenodon*) are not recognized or are absent in the holotype so as we cannot confirm a presence or absence, this character is coded as ?.

274. Distal ribs: absent (0)/ present (1).
Distal ribs (found in e.g., *Kuehneosaurus*) are not recognized or are absent in the holotype so, although an absence is likely, as we cannot confirm a presence or absence, this character is coded as ?.

Scapula

275. Scapula, supraglenoid foramen: absent (0)/ present (1).
A supraglenoid foramen is not observed on either scapula in the holotype but this may be due to a lack of resolution in the scan so this is coded as ?.

276. Scapula, supraglenoid buttress: absent (0)/ present (1).
A supraglenoid buttress is present on the scapula (Fig. 1C) so this is coded as 1.

277. Scapula, scapula ray: absent (0)/ present (1).
A scapula ray is not observed on either scapula in the holotype and so this is scored as 0.

278. Scapula, dorsal acromion process: absent (0)/ present (1).
There is an anterodorsal expansion of the dorsal region of the scapula that we suggest is the acromion process, so this is coded as 1.

279. Scapula, supracoracoidal acromion process: absent (0)/ present (1).
An anteroventral supracoracoidal acromion process of the scapula of the type found in turtles is absent and is coded as 0.

280. Scapula, posterior emargination: absent (0)/ present (1).
A posterior emargination of the scapula is absent and is coded as 0.

281. Scapula, anterior emargination: absent (0)/ present (1).
There is a possible emargination on the anterior margin of a scapula but this may be due to breakage post mortem so we code this as ?.

Procoracoid

282. Procoracoid, supracoracoid foramen: absent (0)/ notch (1)/ complete foramen (2).
A large fenestra on the mid region anterior to the glenoid may be a supracoracoid foramen but it is large, so we are uncertain. Therefore, we code this character as ?.

283. Procoracoid, angulation medially: absent (0)/ present (1).
The medial angulation of the procoracoid of the type found in sauropterygians is absent so this is coded as 0**.**

284. Procoracoid, coracoid emargination: absent (0)/ anterior emargination (1).
Although there is a large opening that we interpret as the primary coracoid fenestra (Fig. 7K) and therefore would be coded as 1. The primary fenestra is potentially present in both coracoids, but until we find larger (and therefore adult) bones for confirmation we have decided to score this character as ?

Posterior coracoid

285. Posterior coracoid: absent (0)/ present (1).
A posterior coracoid is absent, so this is coded as 0.

286. Epicoracoids: absent (0)/ present (1).
The likely presence of coracoid fenestra indicate that it is probable that an epicoracoid is present, so we code this character as 1.

Clavicles, Interclavicle and Cleithra

287. Clavicles, secondary curvature anteroposteriorly: absent (0)/ present (1).
From the scans a secondary curvature of the clavicle is observed (Figs. 1C, 7N) so this is coded as 1.

288. Clavicles, proximoventral fenestration: absent (0)/ present (1).
There is no fenestration on the clavicles (Figs. 1C, 7N) so this is coded 0.

289. Clavicles, posterior process: absent (0)/ present (1).
There is no posterior process on the clavicles (Figs. 1C, 7N) so this is coded 0.

290. Clavicles, dorsolateral flange: absent (0)/ present (1).
There is no dorsolateral flange on the clavicles (Figs. 1C, 7N) so this is coded 0.

291. Clavicles, position (at point of contact), in relation to anterior margin of scapula: laterally (0)/ medially (1)/ anteriorly (2).
From the scans the positioning of the clavicle to the scapula appears to be lateral so this is coded as 0.

292. Clavicles, position (at point of contact), in relation to anterior margin of the interclavicle: ventrally (0)/ dorsally (1)/ anteriorly (2).
From the scans there is a clavicle facet on the interclavicle (Fig. 2C) and this indicates that the positioning of the clavicle is ventral, so this is coded as 0.

293. Interclavicle, anterior process: absent (0)/ present (1).
There is no anterior process on the interclavicle (Fig. 1C), so this is coded 0.

294. Interclavicle, posterior process: present (0)/ absent (1).
There is a posterior process on the interclavicle (Fig. 1C), so this is coded 0.

295. Cleithra: present (0)/ absent (1).
There is no cleithrum present, so this is coded 1.

Ilium

296. Ilia, posterodorsal notch, on acetabular margin: absent (0)/ present (1).

The ilia are not preserved in the holotype or as isolated bones, so we code this character as ?.
297. Ilia, supraacetabular buttress: absent (0)/ present (1).
The ilia are not preserved in the holotype or as isolated bones, so we code this character as ?.
298. Ilia, anterior pubic process: absent (0)/ present (1).
The ilia are not preserved in the holotype or as isolated bones, so we code this character as ?.
299. Ilia, anterior (=preacetabular) process: absent (0)/ present (1).
The ilia are not preserved in the holotype or as isolated bones, so we code this character as ?.

Pubis
300. Pubes, obturator foramen: absent (0)/ complete foramen (1)/ notch (2).
The pubis is not preserved in the holotype or as isolated bones, so we code this character as ?.
301. Pubes, pubic tubercle: absent (0)/ present (1).
The pubis is not preserved in the holotype or as isolated bones, so we code this character as ?.

Ischia
302. Ischia, fusion to pubes: absent (0)/ present (1).
An ischium is not preserved in the holotype or isolated, so we code this character as ?.
303. Ischia, anterior border, emargination: absent (0)/ present (1).
An ischium is not preserved in the holotype or isolated, so we code this character as ?.
304. Ischia, ischiadic tuberosity: absent (0)/ present (1).
An ischium is not preserved in the holotype or isolated, so we code this character as ?.
305. Ischia, isquiadic neck: absent (0)/ present (1).
An ischium is not preserved in the holotype or isolated, so we code this character as ?.
306. Ischia, facet, for hypoisquium: absent (0)/ present (1).
An ischium is not preserved in the holotype or isolated, so we code this character as ?.

Humerus
307. Humeri, ectepicondyle foramen: absent (0)/ groove (1)/ notch (2)/ complete foramen (3)
A complete ectepicondylar foramen is present (Fig. 1A, D) so this is coded as 3.
308. Humeri, epicondyles: present (0)/ absent (1).
Epicondyles are present (Fig. 1A, C, D) so this is coded as 0.
309. Humeri, entepicondyle foramen: absent (0)/ opening dorsally only (1)/ opening ventrally only (2)/ fully open ventrally and dorsally (3).
A complete entepicondylar foramen is fully opened dorsally and ventrally (Fig. 1A) so this is coded as 3.
310. Humeri, expanded radial condyle (= capitellum): present (0)/ absent (1).
A capitellum is present (Fig. 1A) so this is coded as 0.
311. Humeri, pectoral process: present (0)/ absent (1).
A deltopectoral crest (Fig. 1A, D), which incorporates a pectoral crest, is present in *Cryptovaranoides* so this is coded as 0 (confirmed as appropriate by Tiago Simões, pers. com).
312. Humeri, pectoral process, connection to humeral head: separate (0)/ connected (1).
A deltopectoral crest (Fig. 1A, D) is present in *Cryptovaranoides* so this is coded as 1.
313. Humeri, shaft angulation: straight (0)/ angulate posteriorly (1).
The shaft of the humerus is straight (Fig. 1D), so this is coded as 0.
314. Humeri, secondary ossification of epiphyses: absent (0)/ present (1).
There does appear to be an epiphysis on the humerus (Fig. 1A) so this is coded 1.

315. Humeri, anterior flange: absent (0)/ present (1).
Cryptovaranoides lacks an anterior flange on the humerus (Fig. 1D) so this is coded 0.

Radius

316. Radia, distal epiphysis, styloid process: absent (0)/ present (1).
There is a possible styloid process is present on the radius of *Cryptovaranoides* (Fig. 7L), but this may be an artefact of scanning and segmentation so we have coded this ?.
317. Radia, anteroproximal process: absent (0)/ present (1).
The radius (Figs. 1C, 7J) lacks an anteroproximal process, so this is coded 0.

Ulna

318. Ulnae, ossified olecranon process: present (0)/ absent (1).
The scan is not sufficiently resolved to decide if an olecranon process is present or absent on the ulna so this is coded ?.
319. Ulnae, distal epiphysis, expansion: absent (0)/ present (1).
The scan is not sufficiently resolved to decide if a ball-like distal epiphysis is present or absent on the ulna so this is coded ?.

Carpals

320. Perforating foramen, manus: absent (0)/ between ulnare and intermedium (1)/ between radiale and intermedium (2).
As the manus has been somewhat displaced (Fig. 7L) it is not possible to tell if a perforating foramen is present between the ulnare and intermedium (or even if an intermedium is present) so this is coded ?.
321. Intermedium: present (0)/ absent (1).
It is not possible to tell if an intermedium is present as the carpus has been slightly displaced (Fig. 7L) so this is coded as ?.
322. Pisiform: absent (0)/ present (1).
Although there is an element that may be the pisiform (Fig. 7L), it is not possible to be sure as the carpus has been slightly displaced so this is coded as ?.
323. Palmar sesamoid: absent (0)/ present (1).
It is not possible to tell if a palmer sesamoid is present as the carpus has been slightly displaced (Fig. 7L) so this is coded as ?.
324. Distal carpal 1: present (0)/ absent (1).
The proximal head of metacarpal 1 has an expanded epiphysis (Fig. 7L) which suggests it is the fused with distal carpal 1 so we tentatively code this as 1.
325. Distal carpal 5: present (0)/ absent (1).
A distal carpal 5 is not preserved in the holotype or as an isolated bone so we code this character as ?.

Femur and Tibia

326. Femora: present (0)/ absent (1).
A femur is not preserved in the holotype or as an isolated bone so we code this character as ?.
327. Femora, internal trochanter: present (0)/ absent (1).
A femur is not preserved in the holotype or as an isolated bone so we code this character as ?.
328. Femora, fourth trochanter: absent (0)/ present (1).
A femur is not preserved in the holotype or as an isolated bone so we code this character as ?.

329. Femora, intertrochanteric fossa: present (0)/ absent (1).
A femur is not preserved in the holotype or as an isolated bone so we code this character as ?.
330. Tibiae, distal epiphysis, notch: absent (0)/ present (1).
A tibia is not preserved in the holotype or as an isolated bone so we code this character as ?.

Tarsals

331. Astragalus and calcaneum: as totally separate elements (0)/ fused (1).
Neither an astragalus nor a calcaneum is preserved in the holotype or as an isolated bone so we code this character as ?.
332. Astragalus, shape, concave laterally: absent (0)/ present (1).
An astragalus is not preserved in the holotype or as an isolated bone so we code this character as ?.
333. Calcaneum, lateral tuber (or process) of the calcaneum: present (0)/ absent (1).
A calcaneum is not preserved in the holotype or as an isolated bone so we code this character as ?.
334. Calcaneum, foramen for perforating artery, position: absent (0)/ between astragalus and calcaneum (1)/ between proximal ends of tibia and fibula (2).
A calcaneum is not preserved in the holotype or as an isolated bone so we code this character as ?.
335. Pedal lateral centrale: absent (0)/ present (1).
A lateral centrale of the pes is not preserved in the holotype or as an isolated bone so we code this character as ?.
336. Distal tarsal 1: present (0)/ absent (1).
A distal tarsal 1 is not preserved in the holotype or as an isolated bone so we code this character as ?.
337. Distal tarsal 2: present (0)/ absent (1).
A distal tarsal 2 is not preserved in the holotype or as an isolated bone so we code this character as ?.
338. Distal tarsal 4, proximal peg: present (0)/ absent (1).
A distal tarsal 4 is not preserved in the holotype or as an isolated bone so we code this character as ?.
339. Distal tarsal 5: present (0)/ absent (1).
A distal tarsal 4 is not preserved in the holotype or as an isolated bone so we code this character as ?.

Metatarsals

340. Metatarsal 5, hooked: absent (0)/ present (1).
A metatarsal 5 is not preserved in the holotype or as an isolated bone so we code this character as ?.
341. Metatarsal 5, plantar tubercle: absent (0)/ present (1).
A metatarsal 5 is not preserved in the holotype or as an isolated bone so we code this character as ?.

Other ossifications

342. Gastralia: absent (0)/ present (1).
The scan indicates that gastralia are absent (Fig. 1D) from the holotype but we cannot be sure that the region is simply missing and not preserved so have coded this as ?..

343. Dorsal trunk osteoderms: absent (0)/ present (1).
There is no sign of dorsal trunk osteoderms on the scan, so these are considered absent (Fig. 1D) and coded as 0.
344. Dorsal trunk osteoderms, imbrication: not imbricated (1)/ imbricated (2).
As there are no dorsal trunk osteoderms present this character is coded as missing (-).
345. Plastron plate: absent (0)/ present (1).
There is no sign of a plastron plate on the scan, so this is considered absent (Fig. 1D) and coded as 0.
346. Neural plates: absent (0)/ present (1).
There is no sign of neural plates on the scan, so this is considered absent (Fig. 1D) and coded as 0.
347. Costal plates: absent (0)/ present (1).
There is no sign of costal plates on the scan, so this is considered absent (Fig. 1D) and coded as 0.
348. Single implantation median tooth at the most mesial part of the premaxilla(e) dentition: median tooth absent, but dentition is continuous on the anterior (mesial) region (0)/ present (1)/ tooth absent in the median position and edentulous in the immediate lateral vicinity (2). Character added. *Modified from Character 413* in (*22*) and originally Lee character 154 (*62*). In *Cryptovaranoides microlanius* a median tooth is present at the most mesial position of the seven teeth of the fused premaxillae of the large specimen (Fig. 4B, C). This is therefore coded as 1. *Priosphenodon* has a median tooth, but this is formed from the fusion of two teeth, one from each premaxilla so it is coded as 0. In unfused premaxilla there is no median tooth in the most mesial position, rather there is a tooth from each premaxilla either side of the median. Median teeth are found throughout the amphisbaenians (and are often enlarged) and are frequently present in lizards, particularly where there is an odd number of teeth on the single premaxilla. Seven premaxillary teeth are typically found in many lizard families (e.g., Iguanians, cordyliforms, lacertoids; other families such as anguids and varanids often have 7–9 (*17*); in fact, 'most lizards have 7–9' Evans p. 258 *(17)*; however median teeth can be present when there are an even number of teeth e.g., in some geckoes. Snakes characteristically are missing a median tooth, but teeth are sometimes present on the more lateral extremities of the premaxillae but not in the immediate vicinity of the median position (such as *Anilius scytale* and *Xenopeltis unicolor* in our matrix). Many snakes, e.g., *Cylindrophis ruffus*, have no teeth on the single premaxilla; in many fossil snakes the snout region is not preserved. In *Placodus gigas and Langobardisaurus pandolfii* this character is treated as missing as the edentulous condition in those taxa is not homologous.

Appendix S3. Rescoring of key taxa in the Simões et al. (*10*) data matrix.

Rescoring of *Megachirella*

In view of the importance of *Megachirella* as perhaps the oldest pan-squamate (named as the oldest squamate by *10*) we referred to the original papers (*65,66*) and the scoring of Simões et al. (*10*). There are a number of differences in these descriptions (*10,65,66*); for example, the earlier authors describe the dentition of *Megachirella* as 'subthecodont' or 'subpleurodont'. Therefore, we contacted Tiago Simões concerning ten of the character descriptions (eight of them focusing on *Megachirella*) and scorings and he kindly responded in detail to the queries. As a consequence, we revised four of the 347 character codings in the matrix (*10*), and we describe the reasons for the amendments below. Additionally, we have reservations about character 123, which we describe below. This amended scoring was conducted before the release of the *(11)* paper and has not been changed following its publication. However, we have accepted their modifications in characters, scoring and constituent taxa and use them separately in the *(11)* matrix in a later section which enables a close comparison between the outcome of the two matrices.

Character 59. Postfrontal medial margin is lateral compared to parietal. Coded as 2 *(10)*, and recoded as ? here—We considered whether this can be confidently scored as 2 because Simões et al. *(10)* state that it is a probable postorbitofrontal, and we are uncertain how the authors can be sure that it is the postfrontal rather than the postorbital that contacts the parietal., As there is no confirmation (e.g., a suture present) that it is the postfrontal that contacts the parietal and rather than pre-empt the relationship with squamates this character is better coded as ?

Character 206. Posterior dentition replacement teeth present. Coded as 1 *(10)*, and recoded as ? here. —We considered whether replacement is actually observed in the posterior dentition as opposed to anterior or mid dentary teeth. As stated, *(10)*, tooth implantation could not be assessed on maxillary teeth. Also a ? was given to character 207*(10)* regarding resorption pits). We decided that the most prudent scoring for this character is ? because the terminal posterior region is the part most likely to have none replaceable teeth in basal rhynchocephalians such as *Gephyrosaurus* (*21*). Therefore, scoring the character as 1 effectively decides a polarity without the likelihood that the most posterior dentition is observed.

Character 211. Absent ankyloses to labial wall in posterior dentary teeth. Coded as 0 *(10),* and recoded ? here. We considered whether ankylosis is actually observed as absent in the posterior dentary teeth as opposed to mid dentary teeth. We also considered if relatively heavy ankylosis was an unobserved possibility at the base or part way up the labial wall. The character in this case is relevant only to ankylosis at the top of the labial wall. However, we consider that the most prudent scoring for this character is ? because the terminal posterior region is the part most likely to have extensive ankylosis that can stretch from the tooth base to the apex of the labial wall in basal rhynchocephalians. That is the condition found in rhynchocephalian taxa from Holwell (*67*) and Hallau (*27*). Therefore, as the most posterior teeth are not certainly observed, we are not confident that this character can be scored 0.

Character 213. Posterior maxillary teeth, labial wall attachment only. Coded as 0 *(10)*, and recoded ? here. We considered whether this can be confidently scored as 0 as Simões et al. *(10)*

state (ESM, p. 6), that 'The exact mode of tooth implantation on the maxilla is hard to verify'. We therefore consider that the most prudent scoring for this character is ? because the implantation on the maxilla cannot be verified, and we would treat this character as independent of the dentary implantation. Also, as mentioned above we cannot be sure that the dentary dentition is the most posterior.

Character 123. Notch on quadrate for squamosal present. Coded as 1 *(10)*, and coded as 1 here also. We considered whether this can be confidently scored as 1 because Simões et al. *(10)* state in their descriptor for character 51 that the squamosal 'inserts into a lateral notch on the quadrate in most squamates' but we note that the character state is more general here. It could be that such a notch is seen as a precursor of that found in most squamate quadrates and usually not recorded in other reptiles. However, we suggest that this character is too general and effectively links *Megachirella* to squamates without the specific morphology of the squamate squamosal/ quadrate connection. There is, for example, a notch for the squamosal on the quadrate of *Sphenodon* (*68*, fig. 67.3) but the structure is coded as 0 for the taxon *(10)*. In this case, because the character state is general, we have left the coding for *Megachirella* as 1 *(10)*.

Rescoring of *Marmoretta*

In view of the importance of *Marmoretta* in the Simões phylogeny *(10)*, we referred to the original descriptions (*69,70)* which report associated skull and postcranial material, and the more recent scoring of the taxon (*7,11*). As a consequence, we have revised 16 of the 347 codes in the matrix of *(10),* and we describe the amendments below.

20. Maxillae, premaxillary process: present (0)/ absent (1).
A premaxillary process is present on the maxilla (*69,70)*, so this is re-coded as 0.
85. Parietals, parietal table, shape: margins ventrally directed, sagittal crest present (0)/ margins ventrally directed, without sagittal crest (1)/ margins laterally directed (2).
A sagittal crest is present on the parietal (*69,70)*, so this is re-coded as 0.
97. Palatines, teeth: absent (0)/ present (1).
Teeth are present on the palatines (*69*), so this is re-coded as 1.
105. Pterygoids, teeth: absent (0)/ present (1).
Pterygoid teeth are present (*69*), so this is re-coded 1.
113. Ectopterygoids: absent (0)/ present (1).
Ectopterygoids are present (*69*), so this is coded as 1.
115. Epipterygoid: present (0)/ absent (1).
An epipterygoid is present (*69*), so this is re-coded as 0.
121. Quadrates, quadrate conch: absent (0)/ present (1).
A quadrate conch is absent (*69*), so this is re-coded as 0.
130. Basioccipital/basisphenoid, sphenoid tubercles: absent (0)/ present (1).
Sphenoid tubercles are present on the basioccipital (*69*), so this is coded as 1.
132. Basioccipital, fusion, to exoccipital: unfused (0)/ fused (1).
The basioccipital and exoccipital are separate (*69*), so this is re-coded as 0.
143. Basisphenoid, Vidian canal: open (0)/ fully enclosed (1).
The vidian canal is open within the basisphenoid (*69*), so this is re-coded as 0.
136. Basisphenoid, basipterygoid processes: present (0)/ absent (1).
Basipterygoid processes are present on the basisphenoid (*69*), so this is re-coded as 0.

147. Parasphenoid, teeth: absent (0)/ present (1).
Teeth are absent on the parasphenoid (*69*), so this is re-coded as 0.
165. Dentaries, symphyses, fusion to each other: unfused (0)/ fused (1).
The dentaries are unfused at the symphysis (*69*), so this is re-coded as 0.
167. Dentaries, anterior end, split by Meckelian canal: absent (0)/ present (1).
The symphysial area is split by the Meckelian canal (*69*), so this is coded as 1.
168. Dentaries, anterior end, symphysial articulatory facet, position: on dorsal margin only (0)/ on dorsal and ventral margins (1)/ on ventral margin only (2).
The articulatory facet is positioned on both dorsal and ventral symphysial areas (*69*), so this is coded as 1.
190. Articulars, retroarticular process: absent (0)/ present (1).
A retroarticular process is uncertain (*7,69*), so this is coded as ?. (but *(11)* show this should be 0).

Rescoring of five taxa, inserted into the data matrix of Simões *(10)* with addition of presence of median tooth in premaxilla as character 348; scoring for character 348.

Cryptovaranoides_microlanius
(01)0000011?0???0011000??????0000010?00-1----
00??1?0011100??????10???00101?????????????????011101???1001???01011010?110?1111101?
01?011?00?101?0????0??0??-
1110???????11100**0**10001000100?0211??00??11010?0110?0000010000000000000010111001101?
0?-
?2?1?0??????00101??000000000000??01???????1?100??010**?**100000001???????????303001010
?0??????1???????????????????0-0001

Megachirella_wachtleri ?????????? ??????10?? ?????????? ?0?0010010 0101- -1000 111000--?-
-??11-1110 ?101?00?0- ??0-10???? ?????????? ???0101100 ?010??1101 111??????1 ???200?00?
?1????00?0 0??0?????? ????????00 0100?10??? ?00?100?01 1010?01001 00000???00
?0?0??0??? ??????0010 ?1-???10?? ?????000-? 0000000000 0000?????? ?000????00
0?000?1?00 ????1????? ??????3030 0101000000 0?01?????? ?????????? ?10-000?

Marmoretta_oxoniensis
0000000?0???????1000 ????????0??0??010??? ??0???1??? ?????0??21 ??????1?10 01????0???
????0????? ??????1??? ????1????? ??1?0????? 0????????1 ?0??00???? ??????0??? ??????????
????0?1100 0100010??0 200??00??? --0-001000 ?000010000 00000000?? ???????010 ?1-
??????? ?????000-1 ???00?00? ?????????? ?????????? ?????????? ?????????? ??????????
?????????? ?????????? ?????????? ????0000

Vellbergia bartholomaei
???????????????1000?01?00??0??00?010?????0011100??????000?10?????01100??1?00??????
????????????????????????????????????0???00??00
0100?10??0?0???00??1???????????0000???02000000?0????????????????????????????????????
???
?????

Taytalura alcoberi
0?0000??????10?1000??10?01-??-3000100100?001110011110?0001100-11-
011000?10300????0-
110?????????1100??101?1100?011001?01100????????????01?????????000?????110???????001
1000100?100302111100??1??0-
?010???00001000003300000??
??0

Scoring for character 348 based on data in *(10,11,16,17)***:** *Protorothyris archeri* 0; *Protocaptorhinus pricei* 0*; Captorhinus aguti* 0; *Labidosaurus hamatus* 0; *Eunotosaurus africanus* ?*; Proganochelys queensdedti* -; *Odontochelys semitestacea* ?; *Kayentachelys aprix* 0; *Petrolacosaurus kansensis* 0*; Araeoscelis gracilis* ?; *Araeoscelis casei* 0; *Claudiosaurus germaini* 0*; Youngina capensis* 0; *Hovasaurus boulei* ?; *Acerosodontosaurus piveteaui* ?; *Saurosternon bainii* ?; *Coelurosauravus jaekeli* 0; *Coelurosauravus elivensis* ?; *Hupehsuchus nanchangensis* 0; *Parvinatator wapitiensis* 0; *Gulosaurus helmi* 0; *Utatsusaurus hataii* 0; *Mixosaurus panxianensis* 0; *Prolacerta broomi* 0; *Protorosaurus speneri* ?; *Tanystropheus longobardicus* 0; *Langobardisaurus pandolfii* -; *Megalancosaurus preonensis* ?; *Macrocnemus fuyuanensis* ?; *Macrocnemus bassanii* ?; *Endennasaurus acutirostris* 0; *Askeptosaurus italicus* ?; *Xinpusaurus kohi* 0; *Philydrosaurus proseilus* 0; *Champsosaurus lindoei* 0; *Trilophosaurus buettneri* 0; *Mesosuchus browni* 0; *Howesia browni* ?; *Teyumbaita sulcognathus* 0; *Hyperodapedon huenei* 0; *Proterosuchus alexanderi* 0; *Proterosuchus fergusi* 0; *Euparkeria capensis* 0; *Erythrosuchus africanus* 0; *Largocephalosaurus qianensis* 0; *Sinosaurosphargis yunguiensis* 0; *Cyamodus hildegardis* 0; *Placodus gigas* -; *Wumengosaurus delicatomandibularis* ?; *Serpianosaurus mirigioliensis* 0; *Lariosaurus calcagnii* 0; *Pistosaurus longaevus* 0; *Palaeagama vielhaueri* 0; *Kuehneosaurus latus* 0; *Icarosaurus siefkeri* 0; *Pamelina polonica* 0; *Megachirella wachtleri* ?; *Marmoretta oxoniensis* 0; *Paliguana whitei* ?; *Sophineta cracoviensis* 0; *Gephyrosaurus bridensis* 0; *Diphydontosaurus avonis* 0; *Planocephalosaurus robinsonae* 0; *Homeosaurus maximiliani* 0; *Clevosaurus hudsoni* 0; *Palaeopleurosaurus posidoniae* 0; *Kallimodon pulchellus* 0; *Priosphenodon avelasi* 0; *Sphenodon punctatus* 0; *Trioceros jacksonii* ?; *Uromastyx aegyptia* 1; *Priscagama gobiensis* 1; *Pleurodontagama aenigmatoides* ?; *Igua minuta* ?; *Polrussia mongoliensis* ?; *Oplurus cyclurus* ?; *Stenocercus scapularis* 0; *Hoplocercus spinosus* ?; *Pristidactylus scapulatus* 1; *Iguana iguana* 1; *Polychrus marmoratus* 1; *Crotaphytus collaris* 1; *Liolaemus signifier* 0; *Leiocephalus carinatus* 1; *Phrynosoma modestum* 1; *Plestiodon fasciatus* 1; *Mabuya mabouya* ?; *Slavoia darevskii* 1; *Globaura venusta* 0; *Cordylus niger* 1; *Broadleysaurus major* 1; *Timon Lepidus* 1; *Lacerta viridis* 1; *Meyasaurus diazromerali* ?; *Paramacellodus oweni* ?; *Gobinatus arenosus* ?; *Gilmoreteius chulsanensis* ?; *Teius teyou* 0; *Petracola ventrimaculatus* ?; *Xantusia vigilis* 1; *Tepexisaurus tepexii* ?; *Ardeosaurus brevipes* 0; *Eichstaettisaurus schroederi* 0; *Gobekko cretacicus* 0; *Dactylocnemis pacificus* ?; *Gekko gecko* 1; *Coleonyx variegatus* 1; *Huehuecuetzpalli mixtecus* 0; *Dalinghosaurus longidigitus* ?; *Xenosaurus grandis* 1; *Pseudopus apodus* 1; *Elgaria multicarinata* 0; *Heloderma suspectum* 1; *Lanthanotus borneensis* 0; *Varanus salvator* 1; *Aigialosaurus* ?; *Adriosaurus suessi* ?; *Pontosaurus* 2; *Najash rionegrina* ?; *Dinilysia patagonica* ?; *Pachyrhachis problematicus* ?; *Cylindrophis ruffus* 2; *Xenopeltis unicolor* 2; *Anilius scytale* 2; *Spathorhynchus fossorium* 1; *Rhineura floridana* 1; *Bipes biporus* 1; *Blanus cinereus* 1; *Dibamus novaeguineae* 1; *Vellbergia bartholomaei* ?; *Cryptovaranoides microlanius* 1; *Taytalura alcoberi* 0.

Appendix S4. Character list from Griffiths *et al.* *(11)*

New matrix of Griffiths *et al. (11)*
In the time following reviews and the authors receiving the reports and pre-edit, a number of important papers were published. In particular, *(11)* added 32 new characters and modified the Simões et al. *(10)* matrix. Their modifications especially rescored the 347 characters used by *(10)* and they moved Simões character 52 to 51 (and removed the original Ch 51 of *10*) as well as made new characters 52 and 55; they further added new characters numbered 348-387. We outline the new characters below; for derivation of the characters see Griffiths et al. *(11)*. Griffiths et al. removed many taxa: *Protorothyris*, *Protocaptorhinus*, *Captorhinus*, *Labidosaurus*, *Araeoscelis gracilis*, *Araeoscelis pricei*, *Hovasaurus*, *Saurosternon*, *Parvinatator*, *Gulosaurus*, *Mixosaurus*, *Endennasaurus*, *Xinpusaurus, Kayentachelys*, *Erythrosuchus, Homeosaurus*. *Kallimodon* and *Macrocnemus bessanii.* They added *Fraxinisaura rozynekae, Chaohusaurus chaoxianensis*, *Cartorhynchus lenticarpus* and *Vallesaurus cenensis* We have checked the taxa in the new matrix from the literature and we detail our scoring for *Cryptovaranoides microlanius*. The addition of *Cryptovaranoides* makes a total of 116 operational taxonomic units using this matrix.

The new characters and the scoring of *Cryptovaranoides microlanius* are given below.

52. Squamosal, dorsal process, contact with supratemporal or supratemporal process of parietal: sutured (0) / not sutured, forms a buttress for posterolateral corner of the skull roof (1)
The squamosal of *Cryptovaranoidies* is of the squamatan long rod type (Fig. S1A) and it is likely that there is no sutural contact and streptostyly is likely, but as we do not have any certain parietal we cannot be certain of the relationship with other bones so we score this ?

55. Squamosal, anterior process, dorsoventral length: narrow, < one-quarter of the dorsoventral length of the postorbital region (0) / deep, > one-third of the dorsoventral length of the postorbital region (1).
The *Cryptovaranoides* squamosal is of the squamatan (Fig. S1A), rather than rhynchocephalian, type and is scored here as 0.

348. Skull proportions: preorbital skull length equal to postorbital length (0) / preorbital length exceeds postorbital skull length (1) / postorbital length exceeds preorbital skull length (2).
As we do not have any parietal to base our scoring for this character it is difficult to be sure but our *Cryptovaranoides* reconstructions (Fig. 2A, E, S1A, D) generally suggest that the score should be 1 which we tentatively give here. Although *(11)* note that 'in derived rhynchocephalians and many squamates the postorbital region is longer than the preorbital (state 2), or both regions are of similar length (state 0),' that is not the case in the squamate *Varanus salvator* which is clearly state 1 and is scored as such by those authors.

349. Prefrontal/palatine antorbital contact: absent (0) / narrow forming less than 1/3 the transverse distance between the orbits (1) / contact broad, forming at least 1/2 the distance between the orbits (2).
From isolated unregistered palatines we can identify the prefrontal facet and score *Cryptovaranoides* as 2.

350. Postfrontal contribution to upper temporal fenestra: postfrontal excluded (0) / postfrontal included (1).
We have not recognised a postfrontal in the collection and therefore score this character as ?

351. Angular lateral exposure: exposed along 1/3 of the lateral face of the mandible (0) / exposed only as a small sliver along the lateral face (1).
The angular is only exposed as a small sliver in *Cryptovaranoides* (Fig. 2 B, S1B*)* so we score this 1.

352. Maxilla orbital exposure: absent (0) / present (1).
Scrutiny of isolated maxillae suggests that there is a small area of orbital exposure in *Cryptovaranoides* (Fig. S1A) so we score this 1.

353. Maxilla length: extends at least to the posterior orbital margin (0) / does not reach posterior margin of orbit (1).
The maxilla does not reach the posterior margin of the orbit in *Cryptovaranoides* (Fig. S1A) so this is scored as 1.

354. Frontal, morphology: parallelogram shaped (0) / hour-glass shaped (1).
The frontal morphology of *Cryptovaranoides* is best described as parallelogram-shaped (Fig. S1 D) when viewed dorsally (state 0).

355. Dorsal vertebrae, transverse processes: short no more than the total transverse width of the neural arch (0) / long exceeding the transverse width of the neural arch (1).
Transverse processes are short on the dorsal vertebrae of *Cryptovaranoides* (Fig. 1 C, D*)* so this is scored as 0.

356. Pterygoid, orientation of transverse flange: directed predominantly laterally or posterolaterally (0) / oriented in an anterolateral direction (1)
From the CT scan we conclude that *Cryptovaranoides* has an anterolaterally directed transverse flange (state 1).

357. Humerus, torsion: proximal and distal end are off-set at an angle of at least 45°(0) / off set is reduced to no more than 20° (1).
It is clear from the holotype (Fig. 1 D) and isolated specimens that the torsion of the humerus in *Cryptovaranoides* is about 45° so this is scored as 0.

358. Mandible, adductor chamber: small, quadrate does not extend well below level of occipital condyle (0) / enlarged adductor chamber, and quadrate extends well below occipital condyle (1).
The holotype of *Cryptovaranoides* is a juvenile so the adductor chamber relative size is difficult to judge but from the quadrate present and isolated quadrates (Fig. 6D-F) it is clear that the quadrate extends below the occipital condyle, so this is scored as 1.

359. Metacarpals, length of third and fourth: fourth metacarpal longer than third (0) / third and fourth metacarpals subequal in length (1) / fourth metacarpal shorter than third (2) (from Gauthier *et al.* 1988 *(30)* ch.105)
In *Cryptovaranoides* metacarpal IV is subequal to metacarpal III (Fig. 7L; state 1).

360. Lacrimal, size: large, with an anterior or posterior (suborbital) process which is longer anteroposteriorly than the dorsoventral length of the lacrimal in lateral view (0): small, dorsoventral length greater than anteroposterior length and confined to the orbital rim (1).
We score *Cryptovaranoides* 1 as it is for squamates. The lacrimal in the taxon is of similar relative size (about 8% of the antorbital region; Fig. S1A) to *Varanus salvator*; also, in *Xenosaurus grandis,* the lacrimal is anteroposteriorly longer than the dorsoventral length (see *(17,* fig. 1.83 and *16*) but this is scored as 1 by *(11)* which we have left unchanged,

361. Fibula, articulation with femur: fibula/femur articulation end to end (0) / fibula sits in a recess on lateral margin of distal end of femur (1) (modified from Gauthier *et al.* 1988 *(30)* ch.124)
There is no fibula recognised in the *Cryptovaranoides* collection so this is scored as ?

362. Palatine, lateral row of enlarged teeth: absent (0) / lateral tooth row present on palatine, converging posteriorly (1)
A lateral row of enlarged teeth is absent in *Cryptovaranoides (*Fig. 1 D, S1E) so this is scored as 0.

363. Dentary, posterior extent: extends posteriorly no further than the level of coronoid eminence or slightly beyond (0) / extends posteriorly more than halfway between coronoid eminence and articular condyle (1).
The posterior extent of the dentary in *Cryptovaranoides* is about the level of the coronoid eminence (Fig. S1B) so this is scored as 0.

364. Ectopterygoid, contact with maxilla: absent (0) / present (1)
There is an ectopterygoid facet on the maxilla of *Cryptovaranoides* (Fig. 3 C) so this is scored as 1.

365. Vertebrae, cervical and/or dorsal, anterior centrodiapophyseal or paradiapophyseal lamina: absent (0) / present (1).
This lamina is absent in *Cryptovaranoides* (Fig. 7N) so is scored as 0.

366. Vertebrae, posterior cervical and/or dorsal, prezygodiapophyseal lamina: absent (0) / present (1).
This lamina is absent in *Cryptovaranoides* (Fig. 7N) so is scored as 0.

367. Jugal, anterior suborbital extension: broadly separated from prefrontal or posterior to the midpoint of the orbit (0) / reaches level of prefrontal or anterior margin of orbit (1).
The jugal reaches the anterior margin of the orbit in *Cryptovaranoides* (Fig. 2A) so is scored as 1.

368. Basioccipital, articular surface of the occipital condyle: concave (0) / hemispherical (1) The articular surface of the *Cryptovaranoides* is hemispherical so this is scored as 1.

369. Jugal, lateral exposure below orbit: absent (0) / partly exposed above orbital margin of maxilla (1) /entirely exposed above orbital margin of maxilla (2).

We score his 2 for *Cryptovaranoides* as the jugal appears to be well exposed above the orbital margin of the maxilla (Fig. 2 A).

370. Manus, penultimate phalanges: shorter than or equal to antepenultimate (0) / longer than antepenultimate (1).
The manus is not sufficiently preserved in the *Cryptovaranoides* fossils for this to be scored other than ?

371. Jugal, posteroventral process: short or spur-like, anteroventrally less than 20% of the total ventral length (0) / long, greater than 25% of the total ventral length (1) .
The jugal posteroventral process is absent in *Cryptovaranoidses* (Fig. S1 A) so this is scored as -.

372. Jugal, contact with quadratojugal: absent (0) / present (1).
The jugal has no contact with a quadratojugal (Fig. S1A) so this is scored as 0.

373. Vomers, contact with anterior region of maxilla: absent (0) / present (1).
In view of the scorings of *(11)* where contact between the vomer and anterior region of the maxilla is deemed to be 0 in neochoanate squamatans (e.g., *Mabuya, Petracola, Varanus* and *Dibamus*) we follow suit with *Cryptovaranoides* and score as 0.

374. Prefrontal, contact with counterpart at midline: absent (0) / present (1).
From the CT scan we are confident that contact between the prefrontals is absent in *Cryptovaranoides* (Fig. S1 D) and is scored as 0.

375. Interclavicle, notch on anterior margin: absent (0) / present (1).
From the CT scan of the *Cryptovaranoidse* holotype (Fig. 1C) there is no notch on the anterior margin of the interclavicle so this is scored as 0.

376. Tibia, contact with centrale: absent (0) / present (1).
No tibiae are referable to *Cryptovaranoides* so this is scored as ?

377. Premaxilla, contact with prefrontal: absent (0) / present (1).
There is no contact between premaxilla and prefrontal in *Cryptovaranoide*s (Fig. S1 D; state 0).

Griffiths *et al. (11)* also modified characters: 23, 41, 61,108, 116, 119, 122 and 309. All these are scored for *Cryptovaranoides* the same as for the Simões matrix *(10)* above except character 309 which is scored 1 as an entepicondyle foramen is present in the humerus.

We added character 378 which is the character 348 we added to *(10)* where we give more details. This character is: Single implantation median tooth at the most mesial part of the premaxilla(e) dentition: median tooth absent, but dentition is continuous on the anterior (mesial) region (0)/ present (1)/ tooth absent in the median position and edentulous in the immediate lateral vicinity (2). As in the Simões et al. *(10)* matrix, in *Placodus gigas* and *Langobardisaurus pandolfii* this character is treated as missing as the edentulous condition in those taxa is not homologous. Taxon scorings for character 378 are the same as for the character 348 in the *(10)* matrix above except for four new taxa scored as: *Chaohusaurus chaoxianensis* ?; *Cartorhynchus lenticarpus* ?; *Fraxinisaura rozynekae* ?; *Vallesaurus cenensis* ?.

Scoring of *Cryptovaranoides microlanius* for Griffiths et al. (11) matrix.

Cryptovaranoides_microlanius (01)0000011?0???0011000??????0000010?00-1----
00??1?001?100??????10???00101????????????????011101???1001???01011010?110?1?111010
01?011?000001201???0??0?0-
1110???????111001100010001 00?0211??00??11010?0110?0000010000000000001011100 1101?
0?-
??2?1?0??????00101??0000000000000??01???????10100??010**?**100000001???????????301001010
?0??????1??????????????????0-00012?1?10010111?00100112?00000?01

Appendix S5. Character list from Martinez *et al.* *(32)*. The current state-of-play with regard to phylogenetic analysis of Squamata is far from satisfactory, and we have to work within this problematic regime. The key problem is that the current morphological data matrices (*7,9,10,11,17,22,30,36,59*) do not recover the generally agreed molecular tree (*6,8,34,44,45*) and much work by the scientific community will be necessary to resolve this.

We conducted a combined molecular-morphological Bayesian phylogenetic analysis as the best current means to cut through the deeper problems. We explored all the current data matrices, and these highlighted the issues to be resolved more widely, namely that all published morphological data matrices, right up to 2022, do not generate a phylogeny that approaches the agreed molecular phylogeny. We also found that running parsimony analyses constrained to the molecular tree did not resolve the issue – the main finding was that the modern taxa fit the constraint, but most of the fossil lizards then clustered in a single clade, separate from the modern taxa.

Our fourth phylogenetic analysis is based on the most widely used and current comprehensive molecular-morphological data matrix, from Martínez et al. (*32*), and representing an evolving and corrected matrix from earlier versions in a series of papers e.g. (*10*). We added *Cryptovaranoides*, to the matrix following their analytical parameters and using MrBayes, and this gives an unequivocal result where the new taxon is identified as an anguimorph, and in the frame of the current molecular phylogenetic tree of Squamata Fig. 8.

Scoring of *Cryptovaranoides microlanius* for Martinez et al. (*32*) matrix.

Cryptovaranoides_microlanius
(01)0000011?0???0011000??????0000010?00-1----
00??1?0011100??????10???00101????????????????011101???1001???01011010?110?1111101?
01?011?00?101?0????0??0??-
1110???????11100**0**10001000100?0211??00??11010?0110?00000100000000000001011100 1101?
0?-
??2?1?0??????00101??0000000000000??01???????1?100??010**?**100000001???????????303001010
?0??????1??????????????????0-00000

Appendix S6. List of 78 taxa used in the dated timetree (Fig. 9). For each taxon, we give name, geological formation, stratigraphic age, and the age range, showing first appearance datum (FAD) and last appearance datum (LAD). Figure 9 adheres to the widely accepted molecular phylogenetic tree. The phylogeny derives from the molecular phylogeny of Figure 8, relating to published papers by Simoes et al. (*10*), Martinez et al. (*32*), but with inclusion of all squamate families, based primarily on Burbrink et al. (*34*), as explained in the figure caption and in more detail in the Supplementary Methods.

**Taxon**	**Formation**	**Stage**	**FAD**	**LAD**
Petrolacosaurus_kansensis	Stanton	Kasimovian	307	303.7
Youngina_capensis	Daptocephalus	Lopingian	259.1	251.9
Saurosternon_bainii	Daptocephalus	Lopingian	259.1	251.9
Paliguana_whitei	Daptocephalus/ Lystrosaurus	Lopingian-Induan	259.1	251.2
Fraxinasaura_rozynekae	Untere Graue Mergel	Ladinian	240	238
Kuehneosaurus_latus	Cromhall-Lockatong	Rhaetian	208.5	201.3
Marmoretta_oxoniensis	Kirtlington	Bathonian	168.3	166.1
Megachirella_wachtleri	Dont	Anisian	247.2	242
Sophineta_cracoviensis	Czatkowice	Olenekian	251.2	247.2
Gephyrosaurus_bridensis	Pontalun-Pant	HET-SIN	201.3	190.8
Diphydontosaurus_avonis	Tytherington	Rhaetian	208.5	201.3
Vellberg_jaw	Untere Graue Mergel	Ladinian	240	238
Whitakersaurus_bermani	Ghost Ranch/ Apachean	Rhaetian	208.5	201.3
Rebbanasaurus_jaini	Kota	Toarcian	182.7	174.1
Clevosaurus_hudsoni	Cromhall	Rhaetian	208.5	201.3
Palaeopleurosaurus_posidoniae	Posidonienschiefer	Toarcian	182.7	174.1
Pleurosaurus_goldfussi	Solnhofen Plattenkalk	KIM-TTH	157.3	145
Kallimodon_pulchellus	Solnhofen Plattenkalk	KIM-TTH	157.3	145
Priosphenodon_avelasi	Candeleros	CEN	100.5	93.9
Sphenodon_punctatus	subfossil/ Recent	Holocene	0.01	0
Planocephalosaurus_robinsonae	Cromhall-Tytherington	Rhaetian	208.5	201.3
Huehuecuetzpalli_mixtecus	Tlayúa		105	100.5
Eichstaettisaurus_schroederi	Solnhofen Plattenkalk	KIM-TTH	157.3	145
Dibamidae	TGL-A/2, biozone A	E. Oligocene	33.9	0
Eublepharidae	Brooksville 2	L. Oligocene	27.8	0
Diplodactylidae	subfossil		0.01	0
Carphodactylidae	subfossil		0.01	0
Gobekko_cretacicus	Djadokhta	CMP-eMAA	75	71
Pygopodidae	Riversleigh		22	0
Gekkonidae	Jbel Guersif	THA	59.2	0

Sphaerodactylidae	MN2	eMIO	22.4	0
Ardeosaurus_brevipes	Solnhofen Plattenkalk	KIM-TTH	157.3	145
Paramacellodus_oweni	Kilmaluag; Purbeck	BTH-BER	168.3	139.8
Scincidae	Perriére	PRB	37.7	0
Gerrhosauridae		Mio	20.4	0
Cordylidae	Maevarano	eMAA	72.1	0
Xantusiidae	Aguja	lCMP	80	0
Bipedidae	Willwood (Wa-0)		55.8	0
Trogonophidae	karst fills; MP28	Olig	28.4	0
Amphisbaenidae	Bayan Mandahu	CMP-eMAA	75	0
Lacertidae	El Castellar	uPaleocene	59.2	0
Gymnophthalmidae			0	0
Teiidae	La Pedrera da Rúbies	eBRM	129.4	0
Cryptovaranoides_microlanius	Cromhall	Rhaetian	208.5	201.3
Chamaeleonidae	Dolnice	eMio	20.4	0
Agamidae	Hukawng amber	eCEN	99.6	0
Phrynosomatidae	Florida	eMio	23	0
Iguanidae	Rio Negro	eCEN	100.5	0
Dactyloidae	Brooksville 2	lOlig	27.8	0
Tropiduridae	Cypress Hills	lEoc	37.2	0
Leiocephalidae	Cedar Ridge	Olig	33.9	0
Corytophanidae	Lucky lizard locality	Ypr	56	0
Crotaphytidae	Split Rock	mMio	18	0
Liolaemidae	Paso del Río Arriba Cliffs	Pli	5.3	0
Hoplocercidae	Djadokhta	mCMP	80	0
Opluridae	Seychelles	Pleist	2.58	0
Leiosauridae			0	0
Polychrotidae	Green River	eEOC	51.4	0
Helodermatidae	Barun Goyot	eMAA	72.1	0
Diploglossidae			0	0
Anniellidae	Bolinger Canyon Quarry	mMio	13	0
Anguidae	Smoky Hollow M.	TUR	92	0
Xenosauridae	Ilek	APT-ALB	125	0
Shinisauridae	Yixian	BRM-APT	129.7	122.1
Mosasauroidea	Akrabou	mTUR	94.3	89.3
Varanidae	Djadokhta	CMP-eMAA	75	0
Eophis_underwoodi	Forest Marble	BTH	168.3	166.1
Najash_rionegrina	Candelaros	CEN-TUR	100.5	89.8

Leptotyphlopidae	Ano Metochi	lMio (MN13)	5.4	0
Typhlopoidea	Adamantina	TUR-CMP	87.8	0
Anomalepididae	Tornillo		65	0
Haasiophis_terrasanctus	Ein Yabrud quarries	eCEN	100.5	93.9
Amerophidia	Allen	lCMP-eMAA	72.1	0
Pythonoidea	Medicine Pole Hills		35.2	0
Booidea	Cerrejón		58	0
Uropeltoidea			0	0
Caenophidia	Wadi Milk	CEN	72.1	0

Appendix S7. Summary of *Cryptovaranoides* apomorphy distributions across major clades and key taxa. Colors: green (Appendix S7A) are those considered 'Squamate' apomorphs by (*22*) whilst those in blue (Appendix S7B) are in a squamate+ *Huehuecuetzpalli* clade of *(22)*. Light gold-colored characters (Appendix 7B) are those that are apomorphies *within* the clade Squamata, so are Unidentatan or Anguimorph characters. Abbreviations: G12, Gauthier et al. (*22*); G88, Gauthier et al. (*30*); GQ20, Gauthier et al. (*12*); QG20, De Queiroz et al. (*13*); S18, Simões et al. (*10*).

Description: The apomorphies are listed so that those that are autapomorphies (character number 1) or have few exceptions have lower numbers. To keep the table as simple but as useful as possible the scoring for characters is given as follows: ✓=present: ✓=present but only in some taxa; X=generally absent; x=known to be absent in some taxa; x=absent in a few taxa or specimens; -- = character missing; ? =no evidence. Most of the exceptions to the synapomorphs are in characters 2-4 and 7; these are explained in the text. The common presence or absence of the synapomorphy in the clade is recorded for the other characters 5-38 except where there are notable variations e.g., some geckoes have a rugose dorsum but the large majority do not. For character numbers 5-38 we are scoring where the *Cryptovaranoides* apomorphy is prevalent in the clades Squamata, Gekkonomorpha, Unidentata and Anguimorpha. Where bones are typically missing e.g., the jugal in many geckoes is minute or absent then any character involving the bone is scored as ---.

Apomorphy key: 1=Humerus without entepicondylar foramen, G88SCh98 ; 2=All ribs are unicapitate, QG20; 3=Anterior ramus of the pterygoid does not contact the vomer, QG20; 4=Vertebrae procoelous, G12Ch467; 5= Premaxillae fused postnatally, G12Ch1; 6=Presence of Median premaxillary tooth, G12Ch413; 7='Rod-shaped' squamosal with peg for notch or socket in head of quadrate, G12Ch177 ; 8=vomer and maxilla meet at anterior margin of fenestra exochoanalis, G12Ch113 ; 9=Choanal fold on the palatine, G12Ch250; 10=Metotic fissure subdivided by crista tuberalis, S18Ch153, G12Ch354; 11= Vidian canal enclosed within sphenoid as it passes across the basipterygoid process, G12Ch336 ; 12=Quadrate head with lateral notch for squamosal, QG20; 13=Quadrate foramen absent or tiny, G12Ch187; 14= medially positioned posterior myohyoidal foramen on the lower jaw, G12Ch384; 15=fusion of exoccipitals and opisthotics to form an otoccipital, G12Ch352; 16= The fronto-parietal joint is kinetic (inferred), QG20; 17=anteromedial process of the coronoid fits into a sulcus beneath tooth-bearing border of dentary, G12Ch388; 18=Septomaxilla medially positioned, forming part of the nasal cavity and roofing the vomeronasal organ, G12Ch199; 19=frontal underlaps (or barely overlaps) parietal laterally on frontoparietal suture, G12Ch58; 20= palatine extends posteriorly so pterygoid enters sub orbital fenestra, G12Ch240; 21=Trunk intercentra absent, 22= Angular does not extend posteriorly to articular condyle, G12Ch381; QG20; 23=coronoid eminence formed by coronoid bone only, not in the combined process of the dentary and prominent dorsal expansion of the surangular, G12Ch387; 24=Quadratojugal not present as a separate element,G12Ch181; 25= short overlap in quadrate-pterygoid contact, G12Ch182; 26=scapulocoracoid emargination/fenestration present, G12Ch493; 27=jugal posterior process absent, G12Ch155; 28= coronoid postero medial process present, G12Ch393; 29=jugal closely approaches level of prefrontal below orbit, GQ20Ch2 ; 30=jugal entirely exposed above labial margin of maxilla, GQ20Ch3; 31= coronoid reaches lateral side of surangular, GQ12Ch12;

32=frontoparietal suture moderately interdigitated, G12Ch56; 33=rugose ornamentation over dorsum, G12Ch572; 34=jugal lies ventral to lacrimal, G12Ch145; 35=posterodorsal trending ridge delineates anterior limits of naso-lacrimal fossa, G12Ch117; 36=Septomaxilla contacts dorsal surface of palatal shelf of maxilla, G12Ch201; 37= ventral longitudinal ridges long converging toward midline of vomer, G12Ch222; 38= lacrimal arches dorsally over lacrimal duct and floors lacrimal duct with medial process posteriorly, G12Ch138.

7A	1	2	3	4	5	6	7	8	9	10	11	12	13	14	15	16	17	18	19	20	21
Squamata	✓	✓x	✓x	✓X	✓	✓	✓X	✓	✓	✓	✓	✓	✓	✓	✓	✓	✓	✓	✓	✓	✓
Gekkonomorpha	✓	✓	✓	X✓	✓	✓	✓X	✓	✓	✓	✓	✓	✓	✓	✓	✓	✓	✓	✓	✓	✓
Unidentata	✓	✓x	✓x	✓	✓	✓	✓	✓	✓	✓	✓	✓	✓	✓	✓	✓	✓	✓	✓	✓	✓
Anguimorpha	✓	✓x	✓x	✓	✓	✓	✓	✓	✓	✓	✓	✓	✓	✓	✓	✓	✓	✓	✓	✓	✓
Cryptovaranoides	X	X	X	X	✓	✓	✓	✓	✓	✓	✓	✓	✓	✓	✓	✓	✓	✓	✓	✓	✓
Huehuecuetzpalli	X	✓	?	X	X	X	X	?	?	?	?	✓	✓	?	X	✓	?	?	?	?	X
Marmoretta	?	?	?	X	X	X	X	?	X	?	X	X	X	?	X	X	X	?	X	?	X
Megachirella	X	?	?	X	?	?	X	?	?	?	X	X	X	?	X	X	?	?	X	?	?
Taytalura	?	?	✓	?	X	X	X	?	X	?	?	X	X	?	?	X	?	?	?	X	?
Gephyrosaurus	X	X	X	X	X✓	X	X	X	X	X	X	X	X	?	X	X	X	?	X	✓	X

7B	22	23	24	25	26	27	28	29	30	31	32	33	34	35	36	37	38
Squamata	✓	✓	✓	✓	✓	✓	✓	✓	✓	✓	✓	✓	✓	✓	✓	✓	✓
Gekkonomorpha	X	✓	✓	✓	✓	–	✓	–	–	✓	✓	X✓	–	X	✓	X	–
Unidentata	✓	✓	✓	✓	✓	✓	✓	✓	✓	✓	✓	✓	✓	✓	✓	✓	✓
Anguimorpha	✓	✓	✓	✓	✓	✓	✓	✓	✓	✓	✓	✓	✓	✓	✓	✓	✓
Cryptovaranoides	✓	✓	✓	✓	✓	✓	✓	✓	✓	✓	✓	✓	✓	✓	✓	✓	✓
Huehuecuetzpalli	✓	✓	✓	✓	✓	✓	✓	✓	✓	✓	X	X	✓	?	?	?	?
Marmoretta	X	✓	X	?	?	X	X	X	X	✓	X	X	X	X	X	?	?
Megachirella	X	✓	X	?	X	X	?	X	?	?	X	X	?	?	?	?	?
Taytalura	?	X	X	?	?	X	X	X	X	?	X	✓	X	X	?	?	?
Gephyrosaurus	X	X	X	X	X	X	X	X	X	X	✓	✓	X	X	?	X	?

All abbreviations

Repository abbreviation: NHMUK; The Natural History Museum, Department of Earth Sciences, Cromwell Road, London SW7 5BD, UK

Anatomical abbreviations:
2nd, secondary; Ab, abducens; Aiaf, anterior inferior alveolar nerve foramen; Ad, adductor; An, angular; Ant, anterior; Art, articular; At, atlas; Ax, axis; Ba, basioccipital; Bpsr, parasphenoid rostrum; Bs, basipterygoid; Btc, trabeculae cranii; C, crest or crista; Car, carpal; Ceh, cephalic head; Cer, cervical; Ch, choana; Cl, clavicle; Cn, carina; Co, coracoid; Con, condyle; Cor, coronoid; Cr, cranial; Cra, cranii; Cv, cavity; D, digit; De, deltopectoral; Den, dentary; Dor, dorsal; Ds, dorsum sella; Dt,g, dental gutter; Ec, ectopterygoid; Ect, ectepicondyle (ectepicondylar); En, enamel; Ent, entepicondylar; Ep, epipterygoid; Er, erupting; Ex, Exoccipital; Exn, external naris; F, frontal; Fct, facet; Fe, fenestra; Fl, flange; Fo, foramen (foramina); Fos, fossa; Fov, fenestra ovalis; Fsa, foramen sacrale; Gl, glenoid; Hd, head; Hg, Hypoglossal; Hu, humerus (humeral);Hy, Hypophysial; Icf, internal carotid foramen; If, interfenestralis; In, intercentrum; Inc, interclavicle; Inv, incisive; Iptv, interpterygoid vacuity; Ju, jugal; Ke, keel; L., left; Lac, lacrimal; Lon, longitudinal; Lrst, lateral opening of the recessus scala tympani; Ltf, lower temporal fenestra; M, mandibular; Md, median; Me, medial; Mec, Meckelian; Mes, mesial; Mrst, medial opening of the recessus scala tympani; Mt, metacarpal; My, mylohyoid; Mx, maxilla (maxillary); N, nerve; Na, nasal; Nar, naris; Ne, neural; Not, notch; Oc, occipital; Oca, position of olfactory nasal capsule; Ol, olfactory; Op, opening; Orb, orbit; Oto, otooccipital; P, primary; Pa, palatine; Pacp, paroccipital process; Par, parietal; Pi, pisiform; Pg, peg; Pl, pleurodont; Plp, pleurapophysis; Pm, premaxilla; Po, postorbital; Posd, Posterodorsal trending ; Post, posterior; Pr, prootic; Pra, prearticular; Pras, processus ascendens ; Prf, prefrontal; Proc, process; Prz, prezygapophysis; Psf, postfrontal; Pt, pterygoid; Px, phalanx; Q, quadrate; R, right; Ra, radius; Rad, radiale; Rart, retroarticular; Ri, rib; Rid, ridge(d); Re, resorption; Rec, recess; S, spine; Sc, scapula; Scul, sculptured; Se, sellaris; Sm, small (smaller); So, supraorbital; Sof, suborbital fenestra; Sp, septomaxilla; Sph, sphenoid; Spl. splenial; Sq, squamosal; Stf, subtemporal fenestra; Sup, supratemporal; Sur, surangular; Sut, suture; Sy, symphysis (symphyseal); Th, tooth (teeth); Tb, tuberalis; Tu, tubera; Ty, Tympanic; Ul, ulna; Uln, ulnare; Utf, upper temporal fenestra; V, vertebra(l); Ven, ventral; Vf, Vagus foramen; Vi, vidian; Vo, vomer; Vom; vomeronasal; W, wing.

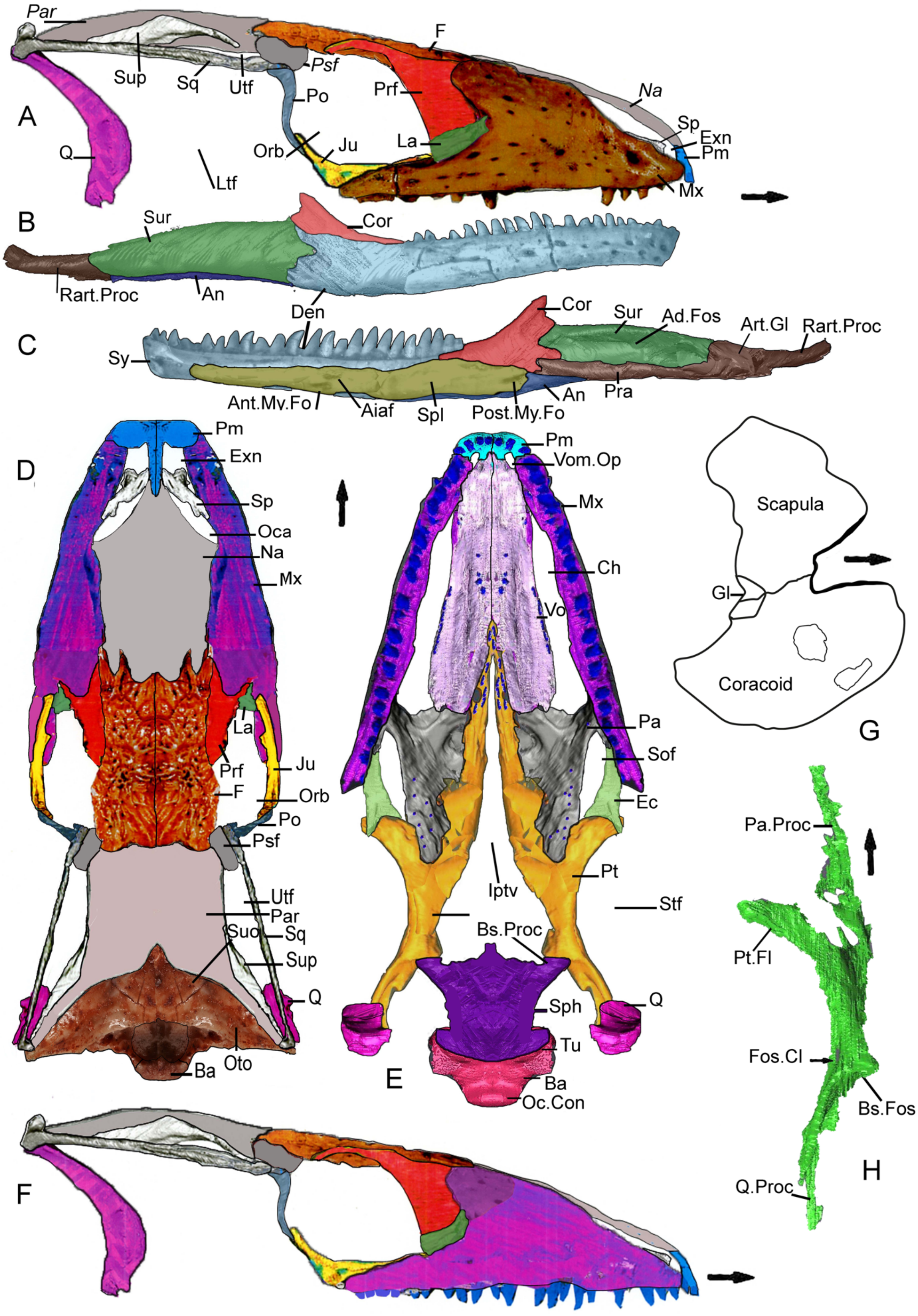

A
Par
Sup
Sq
Utf
Psf
Po
F
Prf
Na
Sp
Exn
Pm
Q
Ltf
Orb
Ju
La
Mx
B
Sur
Cor
Rart.Proc
An
Den
C
Sy
Cor
Sur
Ad.Fos
Art.Gl
Rart.Proc
Pra
Ant.My.Fo
Aiaf
Spl
Post.My.Fo
An
D
Pm
Exn
Sp
Oca
Na
Mx
La
Ju
Prf
F
Orb
Po
Psf
Utf
Par
Suo
Sq
Sup
Q
Oto
Ba
E
Pm
Vom.Op
Mx
Ch
Vo
Pa
Sof
Ec
Pt
Iptv
Stf
Bs.Proc
Sph
Q
Tu
Ba
Oc.Con
G
Scapula
Gl
Coracoid
H
Pa.Proc
Pt.Fl
Fos.Cl
Bs.Fos
Q.Proc
F

Fig. S1 [previous page]. Reconstruction of skull of *Cryptovaranoides*; pterygoid and reconstructed scapulocoracoid from CT scan. Skull shown in lateral (**A, F**), dorsal (**D**), and ventral (**E**) views; right lower jaw shown in lateral (**B**) and medial (**C**) views. Reconstruction of the skull and lower jaw based mainly on the holotype PV R 36822. **(F)** shows lateral reconstruction based on CT scanned bones only.

The reconstruction of the head bones is based on the scan images from the holotype PV R 36822 including the two halves of the skull with the left lower jaw (Fig. 1B) and the right lower jaw on the articulated upper body series (Fig. 1C, F). Additionally, isolated bones were substituted where bones are missing in the holotype or where preservation was more complete. Bone replacements are dentary PV R 37281 in B and C, and the isolated frontal PV R 37274 in A and D. The isolated coronoid PV R37273 influenced the reconstruction of B and F. The isolated basioccipital on the same rock specimen as the holotype is used in E. The composite skull length is calculated as 8 mm in the holotype and the lower jaw is of similar length, but isolated bones indicate that the skull can reach at least 2.7 cm. Note that the parietal, postfrontal and nasal are speculative as the elements are missing (or unrecognized) from the collection. However, the nasal positioning is based on facets on the frontal, maxilla and premaxilla. The parietal and nasal are shown as single elements analogously to *Varanus* but could be paired to match the frontals. (**G**) reconstructed scapulocoracoid based on CT scans of the holotype showing anterior emargination emphasized in thicker black line; the coracoid can be 10 mm wide in larger specimens. If the tentatively figured primary coracoid is excluded, the scapulocoracoid is most similar to *Heloderma* amongst living squamates (cf. fig. 13A in *59*). (**H**) Left pterygoid from CT scan of holotype to show the position of the fossa columellae where the base of the epipterygoid was positioned in life; length of pterygoid about 8mm.

Abbreviations: Aiaf, anterior inferior alveolar nerve foramen; Ad, adductor; An, angular; Ant, anterior; Art, articular; Ba, basioccipital; Bs, basipterygoid; Ch, choana; Cl, columellae; Con, condyle; Cor, coronoid; Ec, ectopterygoid; Exn, external naris; F, frontal; Fl, flange; Fo, foramen (foramina); Fos, fossa; Gl, glenoid; Iptv, interpterygoid vacuity; Ju, jugal; La, lacrimal; Ltf, lower temporal fenestra; My, mylohyoid; Mx, maxilla (maxillary); Na, nasal; Oc, occipital; Oca, position of olfactory nasal capsule; Op, opening; Orb, orbit; Oto, otooccipital; Pa, palatine; Par, parietal; Pm, premaxilla; Po, postorbital; Post, posterior; Pra, prearticular; Prf, prefrontal; Proc, process; Psf, postfrontal; Pt, pterygoid; Q, quadrate; Rart, retroarticular; Ri, rib; Rid, ridge(d); Sof, suborbital fenestra; Sp, septomaxilla; Sph, sphenoid; Spl. splenial; Sq, squamosal; Stf, subtemporal fenestra; Suo, supraoccipital; Sup, supratemporal; Sur, surangular; Sy, symphysis (symphyseal); Tu, tubercle; Utf, upper temporal fenestra; Vo, vomer; Vom; vomeronasal.

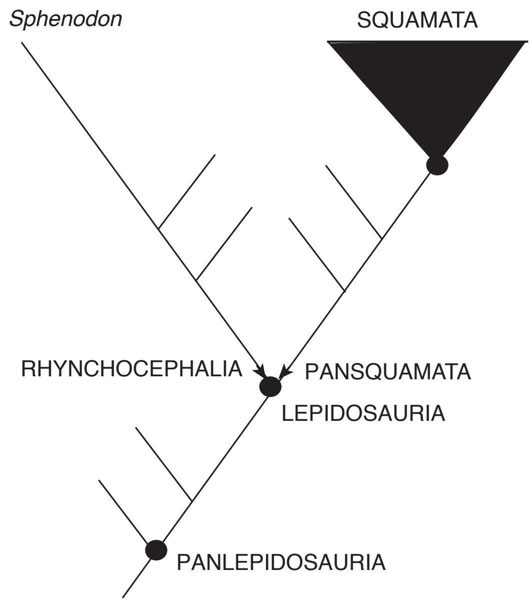

Fig. S2. Sketch cladogram, showing meanings of the terms Pan-lepidosauria, Lepidosauria, Rhynchocephalia, Pan-squamata, and Squamata. Based on refs. *12,13,60,61*.

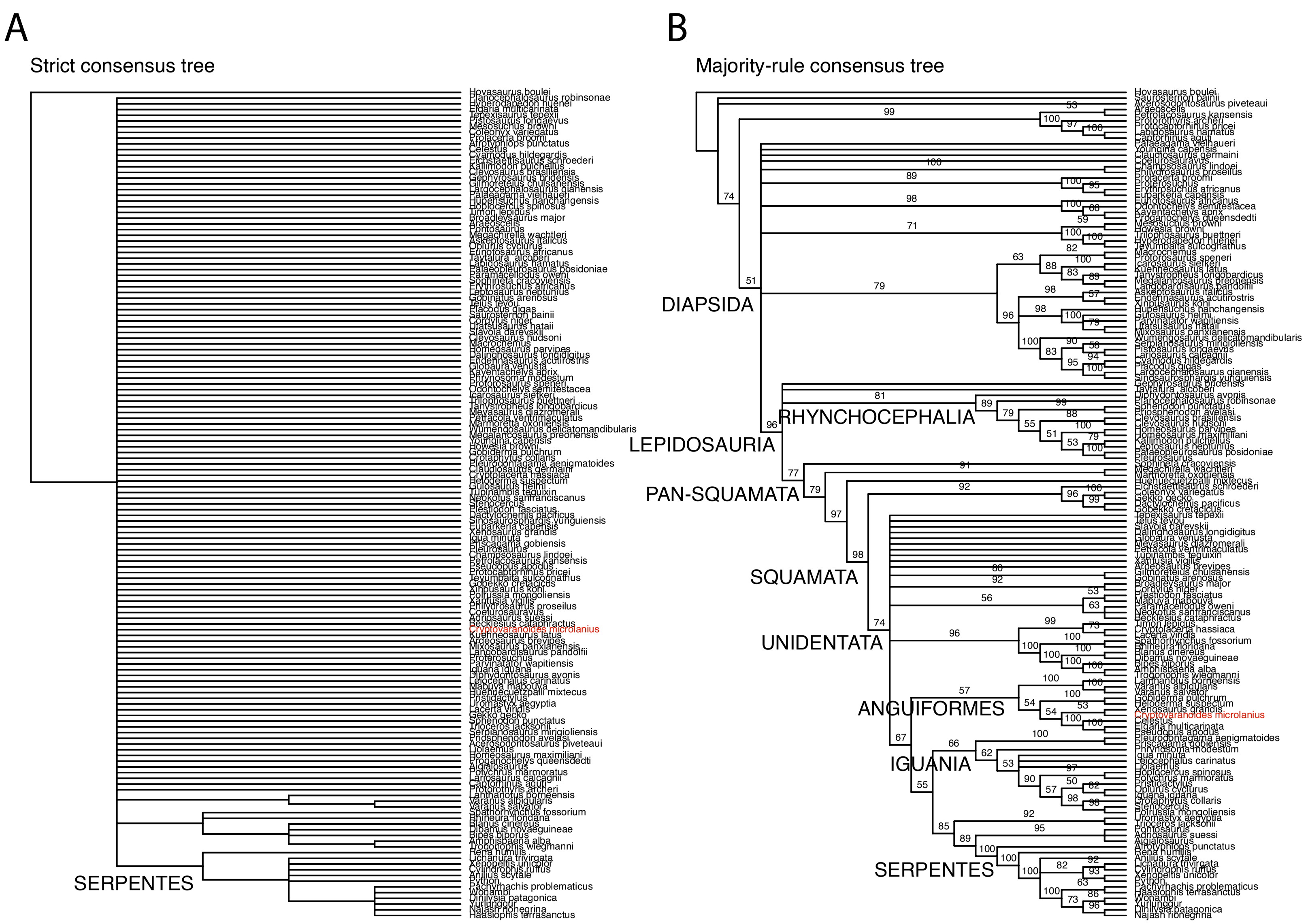
A
Strict consensus tree
SERPENTES
B
Majority-rule consensus tree
DIAPSIDA
LEPIDOSAURIA
RHYNCHOCEPHALIA
PAN-SQUAMATA
SQUAMATA
UNIDENTATA
ANGUIFORMES
IGUANIA
SERPENTES

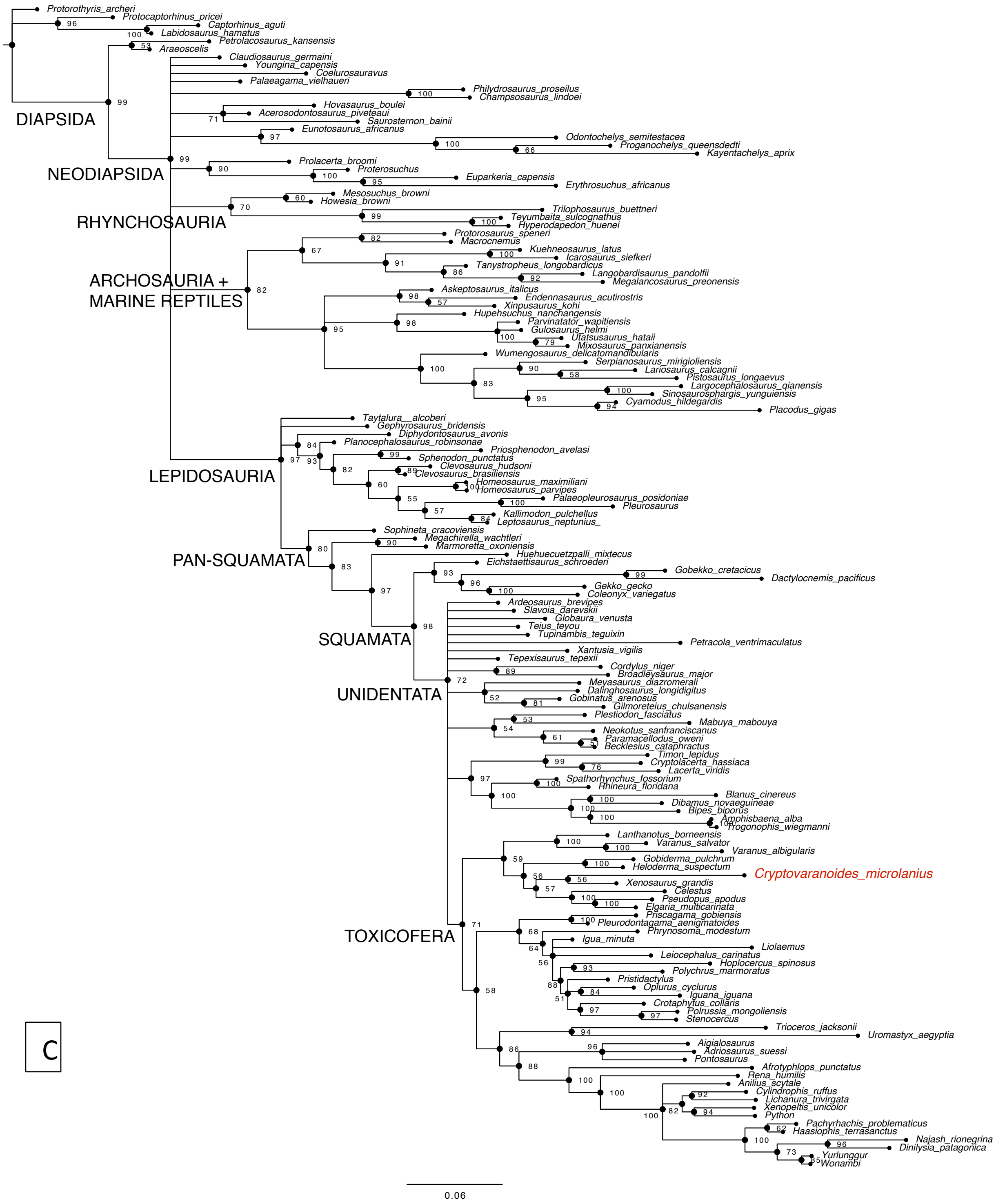

Fig. S3. Phylogenetic analyses of the phylogenetic position of *Cryptovaranoides* using the combined molecular-morphological data matrix of Martínez et al. (*32*), strict consensus tree (A), majority-rule tree (B), and maximum clade credibility tree (C) of the Bayesian analysis.

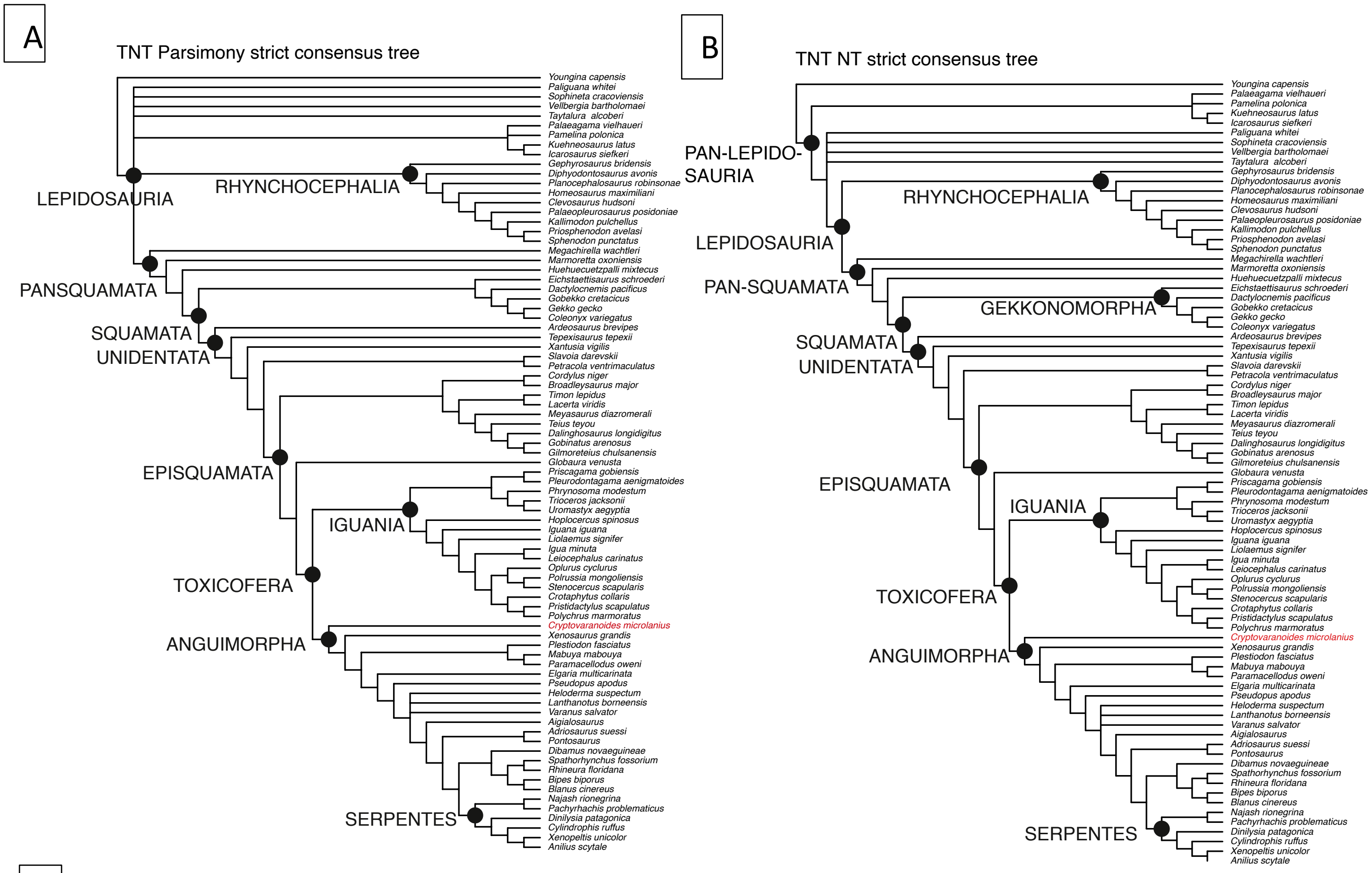

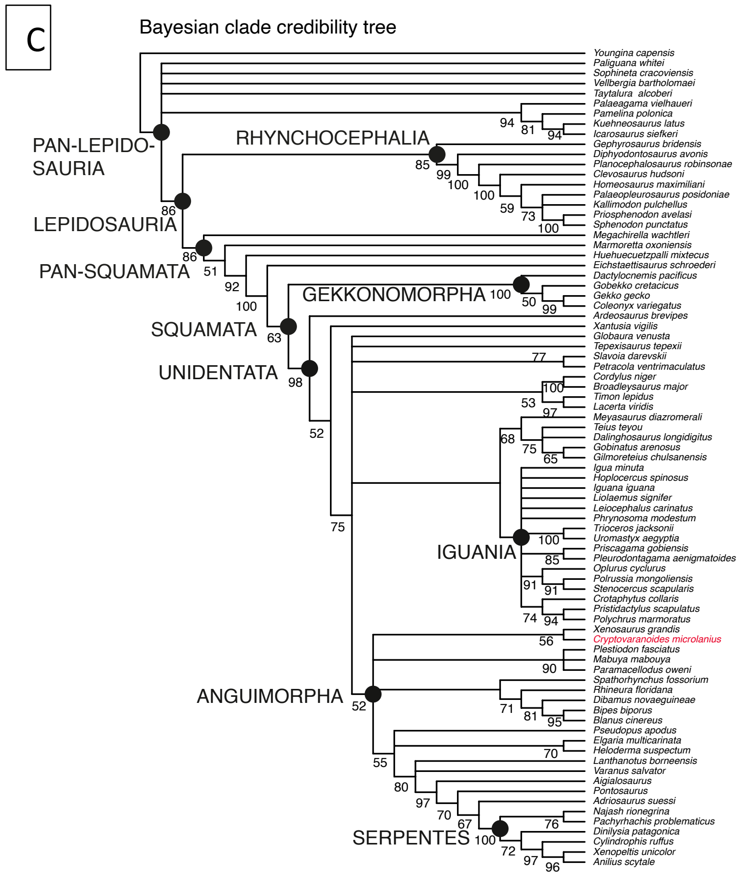

Fig. S4. Phylogenetic analyses of the phylogenetic position of *Cryptovaranoides* using the data matrix of Simões et al. (*10*), with addition of *Cryptovaranoides*, *Vellbergia* and recoding of *Megachirella* and *Marmoretta* (see recodings in Appendix S3, S4); Strict consensus tree of parsimony analysis (A); Strict consensus tree of NT analysis (B); Maximum clade credibility tree from Bayesian analysis (C).

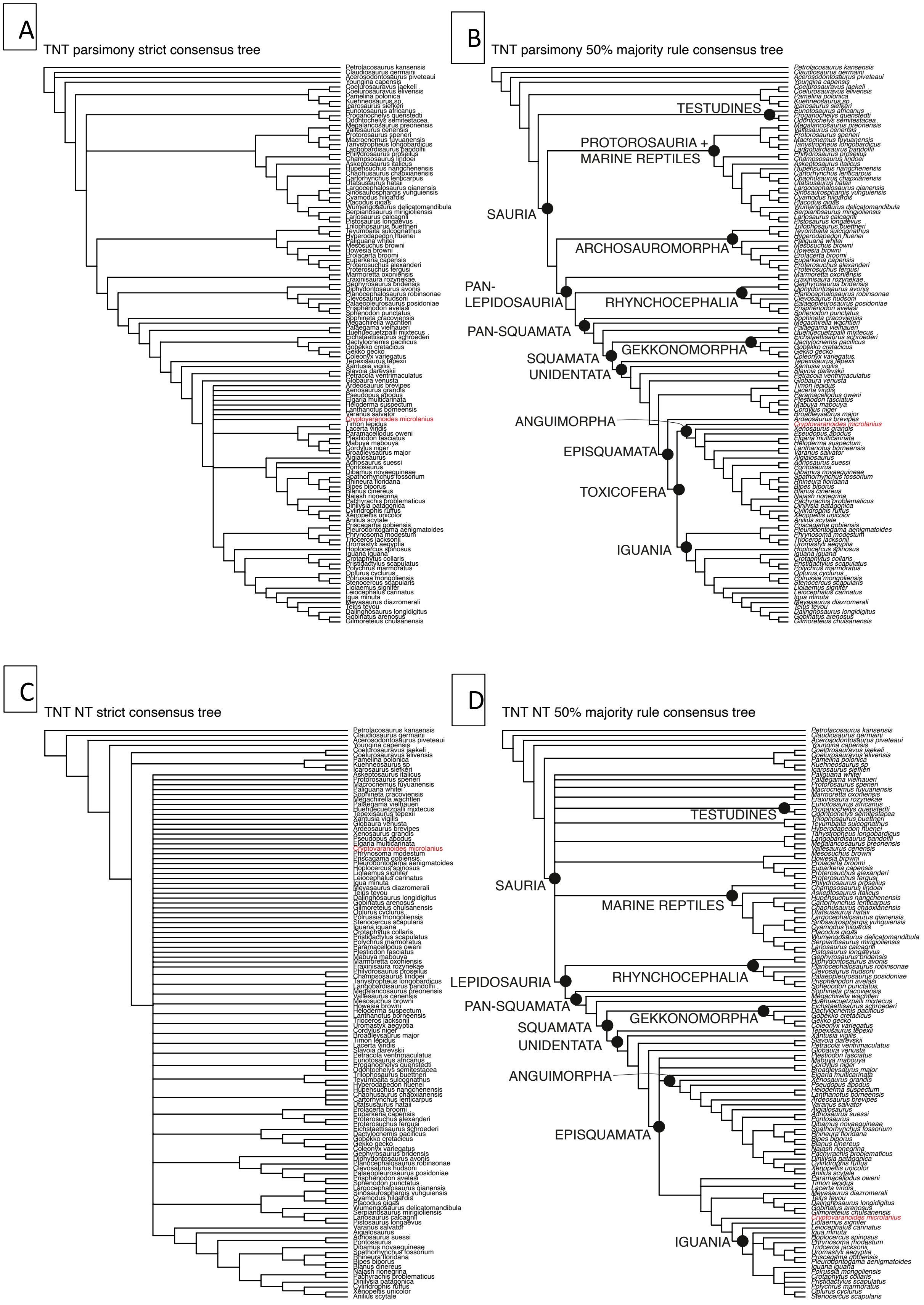
A
TNT parsimony strict consensus tree
B
TNT parsimony 50% majority rule consensus tree
TESTUDINES
PROTOROSAURIA +
MARINE REPTILES
SAURIA
ARCHOSAUROMORPHA
PAN-
LEPIDOSAURIA
RHYNCHOCEPHALIA
PAN-SQUAMATA
SQUAMATA
GEKKONOMORPHA
UNIDENTATA
ANGUIMORPHA
EPISQUAMATA
TOXICOFERA
IGUANIA
C
TNT NT strict consensus tree
D
TNT NT 50% majority rule consensus tree
TESTUDINES
SAURIA
MARINE REPTILES
LEPIDOSAURIA
RHYNCHOCEPHALIA
PAN-SQUAMATA
SQUAMATA
GEKKONOMORPHA
UNIDENTATA
ANGUIMORPHA
EPISQUAMATA
IGUANIA

Fig. S5. [previous page] Phylogenetic analyses of the phylogenetic position of *Cryptovaranoides* using the data matrix of Griffiths et al. (*11*); Strict consensus trees of parsimony analysis (A) and NT analysis (B); 50% Majority rule consensus trees of parsimony analysis (C) and NT analysis (D); Maximum clade credibility tree from Bayesian analysis (E) – below.

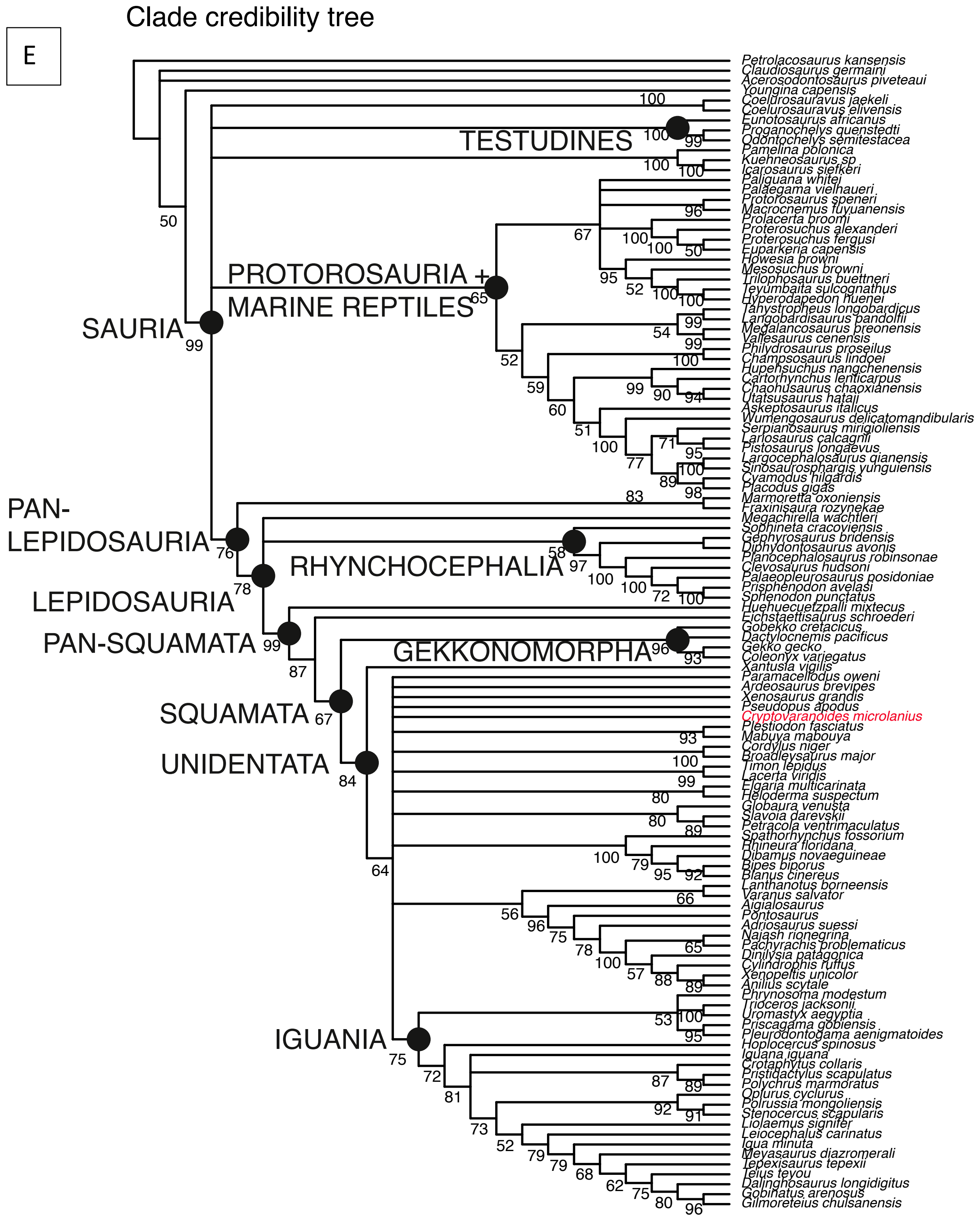

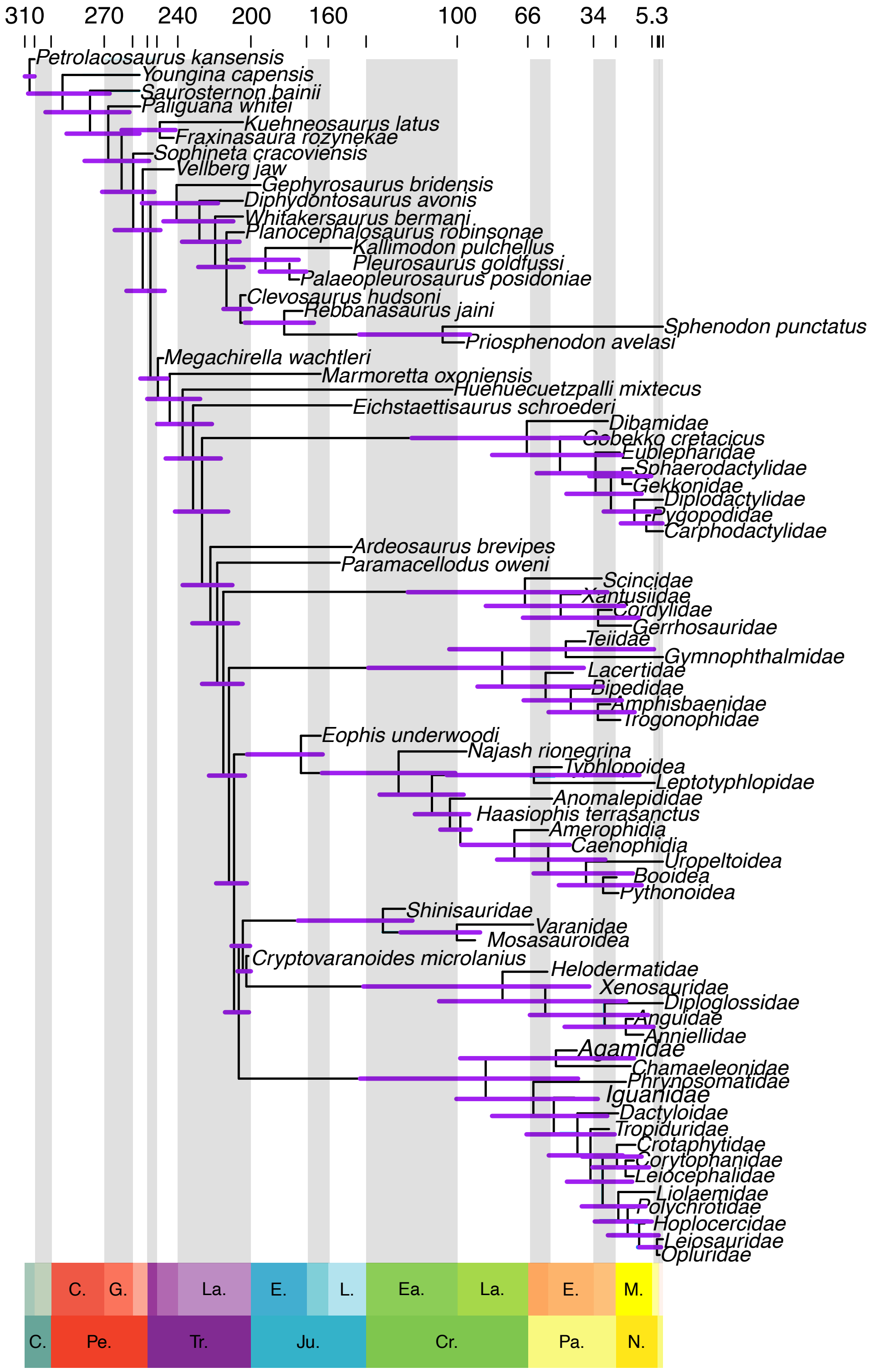

Fig. S6A. Phylogeny of Lepidosauria showing dating estimates for key clades. The tree shows all major squamate groups, constrained on a recent molecular phylogeny (*34*), and showing the effect of *Cryptovaranoides* on dating major times of divergence.

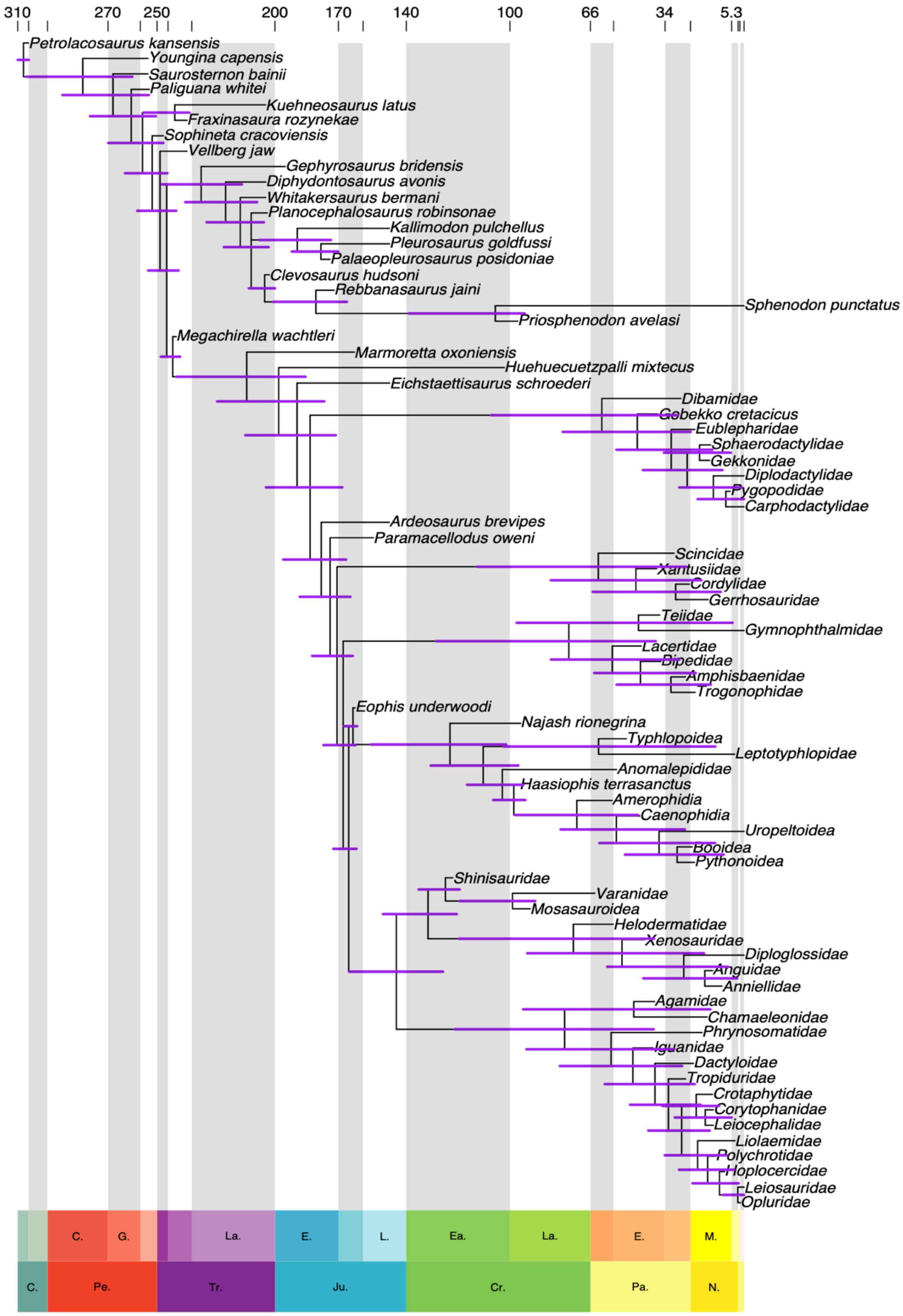

Fig. S6B. Phylogeny of Lepidosauria showing dating estimates for key clades. The tree shows all major squamate groups, constrained on a recent molecular phylogeny (*34*), but this time without *Cryptovaranoides* and showing estimated dating of major times of divergence.

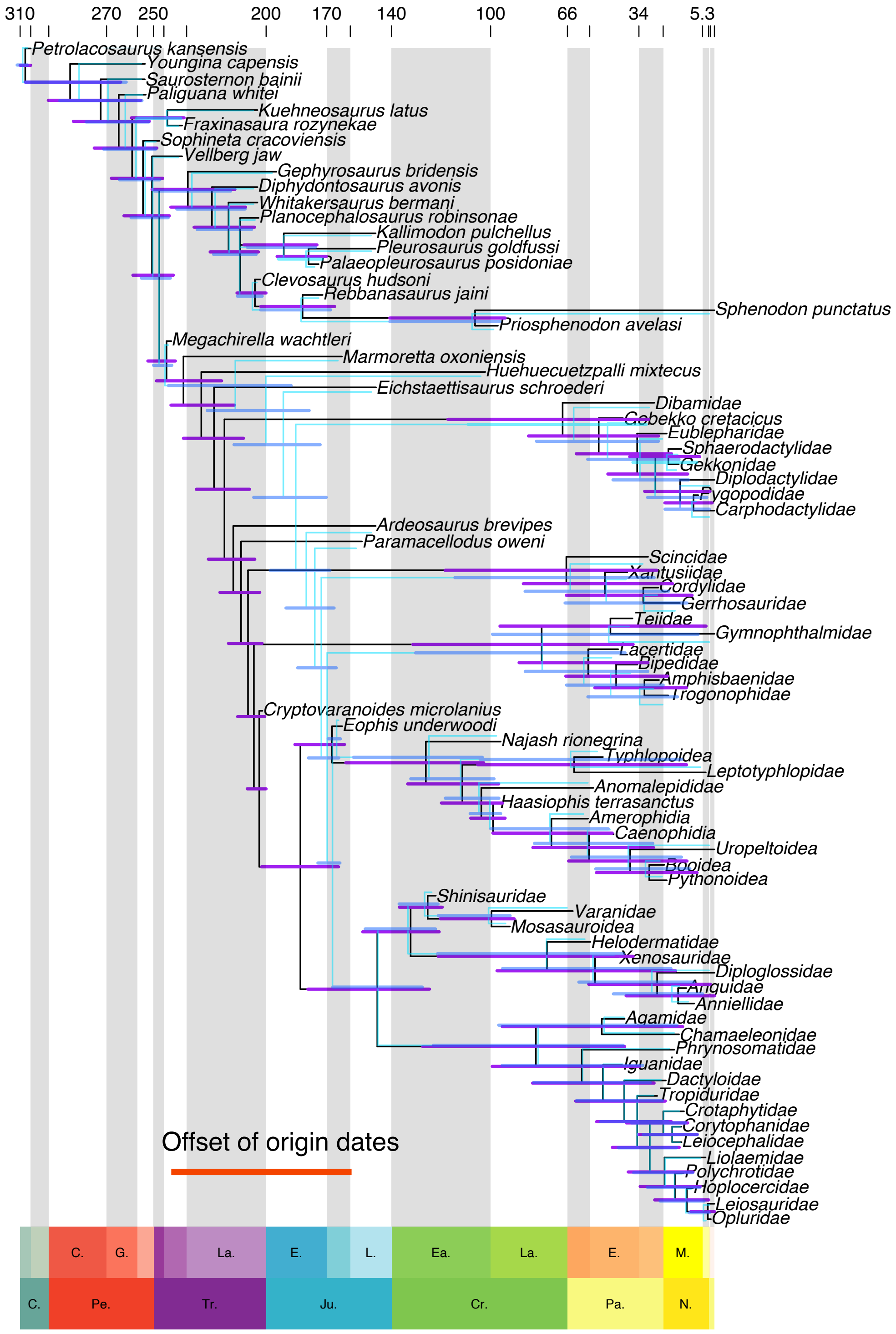

Fig. S6C. Phylogeny of Lepidosauria showing dating estimates for key clades. The tree shows all major squamate groups, constrained on a recent molecular phylogeny (*34*), and showing the difference in dating basal divergences with (older dates) and without (younger dates) the new Triassic squamate *Cryptovaranoides*.

REFERENCES AND NOTES

1. P. Uetz, J. Hošek, The reptile database (2020); www.reptile-database.org.

2. S. E. Evans, At the feet of the dinosaurs: The early history and radiation of lizards. *Biol. Rev.* **78**, 513–551 (2003).

3. L. Nessov, Late mesozoic amphibians and lizards of Soviet Middle Asia. *Acta Zool. Cracov.* **31**, 475–486 (1988).

4. P. Fedorov, L. Nessov, A lizard from the boundary of the Middle and Late Jurassic of north-east Fergana. *Bull. St Petersburg Univ. Geol. Geog.* **3**, 9–14 (1992).

5. S. E. Evans, Crown group Lizards (Reptilia, Squamata) from the Middle Jurassic of the British Isles. *Palaeontographica A* **250**, 123–154 (1998).

6. Y. Zheng, J. J. Wiens, Combining phylogenomic and supermatrix approaches, and a time-calibrated phylogeny for squamate reptiles (lizards and snakes) based on 52 genes and 4162 species. *Mol. Phylogenet. Evol.* **94**, 537–547 (2016).

7. M. E. H. Jones, C. L. Anderson, C. A. Hipsley, J. Müller, S. E. Evans, R. R. Schoch, Integration of molecules and new fossils supports a Triassic origin for Lepidosauria (lizards, snakes, and tuatara). *BMC Evol. Biol.* **13**, 208 (2013).

8. R. A. Pyron, Novel approaches for phylogenetic inference from morphological data and total-evidence dating in squamate reptiles (lizards, snakes, and amphisbaenians). *Syst. Biol.* **66**, 38–56 (2017).

9. J. L. Conrad, A new lizard (Squamata) was the last meal of *Compsognathus* (Theropoda: Dinosauria) and is a holotype in a holotype. *Zool. J. Linn. Soc.* **183**, 584–634 (2018).

10. T. R. Simões, M. W. Caldwell, M. Talanda, M. Bernardi, A. Palci, O. Vernygora, F. Bernardini, L. Mancini, R. L. Nydam, The origin of squamates revealed by a Middle Triassic lizard from the Italian Alps. *Nature* **557**, 706–709 (2018).

11. E. F. Griffiths, D. P. Ford, R. B. J. Benson, S. E. Evans, New information on the Jurassic lepidosauromorph *Marmoretta oxoniensis*. *Pap. Palaeontol.*, **7**, 2255–2278 (2021).

12. J. A. Gauthier, K. de Queiroz, Pan-Squamata, in *Phylonyms: A Companion to the PhyloCode*, K. de Queiroz, P. D. Cantino, J. A. Gauthier, Eds. (CRC Press, 2020), pp. 1087–1092.

13. K. de Queiroz, K. J. A. Gauthier, Squamata, in *Phylonyms: A Companion to the PhyloCode*, K. de Queiroz, P. D. Cantino, J. A. Gauthier, Eds. (CRC Press, 2020), pp. 1093–1101.

14. H. F. Osborn, The reptilian subclasses Diapsida and Synapsida and the early history of the Diaptosauria. *Mem. Am. Mus. Nat. Hist.* **1**, 449–507 (1903).

15. D. V. Kent, P. E. Olsen, G. Muttoni, Astrochronostratigraphic polarity time scale (APTS) for the Late Triassic and Early Jurassic from continental sediments and correlation with standard marine stages. *Earth Sci. Rev.* **166**, 153–180 (2017).

16. DigiMorph.Org. c2002–2021. Digital Morphology: A National Science Foundation Digital Library at The University of Texas at Austin; www.digimorph.org/.

17. S. E. Evans, The skull of lizards and tuatara, in *Biology of the Reptilia, Vol. 20*, C. Gans, A. S. Gaunt, K. Adler, Eds. (Society for the Study of Amphibians and Reptiles, 2008), pp. 1–347.

18. N. C. Fraser, A new rhynchocephalian from the British Upper Trias. *Palaeontology*, **25**, 709–725 (1982).

19. N. C. Fraser, The osteology and relationships of *Clevosaurus* (Reptilia: Sphenodontida). *Philos. Trans. R. Soc. B* **321**, 125–178 (1988).

20. D. I. Whiteside. The head skeleton of the Rhaetian sphenodontid *Diphydontosaurus avonis* gen. et sp. nov. and the modernizing of a living fossil. *Philos. Trans. R. Soc. B* **312**, 379–430 (1986).

21. S. E. Evans, The skull of a new eosuchian reptile from the Lower Jurassic of South Wales. *Zool. J. Linn. Soc.* **70**, 203–264 (1980).

22. J. A. Gauthier, M. Kearney, J. A. Maisano, O. Rieppel, A. Behlke, Assembling the squamate tree of life: Perspectives from the phenotype and the fossil record. *Bull. Peabody Mus. Nat. Hist.* **53**, 3–308 (2012).

23. C. Gans, R. Montero, An atlas of amphisbaenian skull anatomy, in *Biology of the Reptilia, Vol. 21*, C. Gans, A. S. Gaunt, K. Adler, Eds. (Society for the Study of Amphibians and Reptiles, 2008), pp. 621–738.

24. K. N. Bahl, Skull of *Varanus monitor* (Linn.). *Rec. Ind. Mus.* **39**, 133–174 (1937).

25. A. d'A. Bellairs, Observations of the snout of *Varanus*, and a comparison with that of other lizards and snakes. *J. Anat.* **83**, 116–146 (1949).

26. V.-H. Reynoso, *Huehuecuetzpalli mixtecus* gen. et sp. nov: A basal squamate (Reptilia) from the Early Cretaceous of Tepexi de Rodríguez, Central México. *Philos. Trans. R. Soc. B* **353**, 477–500 (1998).

27. D. I. Whiteside, C. J. Duffin, H. Furrer, The Late Triassic lepidosaur fauna from Hallau, north-eastern Switzerland, and a new 'basal' rhynchocephalian *Deltadectes elvetica* gen. et sp. nov. *Neues Jahrb. Geol. Paläontol. Abh.* **285**, 53–74 (2017).

28. J. Klembara, M. Hain, K. Dobiašová, Comparative anatomy of the lower jaw and dentition of *Pseudopus apodus* and the interrelationships of species of subfamily Anguinae (Anguimorpha, Anguidae). *Anat. Rec.* **297**, 516–544 (2014).

29. R. Hoffstetter. J. P. Gasc, Vertebrae and ribs of modern reptiles, in *Biology of the Reptilia, Vol. 1*, C. Gans, A. d'A. Bellairs, T. S. Parsons, Eds. (Academic Press, 1969), pp. 201–310.

30. J. A. Gauthier, R. Estes, K. de Queiroz, A phylogenetic analysis of the Lepidosauromorpha, in *Phylogenetic Relationships of the Lizard Families*, R. Estes, G. Pregill, Eds. (Stanford Univ. Press, 1988), pp. 15–98.

31. R. L. Cieri, S. T. Hatch, J. G. Capano, E. L. Brainerd, Locomotor rib kinematics in two species of lizards and a new hypothesis for the evolution of aspiration breathing in amniotes. *Sci. Rep.* **10**, 7739 (2020).

32. R. N. Martínez, T. R. Simões, G. Sobral, S. Apesteguía, A Triassic stem lepidosaur illuminates the origin of lizard-like reptiles. *Nature* **597**, 235–238 (2021).

33. G. Sobral, T. R. Simões, R. R. Schoch. A tiny new Middle Triassic stem-lepidosauromorph from Germany: Implications for the early evolution of lepidosauromorphs and the Vellberg fauna. *Sci. Rep.* **10**, 2273 (2020).

34. F. T. Burbrink, F. G. Grazziotin, R. A. Pyron, D. Cundall, S. Donnellan, F. Irish, J. S. Keogh, F. Kraus, R. W. Murphy, B. Noonan, C. J. Raxworthy, S. Ruane, A. R. Lemmon, E. M. Lemmon, H. Zaher, Interrogating genomic-scale data for Squamata (lizards, snakes, and amphisbaenians) shows no support for key traditional morphological relationships. *Syst. Biol.* **69**, 502–520 (2020).

35. R. A. Pyron, F. T. Burbrink, Early origin of viviparity and multiple reversions to oviparity in squamate reptiles. *Ecol. Lett.* **17**, 13–21 (2014).

36. J. L. Conrad, Phylogeny and systematics of Squamata (Reptilia) based on morphology, *Bull. Am. Mus. Nat. Hist.* **310**, 1–182 (2008).

37. J. D. Corso, M. Bernardi, Y. D. Sun, J. D. Corso, M. Bernardi, Y. Sun, H. Song, L. J. Seyfullah, N. Preto, P. Gianolla, A. Ruffell, E. Kustatscher, G. Roghi, A. Merico, S. Hohn, A. R. Schmidt, A. Marzoli, R. J. Newton, P. B. Wignall, M. J. Benton, Extinction and dawn of the modern world in the Carnian (Late Triassic). *Science Adv.* **6**, eaba0099 (2021).

38. Z.-Q. Chen, M. J. Benton, The timing and pattern of biotic recovery following the end-Permian mass extinction. *Nat. Geosci.* **5**, 375–383 (2012).

39. S. A. V. Chambi-Trowell, D. I. Whiteside, M. J. Benton, Diversity in rhynchocephalian *Clevosaurus* skulls based on CT reconstruction of two Late Triassic species from Great Britain. *Acta Palaeontol. Pol.* **64**, 41–64 (2019).

40. D. I. Whiteside, C. J. Duffin, P. G. Gill, J. E. A. Marshall, M. J. Benton, The Late Triassic and Early Jurassic fissure faunas from Bristol and South Wales: Stratigraphy and setting. *Palaeontol. Pol.* **67**, 257–287 (2016).

41. T. J. Cleary, R. B. J. Benson, S. E. Evans, P. M. Barrett, Lepidosaurian diversity in the Mesozoic–Palaeogene: The potential roles of sampling biases and environmental drivers. *R. Soc. Open Sci.* **5**, 171830 (2018).

42. R. E. Ricklefs, J. B. Losos, T. M. Townsend, Evolutionary diversification of clades of squamate reptiles. *J. Evol. Biol.* **20**, 1751–1762 (2007).

43. G. T. Lloyd, K. E. Davis, D. Pisani, J. E. Tarver, M. Ruta, M. Sakamoto, D. W. E. Hone, R. Jennings, M. J. Benton, Dinosaurs and the Cretaceous terrestrial revolution. *Proc. R. Soc. B* **275**, 2483–2490 (2008).

44. S. M. Harrington, T. W. Reeder, Phylogenetic inference and divergence dating of snakes using molecules, morphology and fossils: New insights into convergent evolution of feeding morphology and limb reduction. *Biol. J. Linn. Soc.* **121**, 379–394 (2017).

45. A. Y. Hsiang, D. J. Field, T. H. Webster, A. D. B. Behlke, M. B. Davis, R. A. Racicot, J. A. Gauthier, The origin of snakes: Revealing the ecology, behavior, and evolutionary history of early snakes using genomics, phenomics, and the fossil record. *BMC Evol. Biol.* **15**, 87 (2015).

46. M. J. Benton, P. Wilf, H. Sauquet, The angiosperm terrestrial revolution and the origins of modern biodiversity. *New Phytol.* **233**, 2017–2035 (2022).

47. P. L. Robinson, The Mesozoic fissures of the Bristol Channel area and their vertebrate faunas. *Zool. J. Linn. Soc.* **43**, 260–282 (1957).

48. A. O'Brien, D. I. Whiteside, J. E. A. Marshall, Anatomical study of two previously undescribed specimens of *Clevosaurus hudsoni* (Lepidosauria: Rhynchocephalia) from Cromhall Quarry, UK, aided by computed tomography, yields additional information on the skeleton and hitherto undescribed bones. *Zool. J. Linn. Soc.* **183**, 163–195 (2018).

49. J. Lovegrove, A. J. Newell, D. I. Whiteside, M. J. Benton, Testing the relationship between marine transgression and evolving island palaeogeography using 3D GIS: An example from the Late Triassic of SW England. *J. Geol. Soc. London* **178**, jgs2020-158 (2020).

50. J. D. Morton, D. I. Whiteside, M. Hethke, M. J. Benton, Biostratigraphy and geometric morphometrics of conchostracans (Crustacea, Branchiopoda) from the Late Triassic fissure deposits of Cromhall Quarry, UK. *Palaeontology* **60**, 349–374 (2017).

51. F. Ronquist, M. Teslenko, P. van der Mark, D. L. Ayres, A. Darling, S. Höhna, B. Larget, L. Liu, M. A. Suchard, J. P. Huelsenbeck, MrBayes 3.2: Efficient Bayesian phylogenetic inference and model choice across a large model space. *Syst. Biol.* **61**, 539–542 (2012).

52. P. A. Goloboff, J. S. Farris, K. C. Nixon, TNT, a free program for phylogenetic analysis. *Cladistics* **24**, 774–786 (2008).

53. T. R. Simões, M. W. Caldwell, S. E. Pierce, Sphenodontian phylogeny and the impact of model choice in Bayesian morphological clock estimates of divergence times and evolutionary rates. *BMC Biol.* **18**, 191 (2020)

54. R. J. G. Savage, Vertebrate fissure faunas with special reference to Bristol Channel Mesozoic faunas. *J. Geol. Soc. London* **150**, 1025–1034 (1993).

55. G. M. Walkden, N. C. Fraser, Late Triassic fissure sediments and vertebrate faunas: Environmental change and faunal succession at Cromhall South West Britain. *Mod. Geol.* **18**, 511–535 (1993).

56. D. I. Whiteside, J. E. A. Marshall, The age, fauna and palaeoenvironment of the Late Triassic fissure deposits of Tytherington, South Gloucestershire UK. *Geol. Mag.* **145**, 102–147 (2008).

57. G. M. Walkden, N. C. Fraser, M. J. Simms, The age and formation mechanisms of Late Triassic fissure deposits, Gloucestershire, England: Comments on G. Mussini, D. I. Whiteside, C. Hildebrandt, M. J. Benton, Anatomy of a Late Triassic Bristol fissure: Tytherington fissure 2. *Proc. Geol. Assoc.* **132**, 127–137 (2021).

58. D. I. Whiteside, M. J. Benton, The age and formation mechanisms of Late Triassic fissure deposits, Gloucestershire, England: Comments on G. Mussini, D. I. Whiteside, C. Hildebrandt, M. J. Benton, *Proc. Geol. Assoc.* **132**, 138–141 (2021).

59. R. Estes, K. de Queiroz, J. A. Gauthier, Phylogenetic relationships within Squamata, in *Phylogenetic Relationships of the Lizard Families,* R. Estes, G. Pregill, Eds. (Stanford Univ. Press, 1988), pp. 119–281.

60. J. A. Gauthier, K. de Queiroz, Pan-Lepidosauria, in *Phylonyms: A Companion to the PhyloCode*, K. de Queiroz, P. D. Cantino, J. A. Gauthier, Eds. (CRC Press, 2020), pp. 1075–1078.

61. K. de Queiroz, J. A. Gauthier, Lepidosauria, in *Phylonyms: A Companion to the PhyloCode,* K. de Queiroz, P. D. Cantino, J. A. Gauthier, Eds. (CRC Press, 2020), pp. 1079–1085.

62. M. S. Y. Lee, Convergent evolution and character correlation in burrowing reptiles: Towards a resolution of squamate relationships. *Biol. J. Linn. Soc.* **65**, 369–453 (1998).

63. R. R. Schoch, H.-D. Sues. A new lepidosauromorph reptile from the Middle Triassic (Ladinian) of Germany and its phylogenetic relationships. *J. Vertebr. Paleontol.* **38**, e1444619 (2018).

64. E. D. Cope, On the reptilian orders Pythonomorpha and Streptosauria. *Proc. Boston Soc. Nat. Hist.* **12**, 250–266 (1869).

65. S. Renesto, R. Posenato, R. A new lepidosauromorph reptile from the Middle Triassic of the Dolomites (Northern Italy). *Riv. Ital. Paleontol. Stratigr.* **109**, 463–474 (2003).

66. S. Renesto, M. Bernardi, Redescription and phylogenetic relationships of *Megachirella wachtleri* Renesto et Posenato, 2003 (Reptilia, Diapsida). *Paläontol. Z.* **88**, 197–210 (2014).

67. D. I. Whiteside, C. J. Duffin, Late Triassic terrestrial microvertebrates from Charles Moore's 'Microlestes' quarry, Holwell, Somerset, UK. *Zool. J. Linn. Soc.* **179**, 677–705 (2017).

68. M. E. H. Jones, N. Curtis, M. J. Fagan, P. O'Higgins, S. E. Evans, Hard tissue anatomy of the cranial joints in *Sphenodon* (Rhynchocephalia): Sutures, kinesis, and skull mechanics. *Palaeontol. Electron.* **14**, 1–92 (2011).

69. S. E. Evans, A new lizard-like reptile (Diapsida: Lepidosauromorpha) from the Middle Jurassic of England. *Zool. J. Linn. Soc.* **103**, 391–412 (1991).

70. M. Waldman, S. E. Evans, Lepidosauromorph reptiles from the Middle Jurassic of Skye. *Zool. J. Linn. Soc.* **112**, 135–150 (1994).
